# The reactivity of coordinated and uncoordinated chromate ions in the quasi-intramolecular redox reactions of [pentaamminechromatocobalt(iii)] chloride and chromate resulting in tuneable cobalt-chromium spinel oxide catalysts

**DOI:** 10.1039/d6ra06540f

**Published:** 2026-10-23

**Authors:** Bálint Tulik, Zsolt Dürvanger, Shiro Kubuki, Attila Farkas, Stjepko Krehula, Vladimir M. Petruševski, Laura Bereczki, Zoltán Homonnay, Milica Marković, Aleksandra Rakic, Dušan Dimić, László Trif, Zoltán Károly, László Kótai, Kende Attila Béres

**Affiliations:** a Institute of Materials and Environmental Chemistry, HUN-REN Research Centre for Natural Sciences Budapest Hungary tulik.balint@ttk.hu beres.kende.attila@ttk.hu bereczki.laura@ttk.hu trif.laszlo@ttk.hu karoly.zoltan@ttk.hu kotai.laszlo@ttk.hu; b Department of Physical Chemistry and Materials Science, Faculty of Chemical Technology and Biotechnology, Budapest University of Technology and Economics Budapest Hungary; c Laboratory of Structural Chemistry and Biology, Institute of Chemistry, ELTE Eötvös Loránd University Budapest Hungary zsolt.durvanger@ttk.elte.hu; d HUN-REN-ELTE Protein Modelling Research Group Budapest Hungary; e Department of Chemistry, Graduate School of Science, Tokyo Metropolitan University 1-1 Minami-Osawa, Hachi-Oji Tokyo 192-0397 Japan kubuki@tmu.ac.jp; f Department of Organic Chemistry and Technology, Faculty of Chemical Technology and Biotechnology, Budapest University of Technology and Economics Budapest Hungary farkas.attila@vbk.bme.hu; g Division of Materials Chemistry, Ruđer Bošković Institute Zagreb Croatia Stjepko.Krehula@irb.hr; h Institute of Chemistry, Faculty of Natural Sciences and Mathematics, Ss. Cyril and Methodius University Skopje Republic of North Macedonia vladimirpetrusevski@yahoo.com; i Institute of Chemistry, ELTE Eötvös Loránd University Budapest Hungary homonnay.zoltan@ttk.elte.hu; j Faculty of Physical Chemistry, University of Belgrade Belgrade Serbia milica@bio.bg.ac.rs saska@ffh.bg.ac.rs dusan.dimic@ffh.bg.ac.rs

## Abstract

The synthesis routes, crystal and molecular structures, and controlled thermal decomposition reactions of two [pentamminecobalt(iii) compounds having a coordinated chromate ion, [Co(NH_3_)_5_CrO_4_]Cl (compound 1) and [Co(NH_3_)_5_CrO_4_]_2_CrO_4_ (compound 2) were studied experimentally. Quantum chemical optimizations and QTAIM calculations were performed for compounds 1 and 2, and the selected Bond Critical Points (BCPs) properties were discussed. The extended hydrogen-bond network between the coordinated chromate ions and ammonia ligands acts as reactive redox reaction centres, and both compounds show solid-phase quasi-intramolecular redox reactions with the formation of Co[Co_0.5_Cr_1.5_O_3.75_] and Co[Co_0.2_Cr_1.8_O_3.90_] nanosized regular spinels with nominal Co : Cr = 1 : 1 and 2 : 3 molar ratios, respectively. The tetrahedral positions of these spinels are filled with Co^II^ ions. The octahedral positions of 2 : 3 and 1 : 1 spinels prepared in air are filled Cr^III^ and mainly with Co^III^ ions. The 2 : 3 spinel prepared under N_2_ shows a similar distribution of ions, whereas the 1 : 1 spinel prepared under N_2_ contains only Co^II^ ions at T-4, and Cr^III^, Co^III^, and Co^II^ ions at the OC-6 positions, resulting in a strongly defective structure. The position of the chromate ions (inner-sphere coordinated or outer-sphere non-coordinated) plays an important role in the quasi-intramolecular redox reactions and thermal decomposition characteristics of compounds 1 and 2. The inner-sphere coordinated chromate ions of both compounds took part in redox reactions with their own coordinated ammonia only after the loss of the fifth ammonia, in an endothermic ligand-releasing and an exothermic redox reaction mediated by the outer-sphere chromate ion for compounds 1 and 2, respectively. The cobalt(iii) content of both complexes is also reduced completely to cobalt(ii). These spinels were proven to be active catalysts in the visible-light-driven photocatalytic oxidation of rhodamine B with H_2_O_2_.

## Abstract 2

The synthesis routes, structures, and thermal decomposition reactions of two [pentamminecobalt(iii) compounds having a coordinated chromate ion, [Co(NH__3__)__5__CrO__4__]Cl (compound 1) and [Co(NH__3__)__5__CrO__4__]__2__CrO__4__ (compound 2) were studied experimentally.

## Graphical abstract

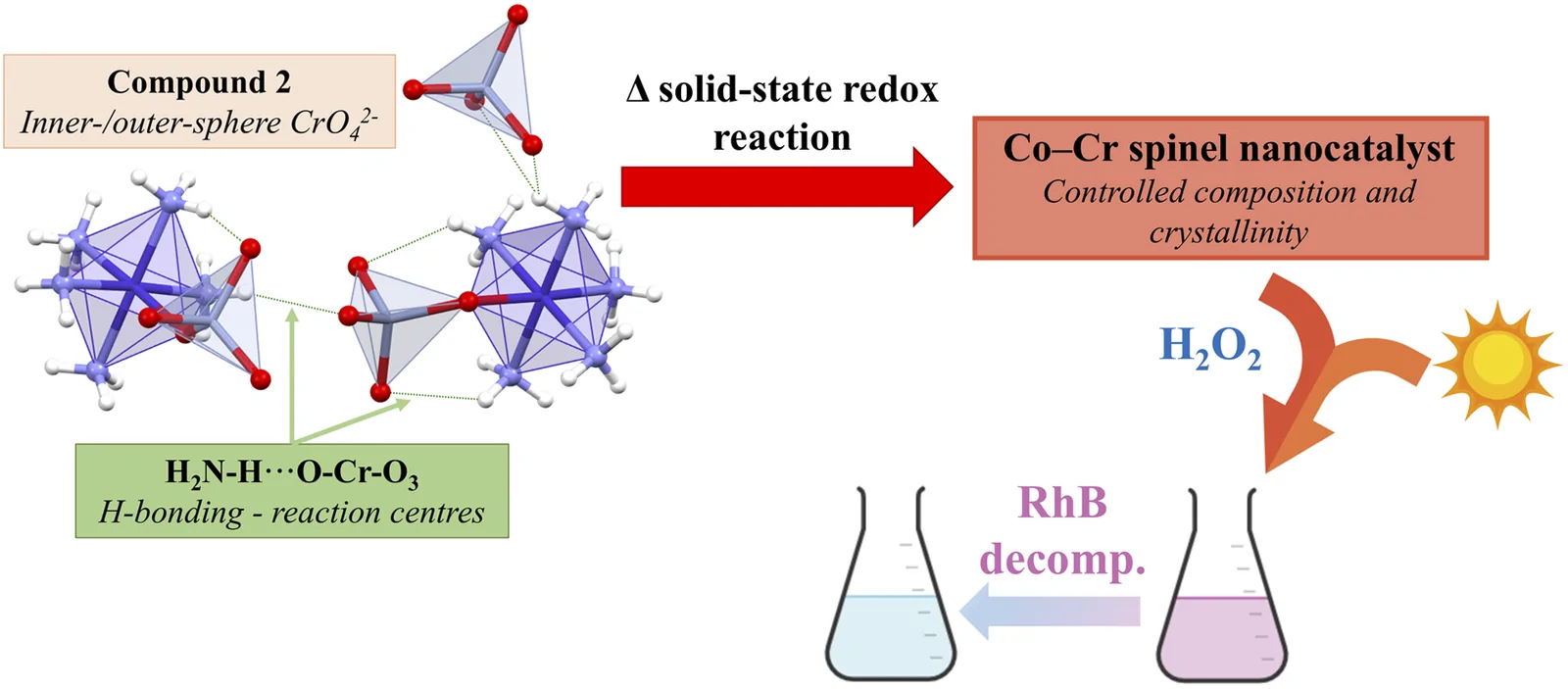

## Introduction

The solid-phase quasi-intramolecular redox reactions (SPQIR) of a unique group of coordination compounds having reducing ligands and oxidizing anions proved to be an effective tool for preparing and tuning nanosized mixed-oxide catalysts.^1,2^ The SPQIR offers great flexibility in the preparation of mixed oxides at relatively low temperature with respect to composition, lattice structure, distribution of defects, metal ions, and valences in lattice positions. In this way, uncommon, non-equilibrium mixed spinel oxide structures of Co^II,III^, Fe^II,III^, Cr^III^, and Mn^II,III^ were prepared from Fe and Co complexes containing permanganate or dichromate anions.^3–5^ These mixed oxides were found to be active catalysts in various environmentally important processes, including CO_2_ reduction,^6^ therefore, we started systematic studies on the preparation and thermal decomposition of [ML_*n*_](XO_*a*_)_*b*_ (where M is one or more metals, L is one or more ligands, X is one or more metal or non-metallic elements, and *a* and *b* are in the range from 0–6) complexes to find the best precursors for optimal catalyst preparation for some selected industrial processes. The variability of catalyst composition and the tuning of these material properties *via* SPQIR is almost infinite, with the selection of precursors by two basic principles:

• By pairing each complex cation, ligand, and anion with various valences, coordination numbers, and coordination modes, making isomorphous solid solutions,^7^ or mixed anionic salts,^8,9^ especially by the incorporation of the same metal from both cationic and anionic positions (in various valences of the precursor compound) and adjusting their ratios, such as in [Cr(urea)_6_](Cr_2_O_7_)(X) or [Cr(urea)_6_(CrO_4_)X salts^8^ (X = oxidizing or non-oxidizing anion), or partial incorporation of non-metal isomorphous or co-anion/ligand, which gives gaseous or water-soluble decomposition products for tuning of cationic/anionic metal ratios in the synthesized oxides, such as [Co(NH_3_)_4_CO_3_]MnO_4_,^10^ [Co(NH_3_)_6_]Cl_2_(MnO_4_)_2_,^11^ [Co(NH_3_)_6_]Br_2_MnO_4_ (ref. 12) or [Co(NH_3_)_5_Cl](MnO_4_)_2_ (ref. 13) complexes.

• By changing the chemical nature of the same metal-containing fragment, *e.g.*, coordinated or non-coordinated nature of the same oxidizing anion as in the complexes of [Co(NH_3_)_5_CrO_4_]X or [Co(NH_3_)_5_X]_*n*_CrO_4_, when X may also be an oxidizing or non-oxidizing anion.^14,15^

The cobalt-chromium spinel oxides (cochromites^16,17^) are cost-effective and sustainable alternatives to precious metal-based catalysts in various processes^18^ with high thermal stability,^19^ good redox properties, and a wide band gap,^20–22^ making them ideal for environmental applications.^23^ They are abundant, less capable of deactivation,^24^ efficient, and long-term photocatalysts^25^ in wastewater treatment.^26–28^ The cochromite inverse spinels were found to be highly active catalysts in the decomposition of H_2_O_2_ into hydroxyl radicals.^29^ The cobalt(ii) ions located in octahedral sites were believed to be responsible for this activity, due to the negative charge excess in the centre of [Co^II^O_6_] octahedra, whereas cobalt(ii) ions located at tetrahedral sites (regular CoCr_2_O_4_) have no excess negative charge and activity.^30,31^ From this point of view, the Co–Cr mixed oxides prepared in SPQIR, especially those with a Cr : Co ratio <2 and a large amount of Co^II^ at octahedral sites, are potential catalyst candidates. It is well known that hydrogen peroxide oxidizes chromium(iii) to chromium(vi), and the chromate ion can react with H_2_O_2_ to form peroxochromate species.^32^ These peroxide intermediates are coloured materials – they absorb visible light and are expected to be excited to a reactive state; thus, it seems to be a promising route for a visible-light-driven degradation of hardly decomposable organic dyes. With the aim of the preparation of precursors of mixed (Co,Cr)O_*x*_ oxides with a Cr to Co ratio <2 ratio with precious catalytic properties, in our present work, we prepared and studied in detail the [Co(NH_3_)_5_CrO4]Cl (compound 1) and [Co(NH_3_)_5_CrO_4_]_2_CrO_4_ (compound 2) complexes, using single crystal XRD, IR, Raman and UV spectroscopies, thermal analysis (TG-MS and DSC), SXRD, PXRD, cryo-DSC, and DFT methods. The influence of chromate coordination (or non-coordination) and O/Cr ratio on the properties of the SPQIR-synthesized Co–Cr mixed oxides (cochromites) from compounds 1 and 2 was compared with the help of PXRD, IR, BET, SEM, XPS, XANES, EXAFS, and visible-light-driven photocatalytic rhodamine B degradation methods.

## Results and discussion

### Synthesis of compounds 1 and 2

Compound 2 was prepared first by Braun^33^ in 1863, by dissolution of solid [Co(NH_3_)_5_(H_2_O)]X_3_ salts (X = Cl and NO_3_, roseocobaltic chloride and nitrate, respectively) in concentrated potassium chromate solutions. The use of [Co(NH_3_)_5_Cl]Cl_2_ (purpureocobaltic chloride) resulted in a mixture of compound 2 and a chloride- and chromate-containing material. Its composition was identical to that of the salt isolated by Jörgensen^34^ in the reaction of a cooled potassium chromate and [pentaamminecobalt(iii)] compound (chloride, sulphate, or nitrate) solution, however, their properties (colour, chemical reactions) were very different. Coomber^14^ identified Jörgensen's compound as [Co(NH_3_)_5_Cl]CrO_4_ (compound 3); the compound found by Braun^33^ may be supposed to be the other isomer, [Co(NH_3_)_5_CrO_4_]Cl (compound 1). This assumption is strengthened by the well-known reaction of [Co(NH_3_)_5_(H_2_O)]^3+^ salts, *e.g.*, the perchlorate compound's reaction with 1 equivalent of K_2_CrO_4_ at pH = 6.5, with the formation of [Co(NH_3_)_5_CrO_4_]ClO_4_.^35^ Compound 1 was prepared in a pure state by Briggs in the reaction of hot K_2_CrO_4_ and [Co(NH_3_)_5_Cl]Cl_2_ solutions, and it could be transformed into compound 2 by reacting with Ag_2_CrO_4_.^36^

The products of the reaction between [Co(NH_3_)_5_Cl]Cl_2_ and K_2_CrO_4_ depend strongly on the molar ratio and concentrations of the reactants, as well as the reaction temperature. The outcome of the reaction is strongly affected by the hydrolysis of [Co(NH_3_)_5_Cl]^2+^ core with the formation of [aquapentamminecobalt(iii)] cation under acidic conditions^37,38^ and, at low pH values, by the appearance of dichromate ions. The hydrolysis reactions of [Co(NH_3_)_5_Cl]^2+^ complexes are relatively slow and pH-dependent processes,^39,40^ thus, over the pH range, the reaction time and the solubility of the potential chromate/dichromate products also affect the distribution of the products. The positions of chloride and chromate anions in the spectrochemical^41^ and polarographic stability^42^ series of [Co(NH_3_)_5_X]^*n*+^ salts are very close to each other; their coordination ability to cobalt in these complexes might be similar. Therefore, the Cl^−^/CrO_4_^2−^ exchange in the coordination sphere to prepare chromate-coordinated species requires careful selection of the appropriate reaction conditions (Scheme 1).

**Scheme 1:**
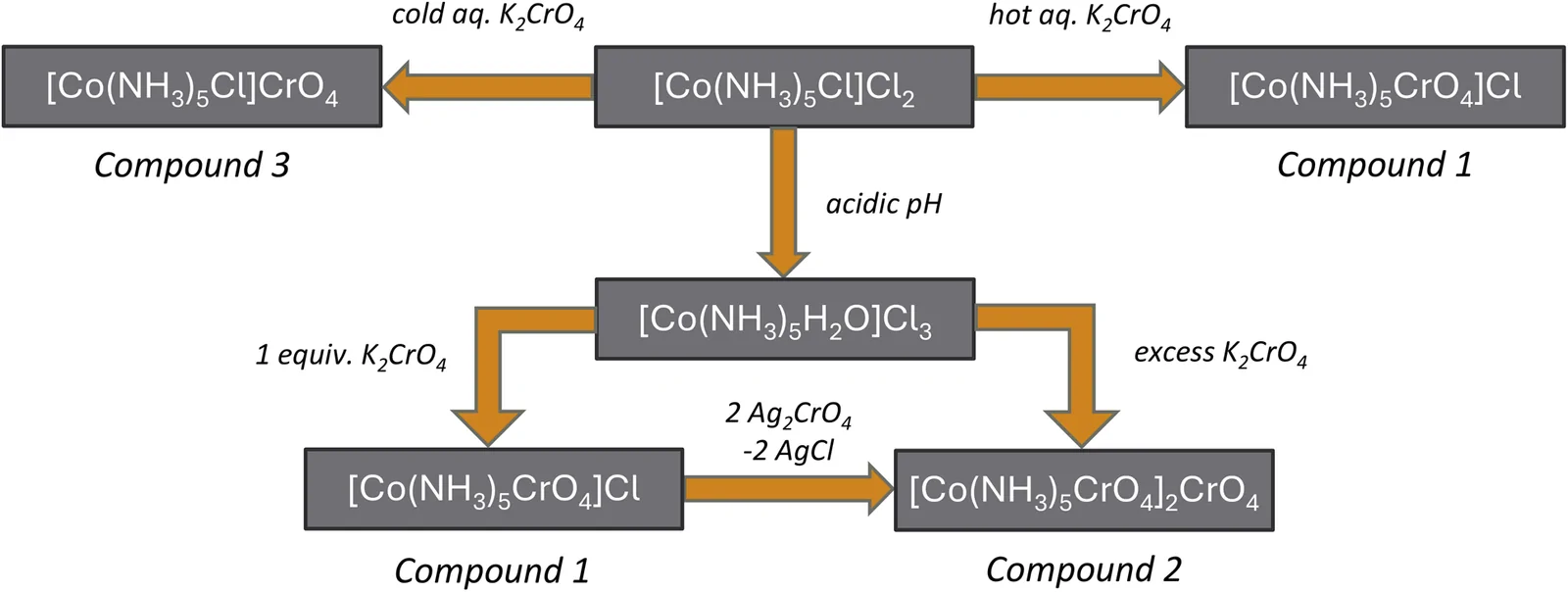
The possible synthesis routes of compounds 1–3.

To prepare chromate-coordinated compounds 1 and 2, we selected the reaction routes applied by Briggs, with slight modifications. A [Co(NH_3_)_5_Cl]Cl_2_ solution (pH = 3.5) at 70 °C was reacted with hot K_2_CrO_4_ solution to isolate compound 1 (at pH = ∼6), then the exchange of the non-coordinated chloride ion in compound 1 with silver chromate resulted in the formation of compound 2 and insoluble AgCl (Scheme 1).

The isolated two chromate-coordinated products, compounds 1 and 2, were formed as reddish-brown needle-like mono- and dark brown tetrahydrates, [Co(NH_3_)_5_CrO_4_]Cl·H_2_O (compound 1) and [Co(NH_3_)_5_CrO_4_]_2_CrO_4_·4H_2_O (compound 2), respectively. Their solubility, pH, and electrolytic conductivities of their saturated aqueous solutions were found to be 5.824 and 2.480 g L^−1^, 5.96 and 5.23, and 1.894 and 0.615 mS, respectively. The pH and electrolytic conductivities of their 10-fold diluted solutions (all of them at 23 °C) were 5.53 and 4.69, and 176.7 and 72.8 mS, respectively. The effective magnetic moment values of compounds 1 and 2 were calculated and described in Text S1 and found to be similar to compound 4.^35^ The solid-phase UV-VIS spectroscopy (Fig. S1) studies of the compounds are described in Text S2 and agree well with Tsuchida^43,44^ and Yonade^45^ work.

### Structure of compounds 1 and 2

The crystalline and molecular parameters of compounds 1 and 2, obtained with SCXRD at 100 K, are summarized in Tables S1–S12. Compound 1 crystallizes in the orthorhombic crystal system and belongs to the *Pnma* space group, whereas compound 2 crystallizes in the monoclinic crystal system and belongs to the *P*2_1_/*c* space group (Table S1, Fig. 1, 2 and S2). Based on the cryo-DSC studies, none of the compounds exhibits a phase transition between −140 and 20 °C (Fig. S3). The measured PXRD agrees well with the one calculated from the SCXRD measurement (Fig. S4).

**Fig. 1:**
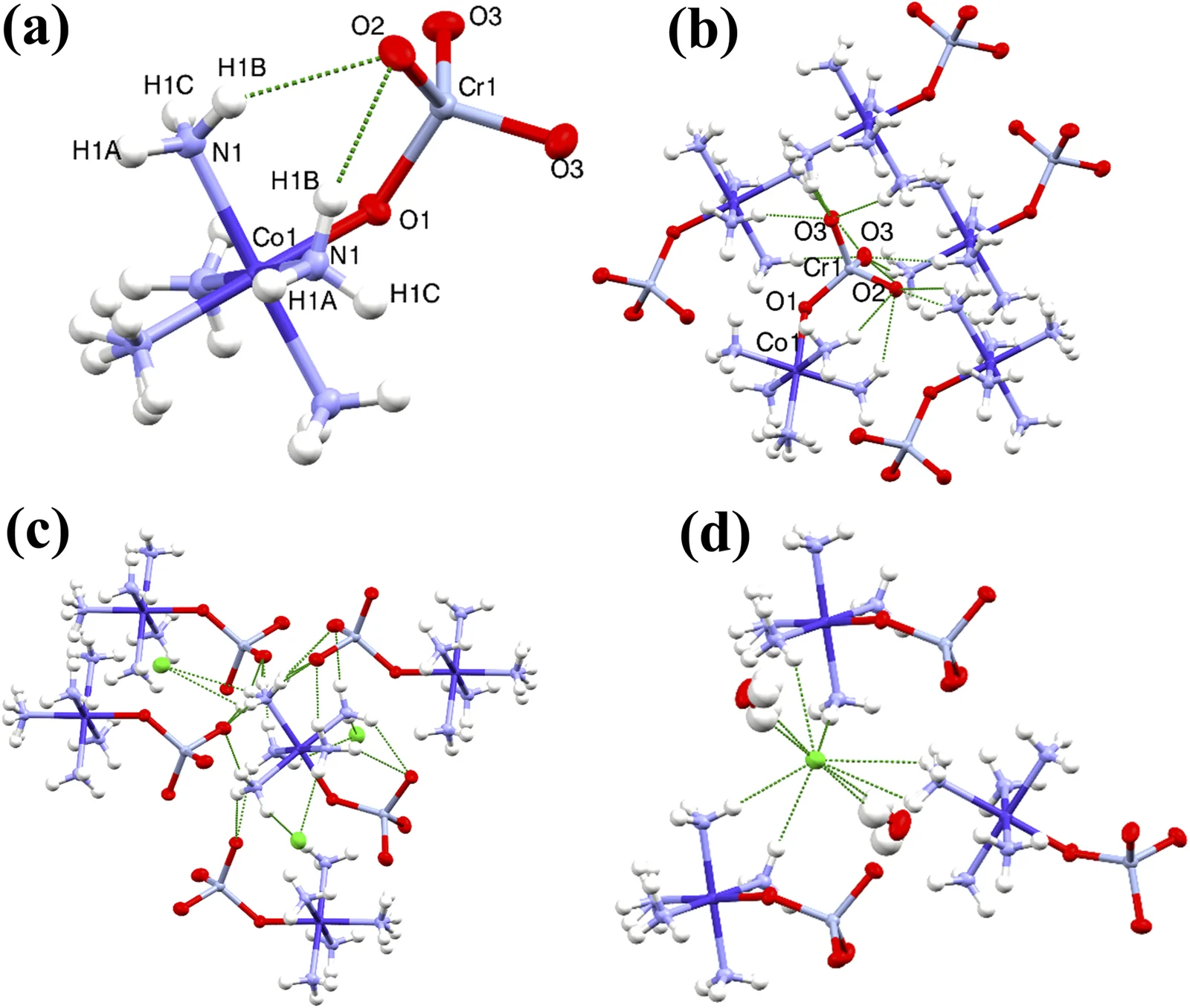
The intra- (a) and intermolecular (b) oxygen-hydrogen bonds, hydrogen bonds formed by NH_3_ ligands (c), and chlorine–hydrogen bonds (d) in compound 1.

**Fig. 2:**
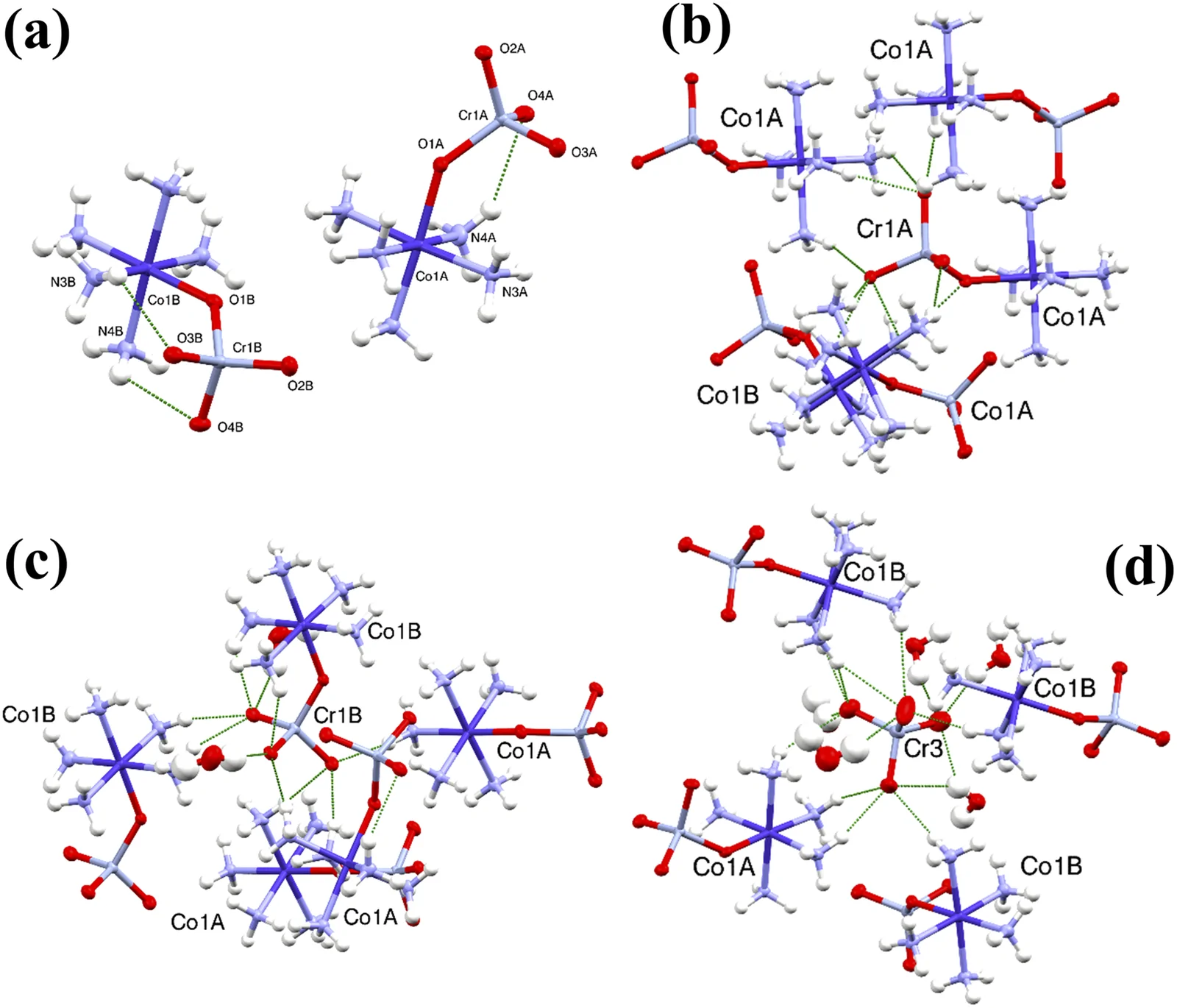
The intra- (a) and intermolecular (b) hydrogen bonds in compound 2. Hydrogen bonds formed by “Type A” CrO_4_^2−^ ligands (c) Hydrogen bonds formed by “Type B” CrO_4_^2−^ ligands. (d) Hydrogen bonds formed by CrO_4_^2−^ anions.

The asymmetric unit of compound 1 contains half of a [Co(NH_3_)_5_CrO_4_]^+^ cation, a Cl^−^ anion, and a water molecule (Fig. S2a), whereas that of compound 2 contains two [Co(NH_3_)_5_CrO_4_]^+^ cations, one CrO_4_^2−^ anion and four water molecules (Fig. S2(b)). In both cases, in addition to the five NH_3_ ligands, a monodentate chromate ligand, coordinated *via* one of its oxygens, is also found in the structure. The most common ligand form of the chromate ion is the bridging^46–49^ type; the monodentate form is quite unique,^50^ similar to the bidentate^51^ (chelating) form, since the chromate ion has multiple donor oxygen atoms.

In compound 1, the oxygen of the crystalline water (O4) and N2 (as well as Co1, O1, Cr1, and Cl1) are located on a mirror plane. The hydrogens attached to O4 (H4A, H4B) and N2 (H2A, H2B, H2B) are therefore symmetry-generated across the mirror plane and were modelled as disordered over two positions with equal occupancy (Fig. S2a). In compound 2, two crystallographically distinct complex cations are present; they differ in their coordination spheres and hydrogen-bonding scheme (Fig. 2).

The three (one in compound 1, and A and B in compound 2) CoN_5_O skeletons' geometry were found to be close to that of regular octahedron; the Stoeckli-Evans *τ*_6_ parameters^52^ showed only a weak distortion from octahedral geometry (*τ*_6_ = 0) to trigonal prism geometry (*τ*_6_ = 0.75), which were found to be 0.023, 0.038 and 0.024, denoted as 3.1, 5.1 and 3.3%, respectively. In the coordination sphere, the Co–N distances were similar in both compounds (Tables S3 and S6, for compounds 1 and 2, respectively) and varied between 1.948 and 1.967 Å, and 1.944 Å and 1.969 Å (compounds 1 and 2, respectively). The Co–O(–Cr) coordination distance in compound 1 was 1.917 Å, similarly to “Type A” [Co(NH_3_)_5_CrO_4_]^+^ cations in compound 2. However, in the case of “Type B” complex cation in compound 2, this distance is 1.899 Å (Tables S3 and S6). This difference can be explained by the different hydrogen bond network formed by the two types of cations. “Type B” [Co(NH_3_)_5_CrO_4_]^+^ cations in compound 2 form hydrogen bonds with 3 “Type A” and 1 “Type B” [Co(NH_3_)_5_CrO_4_]^+^ cations, and two water molecules (Fig. 2 and Table S8), whereas, “Type A” complex cations form hydrogen bonds with NH_3_ molecules of 4 adjacent [Co(NH_3_)_5_CrO_4_]^+^ cations (3 “Type A” and 1 “Type B”) (Fig. 2 and Table S8). In compound 1, the hydrogen bonding system is simpler: the CrO_4_^2−^ ion coordinated to the Co^3+^ ion forms two intramolecular hydrogen bonds with 2 coordinated NH_3_ molecules (the same is true for compound 2 (Fig. 1 and Table S5)), whereas it is connected to a total of four [Co(NH_3_)_5_CrO_4_]^+^ cations (to their NH_3_ ligands) through intermolecular hydrogen bonds (Fig. 1 and Table S5). In the case of counterions, in compound 1 each Cl^−^ ion interacts with 3 [Co(NH_3_)_5_CrO_4_]^+^ cations and two water molecules (Fig. 1(d)), whereas, in compound 2 the CrO_4_^2−^ forms hydrogen bonds with 4 [Co(NH_3_)_5_CrO_4_]^+^ cations (3 “Type A” and 1 “Type B”) and 5 water molecules (Fig. 1 and Table S5).

### Intermolecular interactions

To gain a deeper understanding of intermolecular interactions within the crystal structure, we performed a Hirshfeld surface analysis. The Hirshfeld surface partitions the volume of the crystal into two regions in which the sum of the electron densities of the selected component is larger or smaller than the contribution of the other atoms in the crystal, respectively. The distances of the nearest internal atom to the surface (*d*_i_), the nearest external atom to the surface (*d*_e_), and the sum of the two values normalized by the corresponding van der Waals radii (*d*_norm_) are used to analyse interactions within the crystal structures (Fig. 3).

**Fig. 3:**
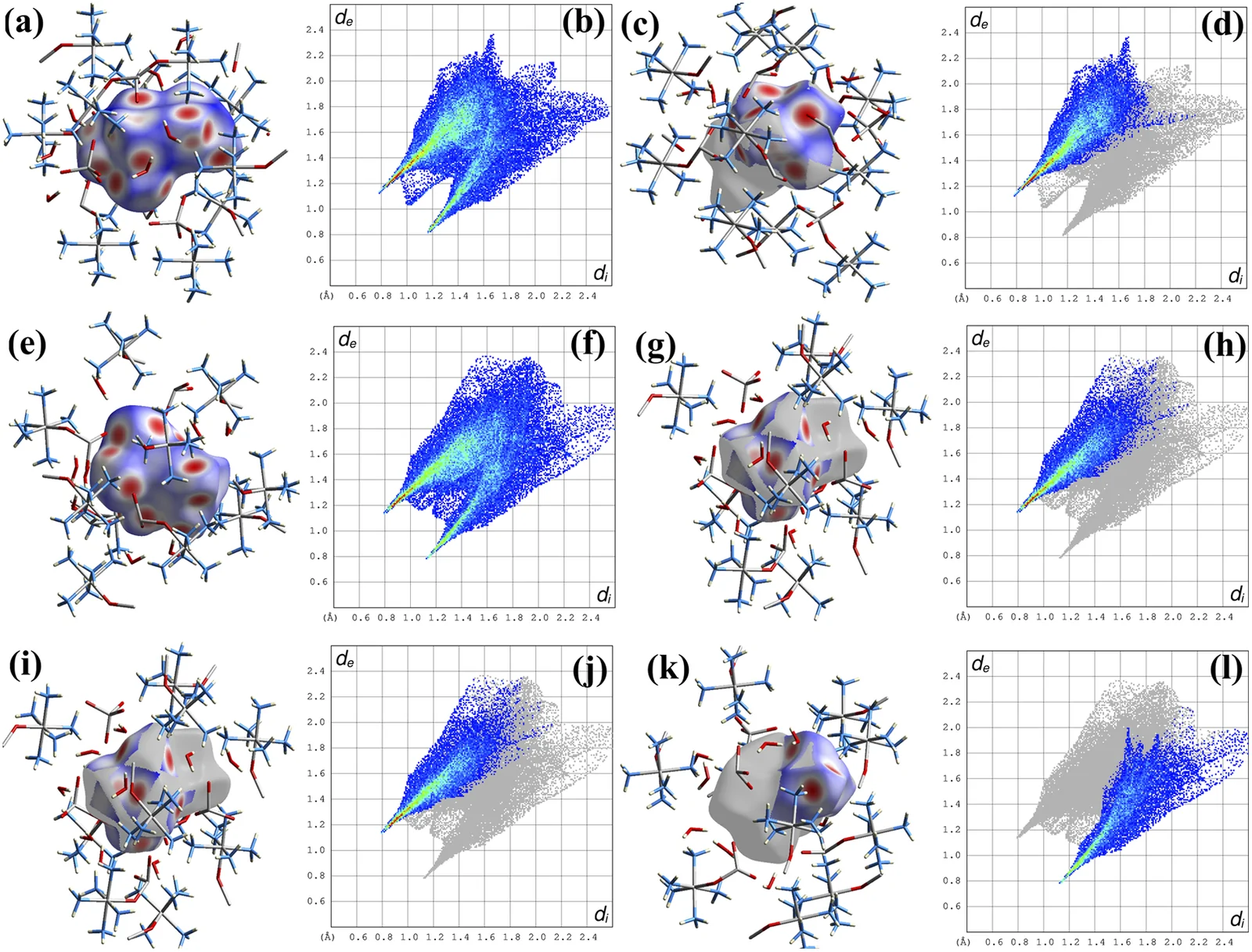
Hirshfeld surface analysis of “Type A” (a–f) and “Type B” (g–l) [Co(NH_3_)_5_CrO_4_]^+^ cations. Hirshfeld surfaces and corresponding fingerprint plots showing all interactions of “Type A″ cations (a and b) and “Type B″ cations (g and h); Hirshfeld surfaces and fingerprint plots showing N–H⋯O interactions (with N–H being inside the surface) for “Type A” (c and d) and “Type B” (i and j) cations and Hirshfeld surfaces and fingerprint plots showing O⋯H–N and O⋯H–O interactions for “Type A” (e and f) and “Type B” (k and l) cations. The regions on the Hirshfeld surface, where the sum of *d*_i_ and *d*_e_ is smaller (strong interaction), equal to, or larger (weak interaction) than the sum of the van der Waals radii of the corresponding atoms, are marked with red, white, and blue colours, respectively. Contacting molecules are shown as sticks.

The Hirshfeld surface analysis was done only on compound 2 to show the non-covalent interactions (Fig. 3), because the disordered ammonia and water molecules in compound 1 could not be evaluated by the Crystalexplorer software.^53^

The hydrogen bond interactions can be readily identified in the fingerprint plots as two distinct spikes corresponding to the O⋯H–N/O⋯H–O and N–H⋯O interactions. The number of N–H⋯O interactions (Fig. 3) is larger than the number of O⋯H–N and O⋯H–O interactions, as indicated by the red values on the Fingerprint plots. Differences can be observed between the fingerprint plots of the two types of cations in both regions of the plot, indicating that their hydrogen bonding networks are indeed different. The surfaces also show that the interactions of the cation are asymmetric, as chemically identical NH_3_ or CO groups form interactions that differ in both strength and the number and identity of their interacting partners.

The packing of compound 1 shows a column along the *a* axis that contains Cl^−^ and water molecules (Fig. S2(c)). Compound 2 also contains a layered structure along the *b* axis in a way that one layer containing only “Type A” [Co(NH_3_)_5_CrO_4_]^+^ cations is followed by two layers containing “Type B” [Co(NH_3_)_5_CrO_4_]^+^ cations, water molecules, and CrO_4_^2−^ ions (Fig. S2(d)). These columns and layers are stabilized by numerous hydrogen bonds discussed previously.

We have found^4,10,11,13^ that the number and positions of the hydrogen bonds have an important influence on the probability and the kick-off temperatures of the quasi-intramolecular redox reaction (in the form of reverse correlation, *i.e.*, the higher the “hydrogen bond density”, the lower is the decomposition temperature). The hydrogen bond density (number of hydrogen bonds/Å^3^) was found to be 49.6 and 71.3 in compounds 1 and 2, respectively. In the case of the previously published [Co(NH_3_)_5_Cl](MnO_4_)_2_,^13^ which was proven to be a good precursor material to prepare Co–Mn mixed oxides at low temperature (below 200 °C) by SPQIR, this value was found to be 54.5,^13^ both complexes are potential precursor materials to prepare cobalt-chromium oxides.

### Optimization of structure and stabilization interactions

The structural optimization was performed to determine starting geometries for the stability analysis, and the experimental structures were optimized at B3LYP/6-311++G(d,p)(H,N,O,Cl)/LanL2DZ(Co, Cr) level of theory without any geometrical constraints (Tables S9–S12). For complexes 1–3, the neutral forms containing all cations and anions were selected. The comparison of experimental and theoretical bond lengths and angles included calculation of the correlation coefficient and the mean absolute error (MAE) between the two data sets. The latter parameter gives the average absolute difference between experimental and theoretical structural parameters.

The experimental and theoretical bond lengths and angles are presented in the SI. The correlation coefficients for the comparison of bond lengths in the cases of compound 1 and compound 2 are 0.98 and 0.99, respectively. When bond angles are compared, the correlation coefficients are 0.99. The MAE values for bond lengths are 0.04 (compound 1) and 0.03 (compound 2), while the MAE values for bond angles are around 4°. This result is expected, since the structural optimization was performed for an isolated compound in vacuum, whereas the crystallographic structure includes various intermolecular interactions, as previously explained. It was particularly challenging to compare the bond lengths and angles within the coordination sphere of the central metal ion, as well as the chromate ion, due to the local symmetry of these groups. Nevertheless, the observed differences are within the expected range for this level of theory and demonstrate that the structures can be used to further analyse intramolecular stabilization interactions.

The QTAIM analysis was performed on the optimized structures, as explained for similar compounds.^54–56^ The selected Bond Critical Points (BCPs) were examined by calculating electron density (*ρ*(r)), Laplacian of the electron density (∇^2^*ρ*(r)), Lagrangian kinetic energy density (*G*(r)), potential energy density (*V*(r)), total electron energy density (*H*(r) = *G*(r) + *V*(r)), and the interatomic bond energy (*E*_bond_ = *V*(r)/2). Based on the electron density, the interactions can be classified as shared (covalent bonds) with *ρ*(r) > 0.1 a.u. and closed-shell (ionic bonds, hydrogen bonds, and van der Waals forces) with *ρ*(r) of around 0.01 a.u.^57,58^ Bianchi and coworkers presented a more detailed classification that examines the ratio of kinetic (*G*(r)) and potential (*V*(r)) energy.^59^ When the −*G*(r)/*V*(r) value is below 1, the interaction is covalent; intermediate (transit) interactions are between 1 and 2; and interactions with values above 2 are ionic. Additionally, negative total energy (*H*(r)) also represents covalent interactions. The interatomic interaction energy can be determined as half the values of the potential energy density, as proposed in the paper by Espinosa.^60,61^ The QTAIM parameters of the selected interactions are presented in Table S13, with BCPs depicted in Fig. S5. It should be mentioned that only one of the Co–N is presented for illustrative purposes, and slight changes in QTAIM parameters are possible due to the additional interactions with surrounding ions.

In the structure of compound 1, the highest values of electron density (0.265 a.u.) and Laplacian (1.115 a.u.) were obtained for the Cr–O bond. This bond is characterized by the most negative total electron energy and the interatomic interaction energy of −767.9 kJ mol^−1^. These values demonstrate the stability of the chromate ion, as expected from its structural features. Once the oxygen atom of the chromate ion interacts with Co, the Cr–O bond energy is reduced to −378.2 kJ mol^−1^, with lower values of electron density and the Laplacian. In the complexation sphere around the cobalt ion, a stronger interaction is found between the central metal ion and the oxygen atom (−218.6 kJ mol^−1^) compared to the Co–N bond (−178.5 kJ mol^−1^). All these interactions show typical properties of covalent interactions. In the optimized structure of compound 1, the interaction between ammonia hydrogen atoms and chloride anions is also strong with partial covalent character due to the difference in charge and proximity of these two groups.

The structure of compound 2 is characterized by the presence of the chromate ion in the coordination sphere and in the outer sphere. The interaction properties of Cr–O are similar in chromate ions coordinated and non-coordinated to the cobalt ion. The electron densities are around 0.240 a.u. with Laplacians of around 1.00 a.u. The ratio −*G*(r)/*V*(r) is the lowest (0.7) compared to other interactions in this compound. The interatomic interaction energies are around −670 kJ mol^−1^. This value is lower than that of compound 1 due to the presence of various weak interactions between the constituents. Again, much lower energy was found for the Cr–O bond, which includes an oxygen atom coordinated to cobalt (−394.4 kJ mol^−1^). In compound 2, the interaction between Co and oxygen is weaker than in compound 1, again due to interactions with surrounding units. Other interactions in the coordination sphere have slightly lower interaction energy (184.6 kJ mol^−1^). The optimized structure of compound 2 is further stabilized by the N–H⋯O interactions that shows partial covalent character with energies of −80.0 kJ mol^−1^, which are significantly higher than expected.

Once the chlorido ligands are present in the coordination sphere ([Co(NH_3_)_5_Cl]Cl_2_), the characteristics of Co–N interactions are similar to those in compound 1. It can be concluded that the interactions between the central metal ion and chlorido ligands are the weakest of all examined in this part, with energies of −78.6 kJ mol^−1^. This result is a consequence of the donation properties of the chlorido ligand. Interaction denoted as Co–Cl has a partial covalent character, as represented by the negative value of *H*(r) (−17.3 kJ mol^−1^) and −*G*(r)/*V*(r) value of 0.9. The interactions formed between amino hydrogen atoms and chloride anions are of the same strength as those in compound 1.

### Vibrational spectroscopic studies of compounds 1 and 2

The IR spectrum of compound 1 was recorded by Duval and Lecomte,^62^ without assignments of band positions, and later Coomber and Griffith^14^ made room-temperature IR and Raman measurements to study the effect of coordination on the shift of chromate-ion *ν*_1_ (*ν*_s_, symmetric stretching mode) band positions, including deuteration to separate the overlapping N–H and Cr–O bands. Coomber and Griffith^14^ found a doublet *ν*_3_(*ν*_as_) band, as was expected for a distorted monodentate-coordinated (*C*_3v_) chromate ion. No vibrational analysis has been done for compound 2 yet. The experimental vibrational spectroscopic studies on compounds 1 and 2 were performed at room temperature (IR, far-IR, and Raman), but the Raman spectra were also recorded at liquid N_2_ temperature with 785 nm and 532 nm laser excitations (Fig. 4 and S6–S11). Our X-ray studies showed that the chromate ions in compounds 1 and 2 exhibit *C*_s_ and *C*_1_ local-group symmetry, respectively, due to the presence of extended hydrogen-bond systems. Coomber and Griffith's interpretation^14^ about monodentate chromate-ion coordination in compound 1 was correct, however, the chromate ion symmetry is lower than *C*_3v_ due to the solid-phase interactions within the crystals (see crystallographic results), and the doublet peaks in the IR and Raman spectra were observed only due to the overlapping of the expected three (F_2_) *ν*_3_ (*ν*_as_) band components. Therefore, knowing the structural parameters, we have done a detailed correlation analysis for both compounds, and in addition to the mentioned IR, far-IR, and low-temperature Raman (125 K) studies, the IR spectra of the deuterated compounds 1-D and 2-D were also recorded.

**Fig. 4:**
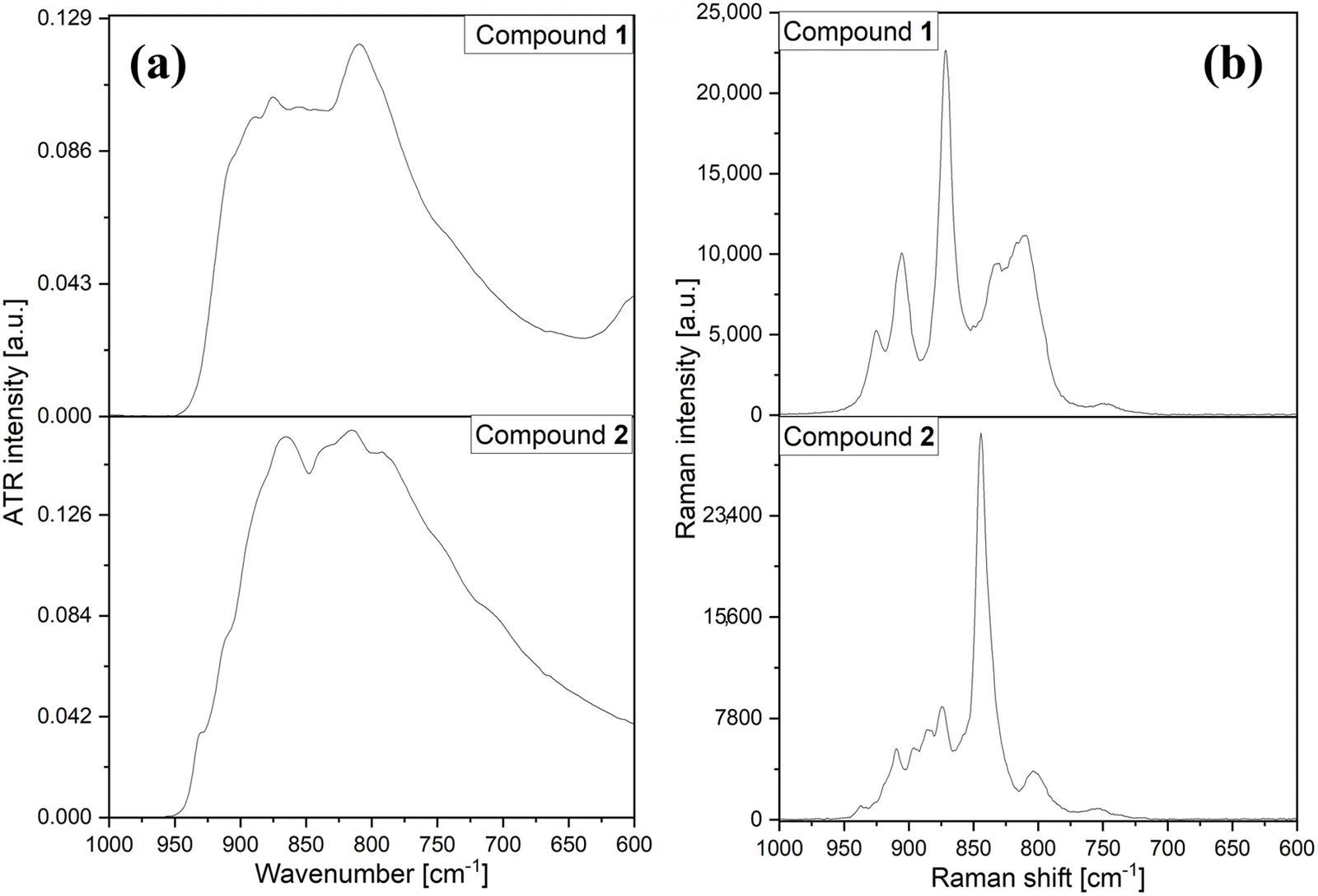
The analytic range IR (*T* = 297 K) (a) and Raman (*T* = 123 K, 784 nm excitation) (b) spectra of compounds 1 and 2 between 1000 and 600 cm^−1^.

### The vibrational modes of chromate ions in compounds 1 and 2

The coordinated chromate anions in compounds 1 and 2, and the non-coordinated one in compound 2 are tetrahedral, accordingly to a first approximation, four normal vibrational modes (*ν*_1_–*ν*_4_) are expected for all of them, two stretching and two deformation modes (symmetric and antisymmetric), *ν*_s_(*ν*_1_), *ν*_as_(*ν*_2_), *δ*_s_(*ν*_3_) and *δ*_as_(*ν*_4_), respectively. The degenerate modes are split due to site-symmetry effects (decrease of the tetrahedral symmetry), and further splitting is possible as a result of interactions among identical oscillators (*e.g.*, Davydov splitting^63^).

The correlation analysis results for compounds 1 and 2 were given according to the ‘fragmentation’ of these compounds into Co^3+^ and Cl^−^ (Fig. S12), or CrO_4_^2−^ ions (Fig. 5 and S13), and NH_3_ (Fig. S14–S17) or H_2_O (Fig. S18 and S19) molecules. The *T*_d_ symmetry of the isolated chromate anions in compounds 1 and 2 changed into *C*_s_ and *C*_1_ due to the coordination to cobalt and/or the hydrogen bond interactions with *D*_2h_ and *C*_2h_ unit-cell (‘factor groups’, respectively).

**Fig. 5:**
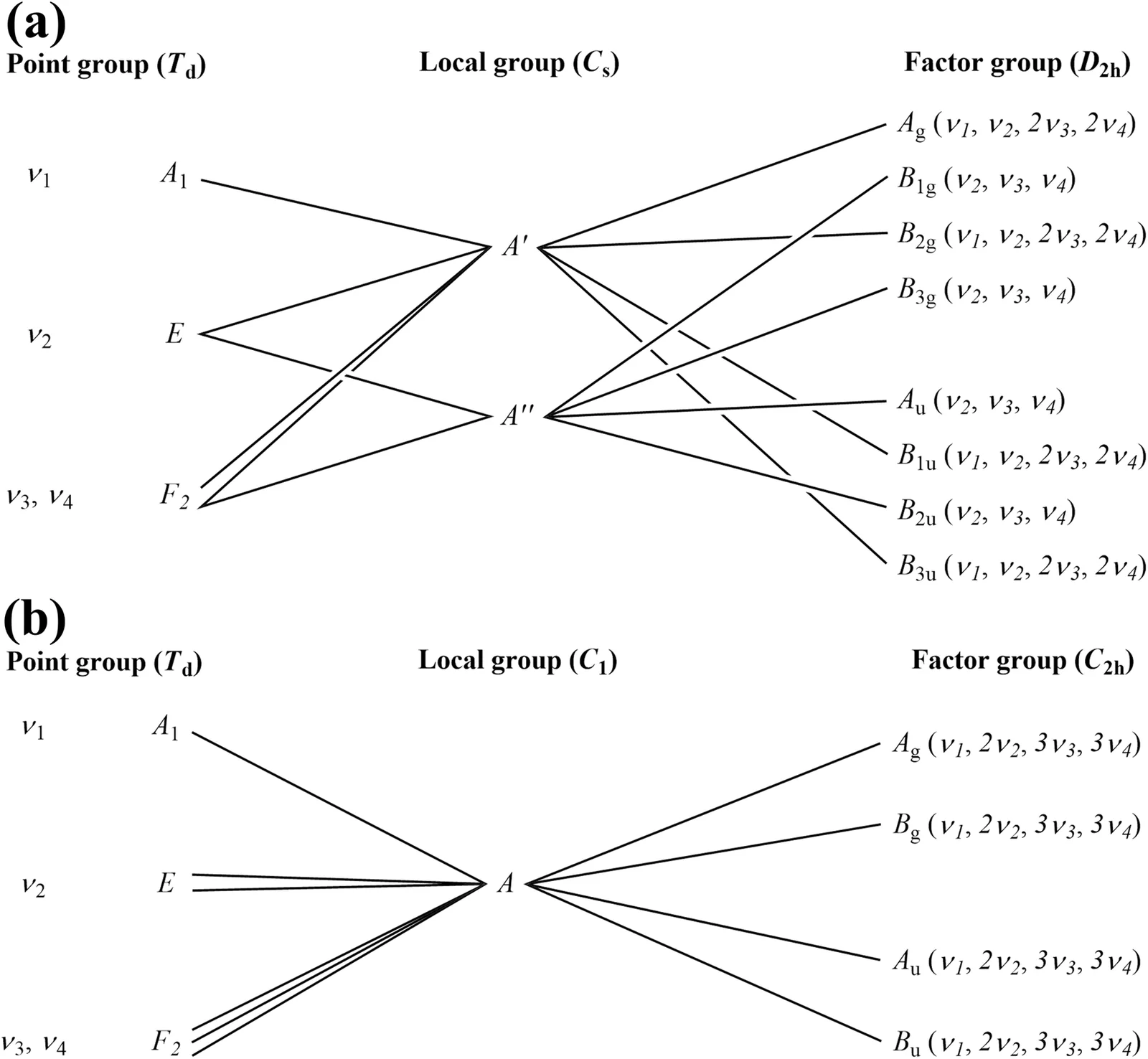
The internal vibrational modes of chromate ion in compounds 1 (a) and 2 (b).

The correlation analysis showed that all vibrational modes are IR- and Raman-active, and that two sets of vibrational degrees of freedom are observed for both compounds. We can expect 2 × 2 and 2 × 3 series of a *ν*_1_ (A) singlet, *ν*_2_ (E) doublets, and *ν*_3_ (F_2_) and *ν*_4_ (F_2_) triplets, both in the Raman and IR spectra of compounds 1 and 2, respectively. There are 18 IR and 18 Raman active external modes, which are duplicated and triplicated due to the presence of two and three crystallographically different chromate ions in the structures of compounds 1 and 2 (Fig. 5 and S13), respectively.

To distinguish the coinciding Cr–O and N–H bands in the spectra of compounds 1 and 2 (Fig. 4 and S6–S11), the IR and low-temperature Raman spectra of compounds 1 and 2 (Fig. 4), and the IR spectra of deuterated 1-D and 2-D (Fig. S20), were evaluated in detail. These studies are essential to distinguish the coordinated and non-coordinated chromate peaks, and to identify the normal modes of ammonia and that of lattice water.^64^

The assignments of Coomber and Griffith^14^ for Cr–O modes in compound 1 were correct; the band assignments of chromate ion in compounds 1 and 2 are given in Table S14. The coincidence of *ν*_1_(Cr–O) and *ν*_3_(Cr–O) bands with *ρ*(NH_3_) bands, in the IR spectra of compounds 2 and 2-D (Fig. S20), and in the LT Raman spectrum (Fig. 4) of compound 2 in the 600–1000 cm^−1^ wavenumber range were analysed in detail. The new *ρ*(ND_3_) band of the [Co(ND_3_)_5_CrO_4_]^+^ core of compound 2-D was assigned at 665 cm^−1^ in the IR spectra with the use of the *φ* = *ρ*(NH_3_)/*ρ*(ND_3_) peak position ratio calculated for compound 1 (*φ* = 1.24).^14^ Accordingly, since compound 1 has the same [Co(NH_3_)_5_CrO_4_]^+^ core as compound 2, the position of the original *ρ*(NH_3_) in compound 2 was identified at 815 cm^−1^, which disappeared after deuteration (Fig. 4 and S20). The measured *ρ*(ND_3_) for compound 1 was found to be 666 cm^−1^; thus, the calculated *ρ*(NH_3_) for the non-deuterated compound 1 was 813 cm^−1^.

The symmetric stretching mode (*ν*_1_) is a weak band in the IR spectrum of compound 2, but it is the most intense band in the Raman spectra of tetrahedral anions.^64^ Accordingly, the two most intense Raman bands at 845 cm^−1^ and 874 cm^−1^ could be assigned as the *ν*_1_ band of the outer and inner-sphere, non-coordinated and coordinated chromate ions, respectively (Table S14).

Two series of triplet *ν*_3_ bands could be observed, one belonging to the coordinated and another to the non-coordinated chromate ions. The wavenumbers of *ν*_1_ and *ν*_3_ modes of the coordinated Cr–O bonds have increased as compared to the non-coordinated ones due to the formation of the Co–O–Cr bond, which increased the degree of multiplicity in the remaining Cr–O bonds (formal Cr=O) of CrO_4_^−^ units. The deuteration caused some shift of *ν*_1_ and *ν*_3_ bands to lower positions due to the small change in the strength of CrO_4_⋯H(D)–N/H(D)–O hydrogen bonds upon deuteration. The LT bands in the Raman spectrum are better resolved than those in the room-temperature IR spectra and show two series of low-intensity triplet Raman bands, belonging to the coordinated and non-coordinated chromate ions. To identify the coordinated and non-coordinated *ν*_3_(Cr–O) band positions, we compared the IR data given for compound 1 ([Co(NH_3_)_5_CrO_4_]Cl, containing only inner sphere chromate) and [Co(NH_3_)_5_Cl]CrO_4,_ containing only outer sphere chromate ion).^14^ The *ν*_2_ and *ν*_4_ deformation modes of chromate ion in compounds 1 and 2 are located in the far-IR region. The LT-Raman spectra of compounds 1 and 2 show bands belonging to these modes at 344/326 and 326/314 cm^−1^, as well as at 380/370/363 cm^−1^ and 386/380 cm^−1^ with a shoulder, respectively, where the high and low wavenumber range components of the band pairs in the spectrum of compound 2 belong to the non-coordinated and the coordinated chromate species,^14^ respectively. The multiple (doublet and triplet) nature of *ν*_2_, *ν*_3_, and *ν*_4_ bands might be observed only in LT-Raman spectra of compounds 1 or 2; the room temperature IR spectra contain overlapped bands. The vibrational modes of [CoN_5_O] skeleton and Co–O–Cr linkage in compounds 1 and 2 discussed in detailed manner in Text S3, while the correlation analysis of water molecules in Text S4.

### The ammonia ligand vibrational modes of compounds 1 and 2

The correlation analysis of ideal *C*_3v_ ammonia ligands in compound 1 (Fig. S14–S17) showed that the local group for ammonia molecules is *C*_s_ in the axial (*trans* to chromate oxygen) and *C*_1_ in the equatorial position. The *ν*_3_, *ν*_4_ modes are doubly degenerate under *C*_3v_, but their components under *D*_2h_ are nondegenerate. Due to the two crystallographically different ammonia ligands (*C*_1_ and *C*_s_), 8*ν*_1_, 8*ν*_2_, 16*ν*_3_ or 16*ν*_4_ and 4*ν*_1_, 4*ν*_2_, 8*ν*_3_ or 8*ν*_4_ vibrational degrees of freedom for ligands may be expected, together with 2 × 3 hindered translations and 2 × 3 hindered rotations for ammonia molecules (Fig. S14 and S15), respectively, both in the IR and Raman spectra of compound 1.

For compound 2, the 2*ν*_1_, 2*ν*_2_, 4*ν*_3,_ and 4*ν*_4_ vibrational degrees of freedom were expected ten times, because of the presence of 2 × 5 = 10 various ammonia (*C*_3v_ point group, *C*_1_ local group, *C*_2h_ factor group) ligands. It denotes two distinct CoN_5_O skeletons, each with 5 distinct ammonia ligands.

The assignment of ammonia internal vibrational modes for compounds 1 and 1-D agrees very well with the results given by Coober and Griffith.^14^ The assignments of ligand vibrations for compounds 1, 1-D, 2, and 2-D are given in Table S15.

The *ν*(N–H) stretching modes showed lower wavenumber values for compound 2 than for compound 1 due to the much higher hydrogen bond density in the unit cell (49.6 and 71.3 pieces/Å^3^ for compounds 1 and 2, respectively); see the crystallographic part. The splitting of *ν*(N–H) and *δ*(N–H) bands might be attributed to the different structural positions of the apical and equatorial ligands relative to the oxygen. The overlapping *δ*_as_(H_2_O) (scissoring) mode of crystalline water and *δ*_as_(NH_3_) mode bands gave a partially resolved band system after deuteration, and the calculated *φ* value for one of the peaks (*δ*_as_(H_2_O), 1191 cm^−1^) was used for estimation of the coinciding positions of the *δ*_as_(N–H) bands in compounds 1 and 2 before deuteration, and found the values of 1541 cm^−1^ and 1562 cm^−1^, respectively. The Co–N bond lengths were also varied between 1.948-1.967 Å and 1.944–1.967 Å in compounds 1 and 2, respectively (Tables S3 and S6), resulting in differences in the relative bond strength. The relative bond strength parameter for ammonia complexes (*ε*) defined by Grinberg^65^ and determined from *δ*_s_ values, was found to be between 0.81 and 0.94 as well as between 0.80 and 0.98 for compounds 1 and 2,^3,6,65^ respectively. The large differences in the *ε* values for the ammonia ligands in compounds 1 and 2 indicated that their expected reactivity toward SPQIR might be very different. The relative strength of ammonia ligands in the complexes 1 and 2 is partly comparable with the values found for [Co(NH_3_)_5_Cl](MnO_4_)_2_ (0.88 and 0.80)^13^ (precursor of CoMn_2_O_4_ spinel catalysts) and proved to be one of the strongest ones (*ε* = 1 for the [Pt(NH_3_)_4_]^2+^ cation^65^). The hydrogen bonds formed by ammonia ligands in compounds 1 and 2 are not equal; the hydrogen bond density across the various ammonia molecules in these interactions is not equal.

### The TG-MS studies on compounds 1 and 2

The TG-MS and DSC measurements on compounds 1 and 2 were performed under both inert (He) and oxidative (air) atmospheres. Low-temperature DSC studies were also performed (Fig. S3), which showed the absence of any low-temperature phase transformation of compounds 1 and 2. The thermal decomposition processes start with the loss of their crystal water content almost completely for compound 1 and only partly for compound 2 (∼5 and ∼6%, Tables S16 and S17, respectively), even below 150 °C. The main decomposition steps consist of simultaneous redox and ligand elimination processes, with ∼215 and ∼230 °C peak temperatures for compounds 1 and 2, respectively, both in an inert and oxidative atmosphere. This shows that oxygen has no role in the initiation of the decomposition processes; thus, these are SPQIRs, as was expected.

Water elimination is followed by a simultaneous loss of additional water, ammonia, and ammonia oxidation products such as N_2_ (*m*/*z* = 28) and N-oxides (NO, N_2_O), both in inert and oxidative atmospheres (Fig. 6). The appearance of the ammonia oxidation products (NO and N_2_O) in the inert atmosphere (Fig. 7) unambiguously confirmed the appearance of SPQIR. The reduction product of chromate and cobalt(iii) ions is expected to be Cr^III^ and Co^II^; thus, the chromate ion/Co^III^(NH_3_)_5_ unit ratio of 1 and 1.5 in compounds 1 and 2, respectively, showed that the chromate ion (or cobalt(iii)) can oxidize only a fraction of the ammonia ligands. The decomposition reactions of compounds 1 and 2 started as endothermic and exothermic processes, respectively, but the overall decomposition of compound 2 becomes endothermic (Fig. 7).

**Fig. 6:**
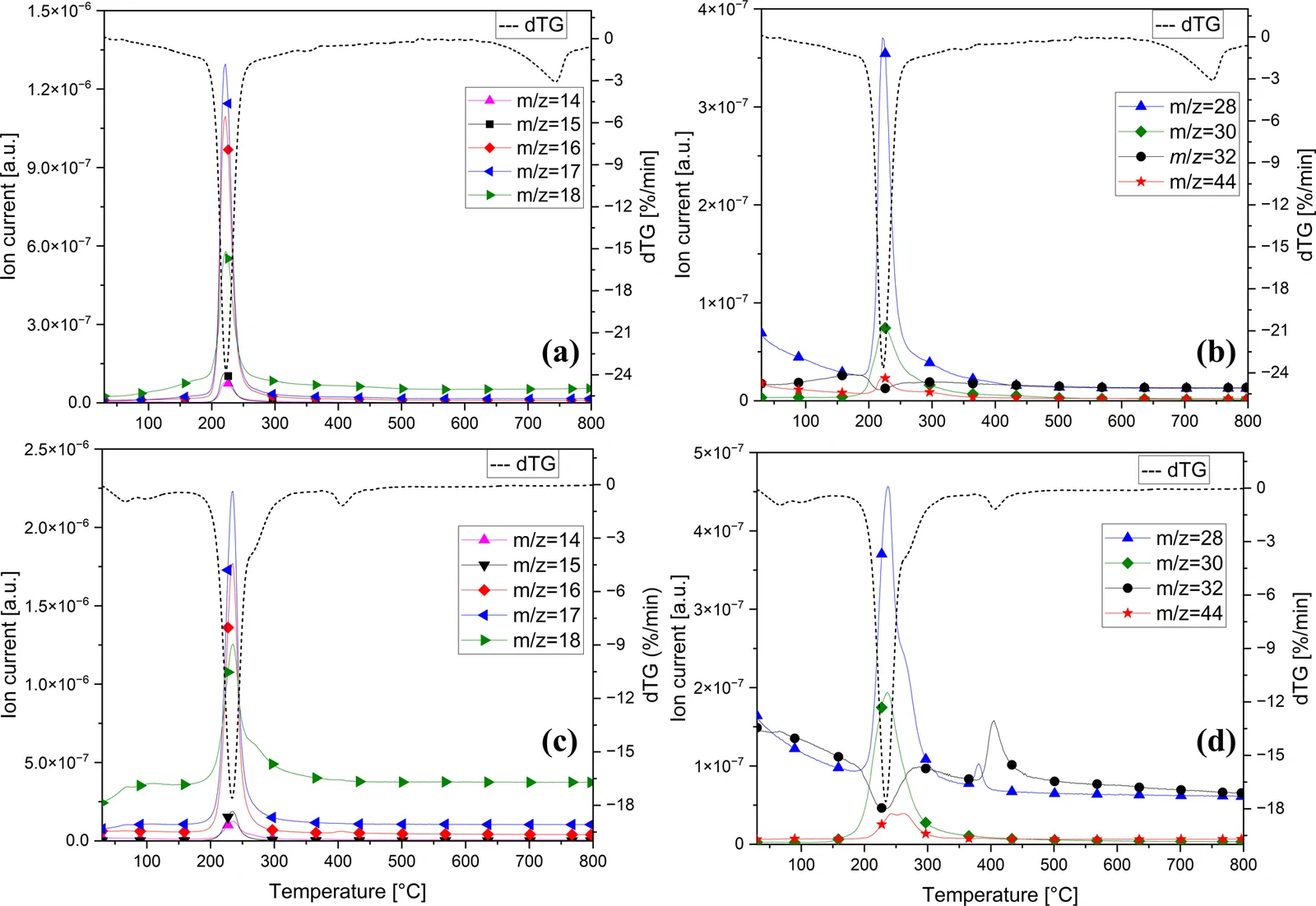
The MS ion curves of compounds 1 (a and b) and 2 (c and d), measured with TG-MS in inert atmosphere.

**Fig. 7:**
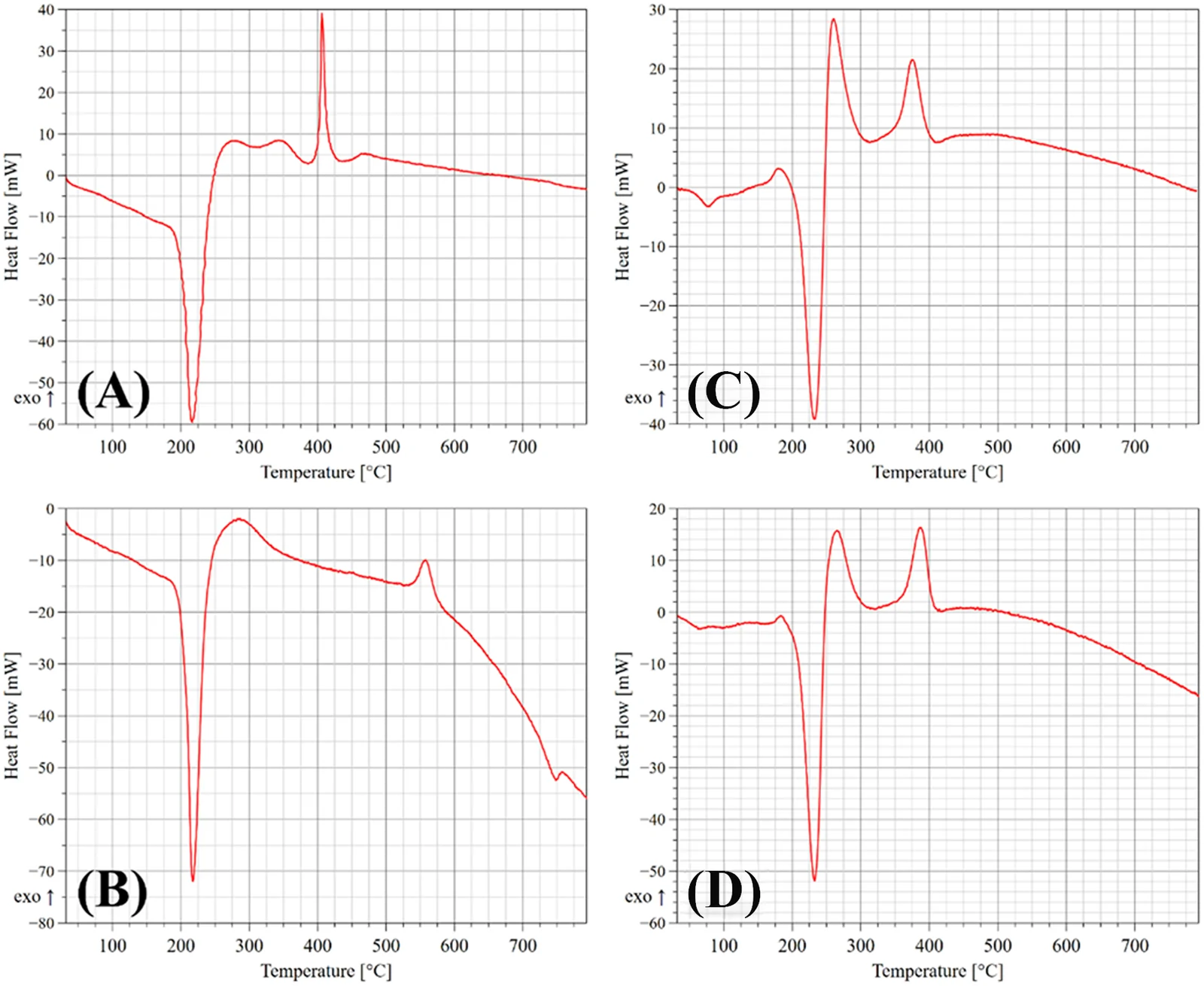
The DSC curves of compound 1 (left) in (A) oxidative and in (B) inert atmosphere, and of compound 2 (right) in (C) oxidative and in (D) inert atmosphere.

The decomposition of compound 1 in both atmospheres starts with the endothermic process of ammonia ligand loss of the anhydrous compound 1, followed by the inner sphere coordinated chromate ion mediated oxidation of some of the residual coordinated ammonia ligands.

The most likely intermediate is expected to be the relatively stable bidentate chromate-coordinated [tetraammine(κ^2^-chromato)cobalt(iii)] cation.^36^ This intermediate may decompose in an SPQIR, with partial oxidation of its ammonia content and liberation of excess ammonia. The evolved heat of the redox reaction between chromate/Co^III^ and ammonia cannot compensate for the endothermic heat required to drive residual ammonia ligand loss, so the overall process is endothermic. In the decomposition process of compound 2, however, the decomposition starts with exothermic character, which led us to suppose that the outer-sphere chromate ion initiates the oxidation of the 5th ammonia ligand; thus, the first moment of the multistage decomposition is also an ammonia loss, but not with an endothermic ligand loss, but *via* exothermic oxidation. It means that the reactivity of the outer-sphere chromate ion is probably significantly higher than that of the coordinated one in compound 2. The reaction heat of the SPIQR in the first decomposition stage of compound 2 by the outer-sphere chromate ion can overcompensate for the heat demand of the residual crystal water loss (Table S17 and Fig. 6), and the supposed [tetraammine(κ^2^-chromato)cobalt(iii)]-containing intermediate decomposes further *via* SPQIR with similar attributes as in the decomposition of compound 1 (Fig. 6 and 7). The TG weight losses (Table S16) in inert atmosphere and in air for compound 1 (50.4% and 57.2%) and for compound 2, (43.9% and 43.3%), respectively (Fig. S21 and S22), showed that air played a role in the decomposition of compound 1.

On increasing temperature, two exothermic processes appeared at 349 and 406 °C, with peak reaction heats of −21.17 and −38.45 kJ mol^−1^, respectively, under a He atmosphere, but not in an air atmosphere. This suggests that the intermediates decompose exothermally and can form only under reductive conditions. In the presence of air, a new peak appeared at 748 °C peak temperature with −16.26 kJ mol^−1^ reaction heat, which may be attributed to the oxidation of Co^II^ content into Co^III^ within the Co-rich spinel lattice^66^ (Fig. 7). This reaction is highly significant for the preparation and tuning of Co^III^-containing potential catalysts.

The weight loss in the main reaction is higher than expected from the evolution of ammonia, due to the loss of oxygen in the form of evolved N-oxides and water, and to the chloride content in the form of HCl (NH_4_Cl). We have not found signals of chlorine elimination as HCl due to the reaction of ammonia and Cl_2_, at *m*/*z* = 36 and 38 (HCl^+^), because the ammonia reacts with HCl and NH_4_Cl was deposited as was found in the case of [Co(NH_3_)_5_Cl](MnO_4_)_2_ (ref. 13) and [Co(NH_3_)_6_]Cl_2_MnO_4_.^11^ The comparison of the experimental weight of the final decomposition residue and the calculated value for the formation of a Co–Cr spinel with Co to Cr stoichiometry 1 : 1, formally Co[Co_0.5_Cr_1.5_O_3.75_] (Table S16), showed the complete elimination of the chloride ion.

The main decomposition step of compound 1 had a maximum around 215 °C in both inert and oxidative atmospheres, with close *ΔH*_r_ values (418.4 and 394.9 J g^−1^, respectively) (Fig. 7). The formation of N_2_ (*m*/*z* = 28) and NO = *m*/*z* = 30) was observed in this step, together with N_2_O and H_2_O formation (Fig. 6), which is clear evidence of SPQIR.^10,11,13^ The formation of H_2_O and N_2_O with coinciding peak positions is a typical sign of the presence of NH_4_NO_3_ intermediate (NH_4_NO_3_=N_2_O + H_2_O) in SPQIR.^67^

From the intensity ratio of *m*/*z* = 17 (NH_3_^+^ and HO^+^) and *m*/*z* = 18 (H_2_O^+^) fragments, compared to the intensity of a pure ammonia fragment *m*/*z* = 15 (NH^+^) (the fragment with *m*/*z* = 16 may be NH_2_^+^ and O^+^; *m*/*z* = 14 (N^+^) fragment may be liberated not only from NH_3_ but from NO, N_2_ and N_2_O as well), a simultaneous formation of ammonia and water could be concluded (Fig. 6). Thus, the oxidation of ammonia to NH_4_NO_3_ and its subsequent decomposition into N_2_O and H_2_O occur in a single step.

The thermal decomposition of compound 2 results in an oxygen-deficient cobalt-rich spinel, Co[Co_0.2_Cr_1.8_O_3.90_], with a main decomposition peak at 230 °C. The overall weight losses in the oxidative and inert atmospheres were 33% and 34% (instead of 24% of the NH_3_ content), respectively, for compound 2 (Table S17 and Fig. S22), providing evidence of a redox reaction between the CrO_4_^2−^ and the ammonia ligands. The presence of higher chromate/Co^III^-complex cation ratio than in compound 1 resulted in the formation of more chromate-oxidized intermediate, which decomposes in exothermic processes with 260 and 374 °C as well as with 265 and 388 °C peak temperatures with −147.39 J mol^−1^ and −109.04 kJ mol^−1^ as well as with −120.81 kJ mol^−1^ and −114.17 kJ mol^−1^ reaction heats, in He and air atmospheres, respectively. The character of the decomposition is almost the same in both atmospheres. The thermal decomposition process of compound 2 starts with water loss below 100 °C, with 23.18 and 20.75 kJ mol^−1^ heat absorption, and the main reaction starts as an exothermic process with −12.29 kJ mol^−1^ and −6.74 kJ mol^−1^ heat releases, followed immediately by endothermic ones, with 254.90 kJ mol^−1^ and 281.74 kJ mol^−1^ heat absorptions at 180 °C and 184 °C, as well as at 233 °C and 232 °C peak maxima, in He and air atmospheres, respectively (Fig. 7).

The TG-MS analysis of gaseous decomposition products shows first the elimination of a part of the crystal water, then the formation of ammonia and water together (from the intensity ratios of *m*/*z* = 17 and 18, OH^+^/NH_3_^+^ and H_2_O^+^ peaks, and *m*/*z* = 15, NH^+^), without oxygen (*m*/*z* = 32) evolution. As ammonia oxidation products, NO and N_2_O could also be detected, and there are two separate N_2_O formation processes, probably due to the presence of catalytic (catalysed by the intermediate Co–Cr oxides) and non-catalytic ammonium nitrate decomposition; there are reactions centred around 240 and 260 °C, respectively (Fig. 6).

### The preparation and characterization of Co–Cr spinel oxide catalyst

To study the effect of temperature and reaction time on the expected catalytic activity of the mixed Co–Cr oxides prepared by SPQIR, isothermal heat treatment of compounds 1 and 2 was performed at various temperatures based on the DTG results (200–800 °C for 2 h, both in an inert and an air atmosphere.

The decomposition of compound 1 in air was completed at 250 °C (the characteristic IR bands of compound 1 had disappeared) with the formation of an amorphous Co–Cr mixed oxide phase (Fig. 8). Some new IR bands emerged in the range of *ν*(O–H, OH) and *ν*(N–H, NH_4_^+^) modes^64^ (Fig. S23).

**Fig. 8:**
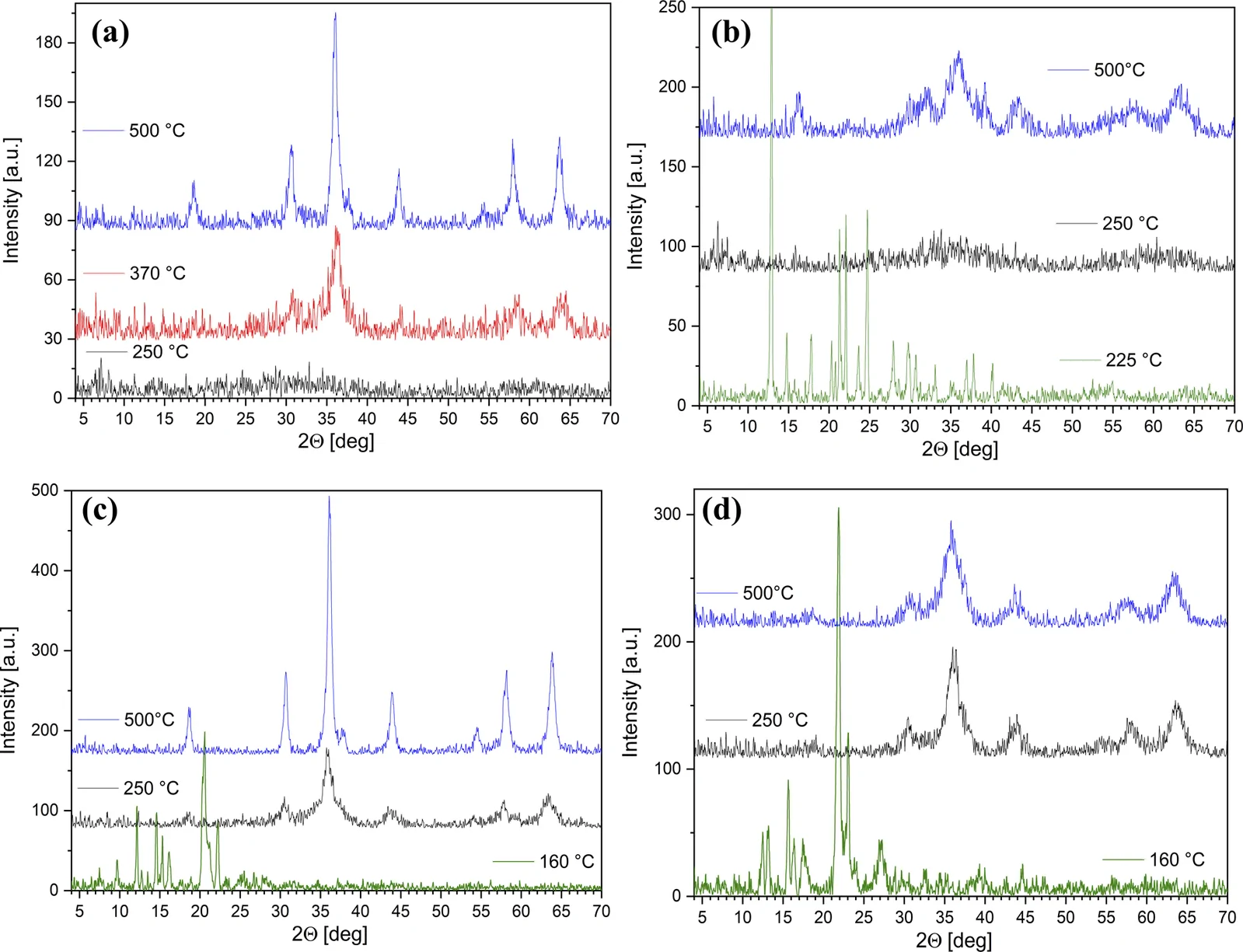
The PXRD of the thermal decomposition products, compounds 1 (a and b) and 2 (c and d), prepared in oxidative (a and c) and in inert atmosphere (b and d).

This amorphous decomposition product was leached with water, when the insoluble black residue was removed, and the evaporation of the aqueous phase resulted in crystalline CoCl_2_·6H_2_O (PDF 80-1559, Fig. S24). The presence of Co^II^ is unambiguous proof for the participation of the Co^III^ content of compound 1 in the SPQIR.

The unleached amorphous Co–Cr oxide (containing ammonium salts) was crystallized into a spinel phase with an average crystallite size of 6.5 nm on further heating; no new phase was formed, but the crystallite sizes increased up to 12 and 31 nm at 500 and 800 °C, respectively (Fig. 8 and S25). The black residue after aqueous leaching of ammonium chloride was also heated in an oxidative atmosphere at 500 °C. The PXRD of the product showed (Fig. S26) the formation of spinel Co–Cr mixed oxide (cochromite), the average crystallite size was found to be 10 nm. Based on the lack of nitrate ion (Fig. S24) and the formation of cobalt(ii) chloride on hydrolysis, it is supposed that the ammonium and chloride ions sublimed as NH_4_Cl at temperatures up to 345 °C.^68^ Nevertheless, the presence or absence of ammonium chloride must have influenced the crystallinity as was found similarly, *i.e.*, the presence of ammonium nitrate influenced the crystallinity and catalytic activity of mixed Cu–Mn oxides formed in SPQIR of [tetraamminecopper(ii)] permanganate.^5,67,69^

The inert-atmosphere heat treatment of compound 1 was performed at 225, 250, 500, and 800 °C. The decomposition intermediate, prepared at 225 °C, was a multiphase material, contained an ammine chromate complex (residual compound 1 or [Co(NH_3_)_4_CrO_4_]Cl (Fig. 8(b)), but the main decomposition reaction products also appeared, for example, in the aqueous leaching of the decomposition residue, NH_4_Cl and CoCl_2_·6H_2_O were observed (Fig. S27). The amorphous insoluble decomposition product proved to be stable, and the average crystallite size was found to be around 4 nm on heating up to 500 °C (Fig. 8(b)).

The decomposition of compound 2 was studied at 160, 250, 325, 500, and 800 °C, both in an inert and an oxidative atmosphere. The thermal decomposition of compound 2 started at 160 °C, both in air and inert atmosphere, accompanied by changes in the band shapes in the range of *ν*(Cr–O) modes, possibly due to the oxidation of the fifth ammonia and reducing the outer sphere chromate ions. The four *ν*(Cr–O) bands in the IR spectrum of compound 2 reduced to two in the IR spectrum of the decomposition product (Fig. S28). Comparison of the spectra of compound 2 having both inner and outer sphere chromate ions, with the IR spectra of [Co(NH_3_)_4_CrO_4_]Cl,^14,15^ which has only inner sphere coordinated chromate with four ammonia co-ligands, it can be concluded, that the bands attributed to the outer sphere chromate ion in compound 2 disappeared, whereas bands close to the positions of the inner sphere *ν*(Cr–O) bands of [Co(NH_3_)_4_CrO_4_]^+^ cation^14,15^ were left without decreasing in their intensity. Further studies on this supposed mechanism on the preparation and thermal decomposition of anhydrous compounds 1 and 2, [Co(NH_3_)_4_CrO_4_]Cl^36^ and [Co(NH_3_)_4_CrO_4_]_2_CrO_4_,^36^ and the dichromate-containing analogues such as [Co(NH_3_)_5_CrO_4_]_2_Cr_2_O_7_ and [Co(NH_3_)_4_]_2_Cr_2_O_7_ (ref. 36) (to increase the oxidation power of the anion towards ammonia) will be published separately.

The expected cobalt-rich Co[Co_0.2_Cr_1.8_O_3.90_] cochromite spinel could be detected even at 250 °C, both under inert and oxidative conditions (Fig. 8(c) and (d)), at much lower temperature than with other methods that used calcination at 500, 600, 700, and up to 1400 °C after solution combustion,^70^ co-precipitation,^22^ sol–gel preparation,^19^ and solid phase heating of the CoO/Co_3_O_4_ mixture with Cr_2_O_3_,^71–73^ respectively. No further phase change was observed on heating in air up to 800 °C, but the average crystallite size increased from 4.5 nm (obtained at 250 °C in air) to 6, 11 and 21 nm, at 325, 425, and 800 °C, respectively (Fig. 8 and S29). In an inert atmosphere, the cochromite spinel still detectable at 325 °C (Fig. 8(d)). The average crystallite size was found to be 3, 5 and 28 nm after heat treatments at 325, 500 and 800 °C, respectively (Fig. 8(d) and S29(b)).

### SEM measurements

The SEM measurements on compounds 1 and 2 and their decomposition products showed that the morphology of the crystallites was preserved during the whole heat treatment, independently of the atmosphere (Fig. 9 and S30–S33).

**Fig. 9:**
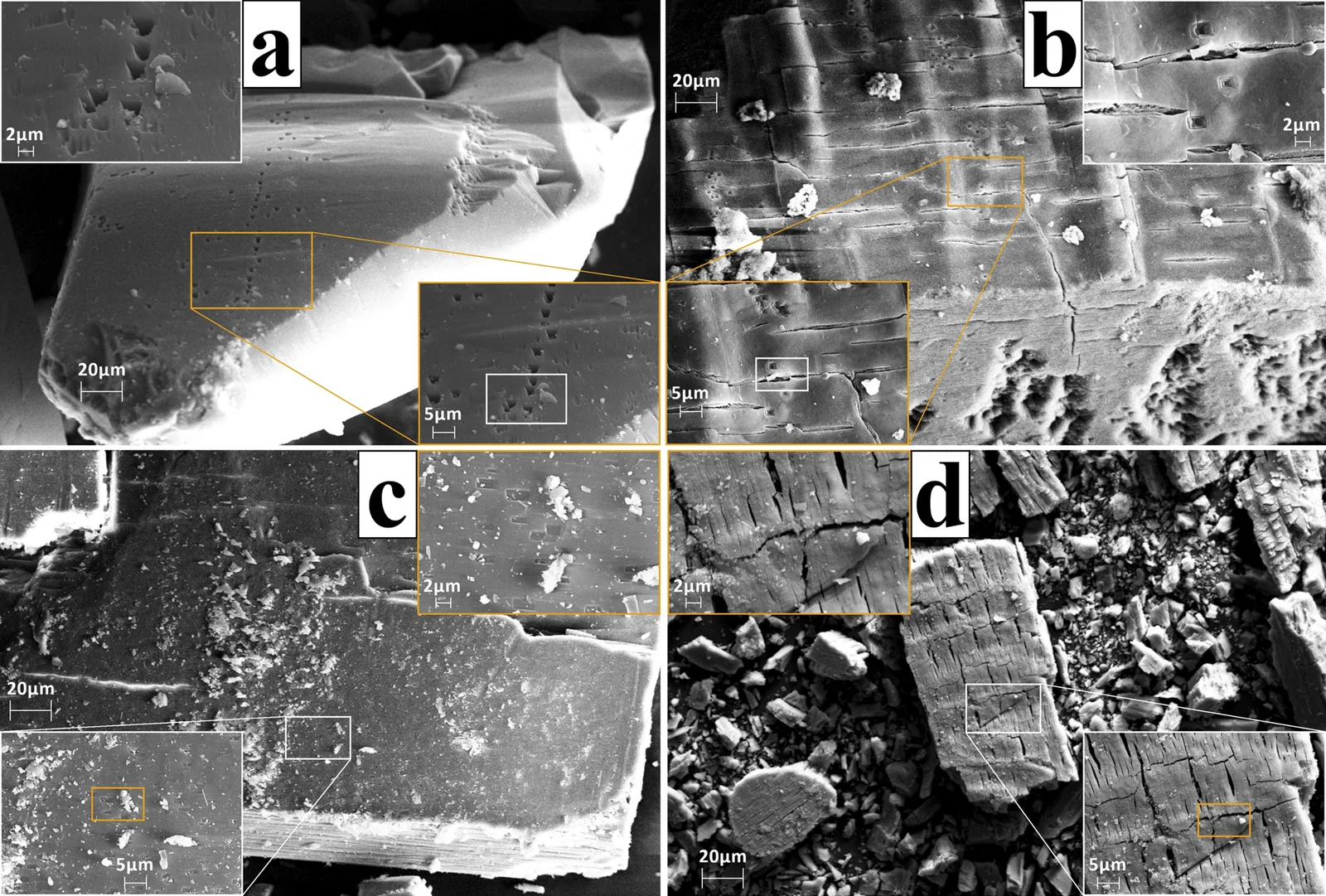
The SEM pictures of compounds 1 (a and b) and 2 (c and d) showing their starting morphology ((a) and (c)) and the change resulted by the thermal decomposition of the compounds by 250 (b) and 325 °C (d).

It also confirms the existence of SPQIR for both compounds. Fig. 9 shows that the formation (and evolution) of gases within the crystals causes cracks on the surface of the crystallites of both compounds. The increase in temperature increased the size of these cracks, which resulted in erosion of larger crystallites into smaller blocks, and eventually into nanosized oxides.

### BET surface area measurements

The BET surface area of the decomposition products of compounds 1 and 2 was determined by N_2_ adsorption–desorption measurements (the results are given in Table S18). In general, air-synthesized samples have a larger BET surface area than those prepared in an inert atmosphere, especially as the heat-treatment temperature increases. It might be attributed to the formation of Co^III^ ions from the oxidation of Co^II^ ions causing lattice distortions. Heating above 500 °C causes a decrease in the surface area of the decomposition products due to sintering of spinel phases.

Several surface area values of the decomposition products made from compounds 1 and 2 are higher than those obtained for cochromites by Chaibeddra *et al.*^22^ and comparable with the reported surface areas found by Luo *et al.*^74^ The surface areas of Co-chromites prepared in various reaction routes are summarized in Table S19.

### XPS studies

The XPS study (Table S20 and Fig. S33 (e–g)) of the decomposition product formed from compound 1 in air contained surface chloride ions, 33.2% chromium(iii), and 66.8% cobalt(iii) expressed in oxide form, without metallic cobalt. The mixed oxides prepared from compound 2 in an inert atmosphere and air contained 21.4% and 49.5% Cr^III^ and 47.7% and 36.4% Co^III^ expressed as oxides, together with 11.0% and 14.1% metallic cobalt, respectively (Table S20). The surface metallic cobalt and cobalt(iii) ions were formed due to the reduction/oxidation steps during the thermal decompositions and sample preparations, respectively.

### Photocatalytic degradation of rhodamine B

The activity of the decomposition products of compounds 1 and 2, prepared at various temperatures and atmospheres (denoted as 1-T-*i* and 2-T-*i* as well as 1-T-*a* and 2-T-*a* in inert and air atmosphere, respectively) was tested as a visible-light-driven photocatalyst^75–78^ in an advanced oxidation process, using the standard procedure with the rhodamine B dye.^75,79–81^ The photocatalytic degradation test results are summarized in Fig. 10, S34 and Table S21. The best results as catalysts in the H_2_O_2_-promoted visible-light-driven photocatalytic oxidation of rhodamine B were obtained with the samples prepared in air (Fig. 10). The highest efficiencies were found at CrO_4_-Cl-250-*a*, CrO_4_-CrO_4_-500-*a*, CrO_4_-CrO_4_-250-*a*, and CrO_4_–Cl-500-*a*, when 97.4%, 95.7%, 93.8%, and 87.1% of the rhodamine B was decomposed, respectively, (Fig. 10 and Table S20). Only a minimal dye decomposition occurred under three different conditions: (1) without a catalyst with H_2_O_2_ and visible light irradiation, (2) without H_2_O_2_ with catalysts and visible light irradiation, and (3) with catalyst and H_2_O_2_ but without visible light irradiation (Fig. S35). The CrO_4_-Cl-250-*a* catalyst, which has the best performance, has a low BET surface area (9.30 m^2^ g^−1^ (Table S20)), but an amorphous structure (Fig. 8(a)), therefore, it contains oxygen and metal vacancies, which can boost up the catalytic performance.

**Fig. 10:**
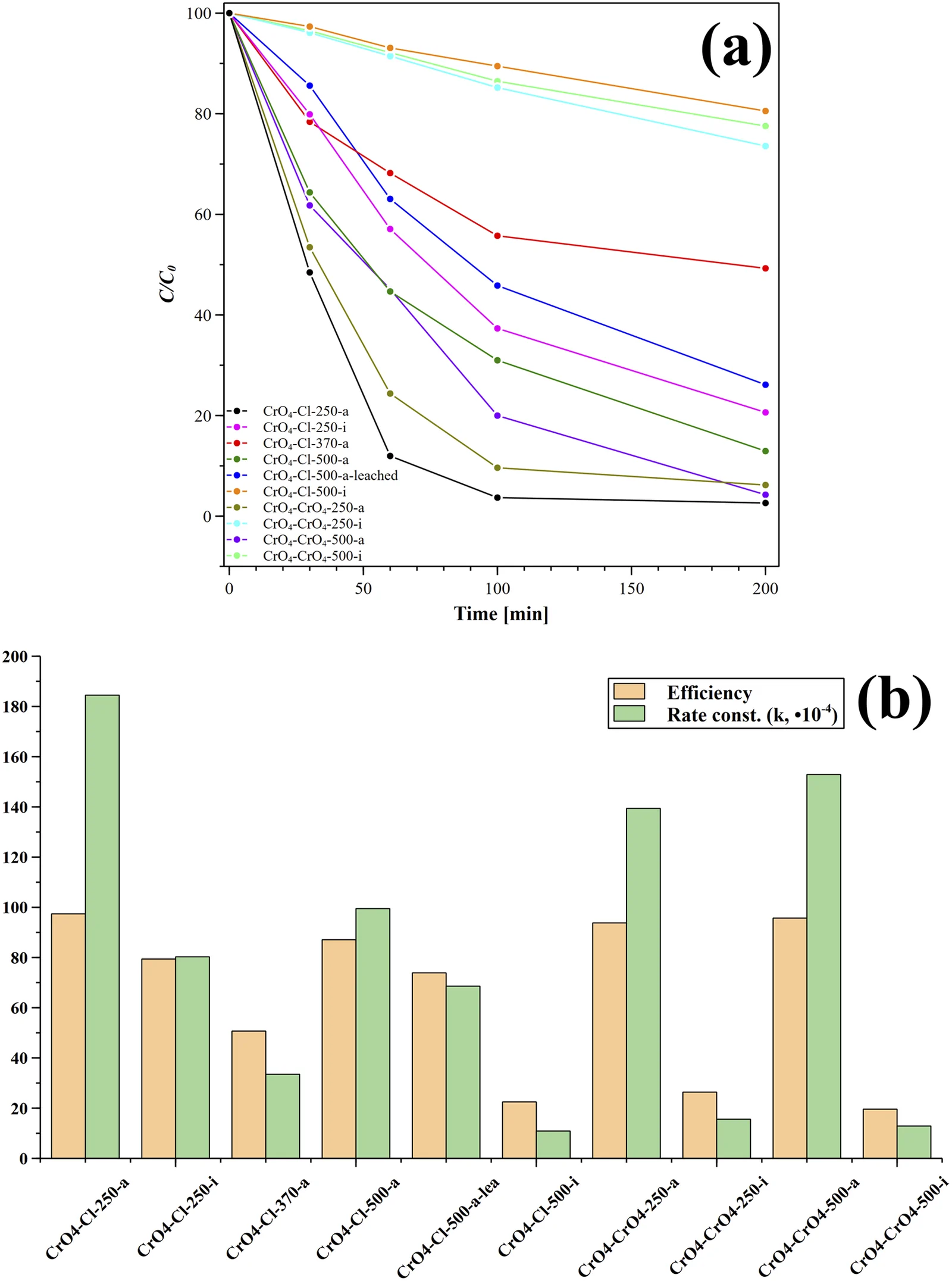
The (a) photocatalytic degradation curves and (b) photocatalytic degradation efficiency and rate constants in the photocatalytic degradation reaction of Rhodamine B performed with different Co–Cr oxides.

Despite the high activity of the amorphous decomposition products, such as CrO_4_-Cl-250-*a*, which released a large amount of chromate ions detected by ICP (Table S22), was not tested further because they proved to be not stable enough to be catalysts and released toxic chromate ions. Furthermore, the released chromate ions might also decompose the organic dye. The rate constants (*k*) of catalysts were calculated from the −ln(*C*/*C*_0_) *vs. t* plots and summarized in Table S21.

The comparison of the water-leached and non-leached samples showed that NH_4_Cl and other water-soluble components do not affect the catalytic activity (Table S21), however, leaching of Cr and Co ions was detected after adding H_2_O_2_. Therefore, we focused on the samples made at 500 °C, where no similar metal ion leaching was found.

The CrO_4_-Cl-500-*i* and CrO_4_-CrO_4_-500-*i* catalysts showed low activity (below 15%), whereas CrO_4_-Cl-500-*a* and CrO_4_-CrO_4_-500-*a*) catalysts showed superior activity (Fig. 10 and Table S21). The catalytic performance has no close relation with the BET surface area (Table S18), therefore, the difference must be accounted for by the structural parameters.^82,83^ The CrO_4_-Cl-500-*a* and CrO_4_-CrO_4_-500-*a* catalysts were tested in three consecutive steps, when they almost completely lost their original activity (remained 3 and 2%, respectively) in the 3rd cycle. The PXRD before and after catalytic reaction showed no structural change (Fig. S36). Their regeneration by heating at 500 °C for 2 h resulted in their reactivation, and their catalytic activity reached 94 and 96% of their original activities in the first cycle, respectively. This strongly suggests that the activity loss in the consecutive cycles might be attributed to the covering of active sites with small organic molecules from the degradation of the dye. We also tested the regeneration of the exhausted catalysts by washing with methanol to remove the adsorbed organic materials from the surface, with drying at 120 °C. The result. The catalyst CrO_4_-Cl-500-*a* showed 13% activity after the 2nd cycle, and washing with methanol and drying resulted in only 17% of the original catalytic activity. This showed that the adsorbed species were bound strongly to the catalyst surface.

The most likely mechanism is the oxidation of the surface Cr^III^ centres into Cr^VI^, which can absorb visible light, and the activated Cr^VI^ state reacts with the dye. Accordingly, the higher surface Co^III^ level results in better catalytic activity. Exploring the decomposition pathway of rhodamine B is also an important task. A high rate of mineralization of rhodamine B occurred, and the carbon content was converted mainly into CO_2_. The hydrogen peroxide oxidized rhodamine B to a great extent with the use of these catalysts. HPLC-MS measurements showed only one broad peak at 0.45 min retention time (Fig. S37 and S38) (The rhodamine B of high molecular mass was found at 2.18 min with 443 *m*/*z* base peak (Fig. S37)). The mass spectra showed no high molecular mass component, only a low molecular mass fragment with *m*/*z* = 112 as the base peak, together with some amount of phenol and acetic acid as fragments. The main decomposition products were identified as ammonium nitrate and carbon dioxide. This degradation route was studied previously in a detailed manner by Nguyen *et al.*^80^

Because of the positive preliminary results of the catalytic tests in decomposition of RhB, some EXAFS and XANES measurements have been performed on CrO_4_-Cl-500-*a*, CrO_4_-Cl-500-*i*, CrO_4_-CrO_4_-500-*a*, and CrO_4_-CrO_4_-500-*i* to determine the oxidation state and structural positions of the Co and Cr in the spinel structures^84,85^ (Fig. 11). During the evaluation previous XANES and EXAFS studies of Co–Cr mixed oxides were used for comparison.^86–89^ All materials have a normal spinel structure and were found to be phase pure, not a mixture of different oxide structures, which was independent of the atmosphere of their preparation. The tetrahedral sites contained only Co^II^ ions;^90^ the Co^III^ and Cr^III^ were located at OC-6 positions,^91,92^ except for the CrO_4_-Cl-500-*i* where the Co^II^ (at OC-6 positions) could not be oxidized into the trivalent state. This sample also had a very defective structure containing Cr^III^, Co^III^ and Co^II^ at the OC-6, and only Co^II^ at the T-4 sites. The XANES and EXAFS studies unambiguously confirmed the normal spinel structure of all decomposition products made at 500 °C.

**Fig. 11:**
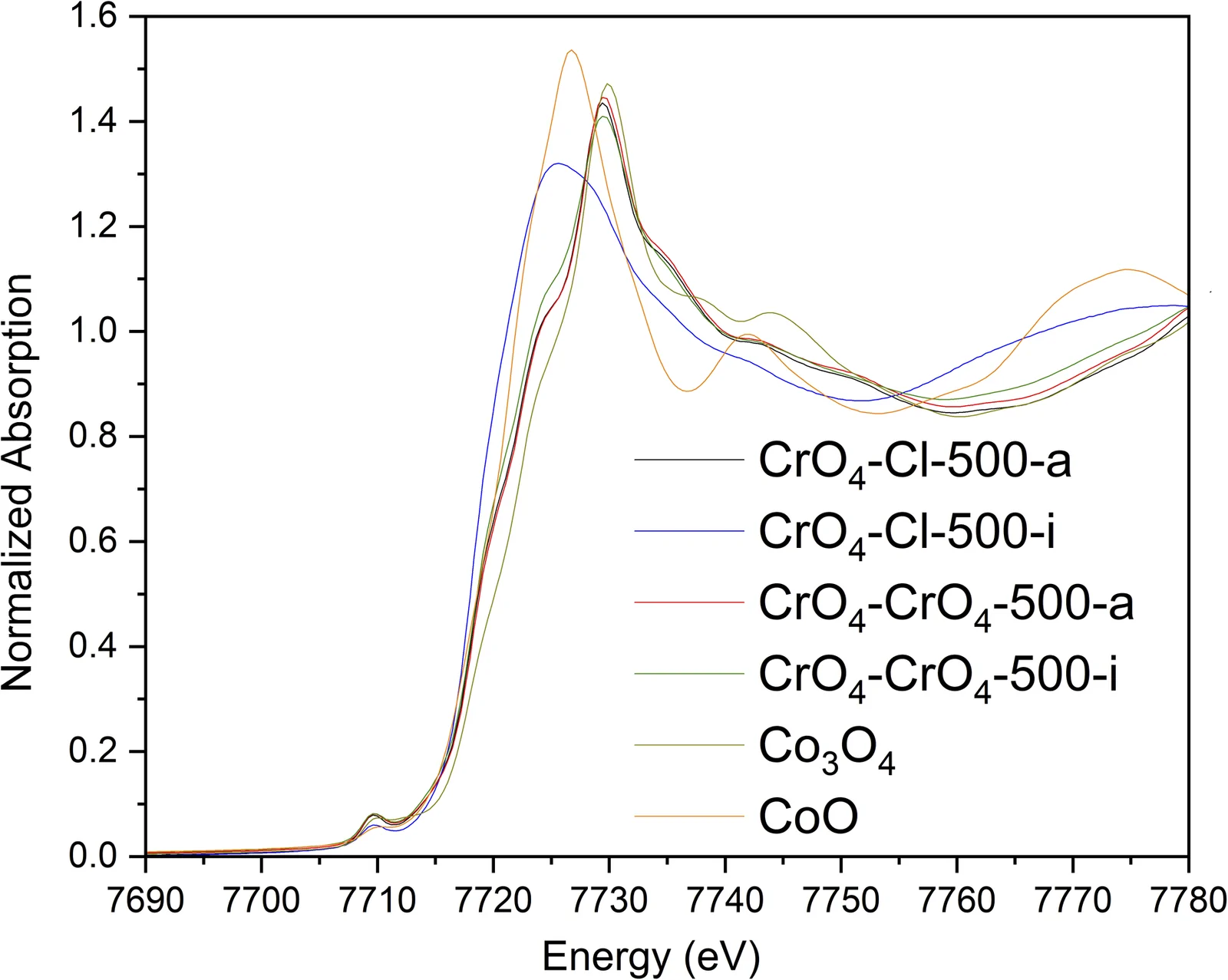
The XANES spectra of the different catalysts, Co_3_O_4_ and CoO.

This means that all the chromate ions are completely reduced to Cr^III^ during the thermal decomposition^93^ of compounds 1 and 2, whereas only a part of Co^III^ is reduced to Co^II^. The Co^II^, ions, located at OC-6 positions of these spinels, can be oxidized in air during heat treatment at 500 °C. A more detailed evaluation of XANES and EXAFS results and catalytic studies (like in the case of Gao *et al.*^95,96^) will be given in comparison with other spinels (Fe–Cr, Fe–Mn, Co–Cr and Co–Mn)^3,4,6,10,11,13^ prepared *via* SPQIR in our forthcoming submission.

## Experimental

The detailed synthesis and experimental circumstances are described in Text S5.

Crystallographic data of compound 1 and compound 2 were deposited in the Cambridge Structural Database^94^ under accession numbers 2544732 and 2544731, respectively.

## Conclusions

(1) Two coordinated chromate ion-containing complexes, [Co(NH_3_)_5_CrO_4_]Cl (compound 1) and [Co(NH_3_)_5_CrO_4_]_2_CrO_4_ (compound 2) were preparared.

The assignments of normal modes in the IR and Raman spectra have been performed with the use of correlation analysis data, including the distinction of the peaks belonging to coordinated and non-coordinated chromate ions in compound 2.

(2) The crystallographic and molecular parameters of the compounds, orthorhombic for compound 1 and monoclinic for compound 2, were determined with SCXRD at 100 K. None of the compounds exhibits any phase transition between −140 and 20 °C. The stabilization effects of hydrogen bond interactions were determined with the use of DFT calculations.

(3) The QTAIM analysis proved that the strongest interactions within the coordination sphere of the cobalt ion were formed with the chromate oxygen atom. There were no significant differences observed in the Cr–O bond strengths in coordinated and uncoordinated chromate ions in compound 2. Additional weak interactions between ammino ligands and other species in the crystal structure were observed.

(4) The extended hydrogen-bond network between the coordinated chromate ions and ammonia ligands was found to be not only a network-stabilizer but also served as a reactive site for redox reactions. Both compounds show solid-phase quasi-intramolecular redox reactions of ammonia ligands and chromate anions, with the formation of nanosized regular spinels with nominal Co to Cr molar ratios 1 : 1 (compound 1) and 2 : 3 (compound 2).

The inner-sphere coordinated chromate ions of both compounds took part in redox reactions with the residual coordinated ammonia ligands, only after the loss of one of the five ammonia ligands. However, a simple endothermic release of the first ammonia ligand was observed for compound 1, whereas an exothermic redox reaction mediated ligand destruction by the outer-sphere chromate ions was found for compound 2.

(5) The decomposition products of compounds 1 and 2 are spinels, with Co[Co_0.5_Cr_1.5_O_3.75_] and Co[Co_0.2_Cr_1.8_O_3.90_] compositions, respectively, and the cobalt(iii) content of both complexes is partially reduced to cobalt(ii). The cobalt(ii) ions are located at the tetrahedral positions in all four spinels, and a substantial amount is found at T-4 positions in the 1 : 1 spinel made in an inert atmosphere, resulting in a strongly defective structure. All the Co^III^ and Cr^III^ ions are located only in octahedral lattice positions in all four spinels.

(6) The chromate ions of compounds 1 and 2 are completely reduced to Cr^III^ during the thermal decomposition, whereas only a part of Co^III^ is reduced to Co^II^. Furthermore, the reduction of Co^III^ to Co^II^ depends on the atmosphere and Co to Cr ratio as well. At Co to Cr ratio 2 : 3, the atmosphere had negligible effect; in both inert and air atmospheres, the T-4 and OC-6 sites of the decomposition products contain only Co^II^ and Co^III/^Cr^III^, respectively. However, at Co to Cr ratio 1 : 1 under N_2_, the products contained both Co^II^ and Co^III^ at the OC-6 sites, this Co^II^ content oxidized in air into Co^III^. The T-4 sites contained only Co^II^ in the products made eitherin an inert or air atmosphere, even at Co to Cr ratio 1 : 1.

(7) The decomposition products of compounds 1 and 2 were proved to be active catalysts in the visible-light-driven photocatalytic oxidation of rhodamine B with H_2_O_2_. The surface characterization of the *in situ* formed nanocatalysts prepared at various temperatures from compounds 1 and 2, and the kinetic parameters of rhodamine B degradation in the presence of these catalysts were evaluated in detail. The most active non-amorphous catalyst was prepared from compound 1 at 500 °C, and its superior activity might be attributed to the presence of Co^II^ ions at octahedral sites and to its strongly defective structure.

## Author contributions

Bálint Tulik: writing – editing, visualization, validation, methodology, investigation, formal analysis, data curation. Zsolt Dürvanger: writing – review & editing, investigation, methodology, formal analysis, data curation. Shiro Kubuki: writing – review, validation, methodology, investigation, formal analysis, data curation. Attila Farkas: visualization, validation, methodology, investigation. Stjepko Krehula: validation, methodology, investigation, formal analysis, data curation. Vladimir M. Petruševski: writing – review & editing, validation, methodology, investigation, formal analysis. Laura Bereczki: writing – review & editing, investigation, methodology, formal analysis, data curation. Zoltán Homonnay: writing – review & editing & grammar, visualization, methodology. Milica Marković: visualization, methodology, investigation, formal analysis, data curation. Aleksandra Rakic: visualization, validation, methodology, investigation, formal analysis, data curation. Dušan Dimić: writing – review & editing, visualization validation, methodology, investigation, formal analysis, data curation. László Trif: visualization, validation, investigation, formal analysis, data curation. Zoltán Károly: writing – review & editing, visualization, methodology, resources, project administration. László Kótai: writing – review & editing, writing – original draft, visualization, validation, supervision, resources, methodology, investigation, funding acquisition, formal analysis, data curation, conceptualization. Kende Attila Béres: writing – review & editing, writing – original draft, visualization, validation, resources, methodology, investigation, funding acquisition, formal analysis, data curation, conceptualization.

## Conflicts of interest

There are no conflicts to declare.

## Supplementary Material

RA-016-D6RA06540F-s001

## Data Availability

CCDC 2544731 (1) and 2544732 (2) contain the supplementary crystallographic data for this paper.^97*a*,*b*^ The data supporting this article are available in Zenodo at https://doi.org/10.5281/zenodo.21301067. Additional experimental details and characterization data are provided in the supplementary information (SI). Contents to upload to a data repository • Raw characterization data (XRD, IR, Raman) • Catalytic testing data (Origin) • Crystal structure files (CIF). Supplementary information is available. See DOI: https://doi.org/10.1039/d6ra06540f.
