# Supplementary material for: The reactivity of coordinated and uncoordinated chromate ions in the quasi-intramolecular redox reactions of [pentaamminechromatocobalt(iii)] chloride and chromate resulting in tuneable cobalt-chromium spinel oxide catalysts
Source: RSC Adv. 2026 Oct 23;16(48):65627–41. doi: 10.1039/d6ra06540f (PMC13693807; doi:10.1039/d6ra06540f)
Supplement: Supplementary file 1 [file RA-016-D6RA06540F-s001.pdf]

Supplementary Information (SI) for RSC Advances.
This journal is © The Royal Society of Chemistry

Electronic Supplementary Information

The reactivity of coordinated and uncoordinated chromate ions in the quasi-intramolecular redox reactions of [pentaamminechromatocobalt(III)] chloride and chromate resulting in tuneable cobalt-chromium spinel oxide catalysts

Bálint Tulik,[a,b] Zsolt Dürvanger,[c,d] Shiro Kubuki,[e] Attila Farkas,[f] Stjepko Krehula,[g] Vladimir M. Petruševski,[h] Laura Bereczki,[a] Zoltán Homonnay,[i] Milica Marković,[j] Aleksandra Rakic,[j] Dušan Dimić,[j] László Trif,[a] Zoltán Károly,[a] László Kótai,[a,] and Kende Attila Béres[a]*

a) *Institute of Materials and Environmental Chemistry, HUN-REN Research Centre for Natural Sciences, Budapest, Hungary. E-mail: tulik.balint@ttk.hu, beres.kende.attila@ttk.hu, bereczki.laura@ttk.hu, trif.laszlo@ttk.hu, karoly.zoltan@ttk.hu*

b) *Department of Physical Chemistry and Materials Science, Faculty of Chemical Technology and Biotechnology, Budapest University of Technology and Economics, Budapest, Hungary.*

c) *Laboratory of Structural Chemistry and Biology, Institute of Chemistry, ELTE Eötvös Loránd University, Budapest, Hungary. zsolt.durvanger@ttk.elte.hu*

d) *HUN-REN-ELTE Protein Modelling Research Group, Budapest, Hungary*

e) *Department of Chemistry, Graduate School of Science, Tokyo Metropolitan University, 1-1 Minami-Osawa, Hachi-Oji, Tokyo, 192-0397, Japan. kubuki@tmu.ac.jp*

f) *Department of Organic Chemistry and Technology, Faculty of Chemical Technology and Biotechnology, Budapest University of Technology and Economics, Budapest, Hungary. farkas.attila@vbk.bme.hu*

g) *Division of Materials Chemistry, Ruđer Bošković Institute, Zagreb, Croatia. E-mail: Stjepko.Krehula@irb.hr*

h) *Institute of Chemistry, Faculty of Natural Sciences and Mathematics, Ss. Cyril and Methodius University, Skopje, Republic of North Macedonia. E-mail: vladimirpetrusevski@yahoo.com*

i) *Institute of Chemistry, ELTE Eötvös Loránd University, Budapest, Hungary. E-mail: homonnay.zoltan@ttk.elte.hu*

j) *Faculty of Physical Chemistry, University of Belgrade, Belgrade, Serbia. E-mail: milica@bio.bg.ac.rs, saska@ffh.bg.ac.rs, dusan.dimic@ffh.bg.ac.rs*

***Correspondence:** *kotai.laszlo@ttk.hu*

Text S1. Magnetic susceptibility measurements

The effective magnetic moment values of compounds **1** and **2** were calculated from the room-temperature magnetic susceptibility measurements and found to be 0.43 and 0.85 B.M., respectively, as expected for the diamagnetic low-spin d^6 Co^{III} compounds. These are close to the μ_{eff} value of $[Co(NH_3)_5CrO_4]ClO_4$ (compound **4**, μ_{eff} =0.62 B.M.[1]), in the case of compound **1**. The same cationic contribution is expected in compounds **1**, **2,** and **4**, due to the same complex cation involved; thus, the temperature-independent paramagnetic contribution of the outer sphere chromate ion is equal to that of two chloride ions in two moles of compound **1** (2×0.43=0.86 is very close to the value of 0.85 found for compound **2**).

Text S2. UV-VIS spectroscopy

The ground state electron configuration of the diamagnetic (low spin) octahedral d^6 Co^{III} ion is t_{2g}^6 ($^1A_{1g}$). The $t_{2g}^6 \rightarrow t_{2g}^5 e_g^1$ spin-allowed electron excitation generates two excited singlet terms, $^1T_{1g}$ and $^1T_{2g}$. These transitions are Laporte-forbidden and spin allowed (singlet to singlet) ones, so their intensities are expected to be weak and are overlapped with the intense LMCT bands of chromate ion ($O_{2p} \rightarrow Cr_{3d}$ bands). The solid phase UV-VIS data of compounds **1** and **2** showed that the first band ($^1A_{1g} \rightarrow {}^1T_{1g}$) of the $[Co(NH_3)_5CrO_4]^+$ cation is at 582 and 570 nm, respectively (Fig. S1). The shift of this *d-d* band may be attributed to the change in the ligand field splitting due to stronger electrostatic interactions between the chromate(2-) anion compared to the monovalent chloride anion, and due to the presence of more hydrogen bonds in compound **2** than in compound **1**, which results in a reduced ability of the ligand field to relax. The $^1A_{1g} \rightarrow {}^1T_{2g}$ transition is overlapped with the $\pi \rightarrow \pi^*$ band of chromate ion; accordingly, two partially overlapping bands are observed in the UV-VIS spectra of compounds **1** and **2** at 355/311 and 364/342 cm^{-1}, respectively. Tsuchida[2,3] found the bands for the aqueous solution for both the chloride and nitrate complexes at 540 and 383 nm, whereas Yonade found three bands for the solutions of this complex at 545, 429, and 375 nm [4]. The $CrO_4^{2-} \rightarrow Co^{III}$ LMCT band in the solid compounds **1** and **2** was found at 462 and 457 nm, respectively. The ammonia ligand-centered band, together with other $CrO_4^{2-} \rightarrow Co^{III}$ LMCT bands with different orbital symmetries, is located around 220/231 and 260 cm^{-1} in the spectra of compounds **1** and **2**, respectively.

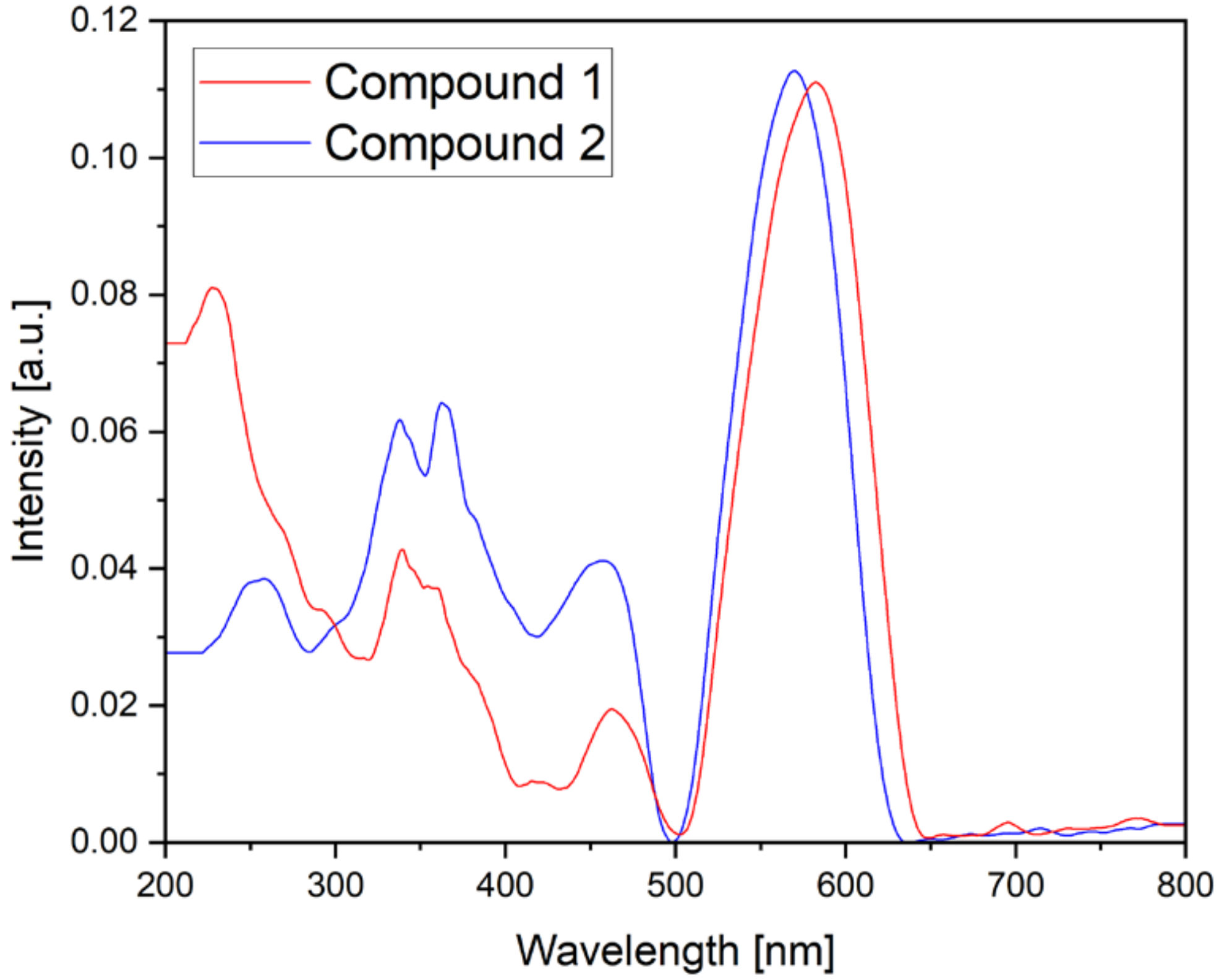

Figure S1. The solid phase UV spectra of compounds **1** and **2.**

Table S1. Crystallographic data for compounds **1** and **2**.

Compound	$[Co(NH_3)_5CrO_4]Cl\cdot H_2O$	$[Co(NH_3)_5CrO_4]_2CrO_4\cdot 4H_2O$
Acronym	Compound **1**	Compound **2**
Empirical formula	$CoCrClN_5O_5H_{17}$	$Co_2Cr_3N_{10}O_{16}H_{38}$
Formula weight	313.57	708.26
Temperature (K)	103(2)	108(2)
Radiation and wavelength (Å)	Mo-Kα, λ =0.71073	Cu-Kα, 1.54184
Crystal system	Orthorhombic	Monoclinic
Space group	*Pnma*	$P2_1/c$
Unit cell dimensions (Å and °)	*a*=9.9747(8)<br>*b*=8.2197(8)<br>*c*=12.7822(12)	*a* = 15.7663(2) *b* = 7.08120(10)<br>*c* = 20.6427(2)<br>*β*= 93.9040(10) °
Volume ($Å^3$)	1048.00(16)	2299.29(5)
Z	4	4
Density (calculated, Mg/m^3)	1.987	2.046

Table S2. Data collection and refinement parameters for compounds **1** and **2**.

Compound	$[Co(NH_3)_5CrO_4]Cl\cdot H_2O$	$[Co(NH_3)_5CrO_4]_2CrO_4\cdot 4H_2O$
F(000)	640	1448
Crystal size	0.337 x 0.329 x 0.232 mm^3	0.145 x 0.061 x 0.021 mm^3
Theta range for data collection	5.186 to 26.372°.	2.809 to 75.545°.
Index ranges	-12<=h<=12, -10<=k<=10, -15<=l<=15	-19<=h<=19, -8<=k<=8, -25<=l<=21
Reflections collected	14599	38932
Independent reflections	1138 [*R*(int) = 0.0648]	4718 [R(int) = 0.0460]
Completeness to theta = 25.242°	98.9 %	100.0 %
Absorption correction	Numerical	Analytical
Max. and min. transmission	0.902 and 0.846	0.709 and 0.216
Refinement method	Full-matrix least-squares on F^2	Full-matrix least-squares on F^2
Data / restraints / parameters	1138 / 0 / 73	4718 / 0 / 302
Goodness-of-fit on F^2	1.242	1.099
Final R indices [I>2sigma(I)]	R1 = 0.0406, wR2 = 0.0769	R1 = 0.0526, wR2 = 0.1278
R indices (all data)	R1 = 0.0462, wR2 = 0.0787	R1 = 0.0586, wR2 = 0.1316
Extinction coefficient	n/a	n/a
Largest diff. peak and hole	0.396 and -0.424 e. $Å^{-3}$	1.229 and -0.723 e·$Å^{-3}$

Table S3. Bond lengths [Å] and angles [°] in compound **1**.

Co(1)-O(1)	1.917(3)
Co(1)-N(2)	1.948(4)
Co(1)-N(3)	1.955(3)
Co(1)-N(3)#1	1.955(3)
Co(1)-N(1)#1	1.967(3)
Co(1)-N(1)	1.967(3)
Cr(1)-O(2)	1.626(3)
Cr(1)-O(3)#1	1.641(2)
Cr(1)-O(3)	1.641(2)
Cr(1)-O(1)	1.699(3)
N(1)-H(1A)	0.8900
N(1)-H(1B)	0.8900
N(1)-H(1C)	0.8900
N(3)-H(3A)	0.8900
N(3)-H(3B)	0.8900
N(3)-H(3C)	0.8900
N(2)-H(2A)	0.8900
N(2)-H(2B)	0.8900
N(2)-H(2C)	0.8900
N(2)-H(2A)#1	0.890(17)
N(2)-H(2B)#1	0.89(2)
N(2)-H(2C)#1	0.89(4)
O(4)-H(4A)	0.8457
O(4)-H(4B)	0.8489
O(4)-H(4A)#2	0.846(4)
O(4)-H(4B)#2	0.849(4)
O(1)-Co(1)-N(2)	178.69(15)
O(1)-Co(1)-N(3)	88.05(11)
N(2)-Co(1)-N(3)	91.04(11)
O(1)-Co(1)-N(3)#1	88.04(11)
N(2)-Co(1)-N(3)#1	91.04(11)
N(3)-Co(1)-N(3)#1	90.53(17)
O(1)-Co(1)-N(1)#1	90.51(11)
N(2)-Co(1)-N(1)#1	90.41(11)
N(3)-Co(1)-N(1)#1	178.55(12)

N(3)#1-Co(1)-N(1)#1	89.29(12)
O(1)-Co(1)-N(1)	90.51(11)
N(2)-Co(1)-N(1)	90.41(11)
N(3)-Co(1)-N(1)	89.29(12)
N(3)#1-Co(1)-N(1)	178.55(12)
N(1)#1-Co(1)-N(1)	90.86(16)
O(2)-Cr(1)-O(3)#1	110.95(11)
O(2)-Cr(1)-O(3)	110.95(11)
O(3)#1-Cr(1)-O(3)	107.53(18)
O(2)-Cr(1)-O(1)	110.45(17)
O(3)#1-Cr(1)-O(1)	108.43(11)
O(3)-Cr(1)-O(1)	108.43(11)
Cr(1)-O(1)-Co(1)	134.14(19)
Co(1)-N(1)-H(1A)	109.5
Co(1)-N(1)-H(1B)	109.5
H(1A)-N(1)-H(1B)	109.5
Co(1)-N(1)-H(1C)	109.5
H(1A)-N(1)-H(1C)	109.5
H(1B)-N(1)-H(1C)	109.5
Co(1)-N(3)-H(3A)	109.5
Co(1)-N(3)-H(3B)	109.5
H(3A)-N(3)-H(3B)	109.5
Co(1)-N(3)-H(3C)	109.5
H(3A)-N(3)-H(3C)	109.5
H(3B)-N(3)-H(3C)	109.5
Co(1)-N(2)-H(2A)	109.5
Co(1)-N(2)-H(2B)	109.5
H(2A)-N(2)-H(2B)	109.5
Co(1)-N(2)-H(2C)	109.5
H(2A)-N(2)-H(2C)	109.5
H(2B)-N(2)-H(2C)	109.5
Co(1)-N(2)-H(2A)#1	109.5(4)
H(2A)-N(2)-H(2A)#1	132.5
H(2B)-N(2)-H(2A)#1	81.7
H(2C)-N(2)-H(2A)#1	30.3
Co(1)-N(2)-H(2B)#1	109.5(5)
H(2A)-N(2)-H(2B)#1	81.7
H(2B)-N(2)-H(2B)#1	30.3

H(2C)-N(2)-H(2B)#1	132.5
H(2A)#1-N(2)-H(2B)#1	109.5
Co(1)-N(2)-H(2C)#1	109.5(9)
H(2A)-N(2)-H(2C)#1	30.3
H(2B)-N(2)-H(2C)#1	132.5
H(2C)-N(2)-H(2C)#1	81.7
H(2A)#1-N(2)-H(2C)#1	109.5
H(2B)#1-N(2)-H(2C)#1	109.5
H(4A)-O(4)-H(4B)	110.1
H(4A)-O(4)-H(4A)#2	17.9
H(4B)-O(4)-H(4A)#2	107.2
H(4A)-O(4)-H(4B)#2	107.2
H(4B)-O(4)-H(4B)#2	17.9
H(4A)#2-O(4)-H(4B)#2	110.1

Symmetry transformations used to generate equivalent atoms:

#1 x,-y+3/2,z   #2 x,-y+1/2,z

Table S4. Torsion angles [°] in compound **1**.

O(2)-Cr(1)-O(1)-Co(1)	0.000(1)
O(3)#1-Cr(1)-O(1)-Co(1)	-121.77(10)
O(3)-Cr(1)-O(1)-Co(1)	121.77(10)

Symmetry transformations used to generate equivalent atoms:

#1 x,-y+3/2,z #2 x,-y+1/2,z

Table S5. Hydrogen bonds in compound **1**.

D-H...A	d(D-H)	d(H...A)	d(D...A)	<(DHA)
N(1)-H(1A)...O(3)#3	0.89	2.15	3.000(3)	159.8
N(1)-H(1B)...O(2)	0.89	2.33	3.087(4)	143.4
N(1)-H(1C)...Cl(1)	0.89	2.49	3.373(3)	174.2
N(3)-H(3A)...O(3)#4	0.89	2.14	2.995(4)	162.0
N(3)-H(3B)...Cl(1)	0.89	2.69	3.407(3)	138.5
N(3)-H(3C)...O(2)#5	0.89	2.41	3.111(4)	136.3
N(3)-H(3C)...O(4)#4	0.89	2.25	2.909(5)	130.9
N(2)-H(2A)...O(3)#6	0.89	2.02	2.889(3)	166.6
N(2)-H(2B)...O(3)#3	0.89	2.27	3.136(4)	164.4
N(2)-H(2B)...O(3)#7	0.89	2.53	3.136(4)	126.3
N(2)-H(2C)...O(3)#4	0.89	2.22	2.889(3)	131.8
O(4)-H(4A)...Cl(1)#3	0.85	2.24	3.070(4)	167.1
O(4)-H(4B)...Cl(1)	0.85	2.25	3.079(4)	167.2

Symmetry transformations used to generate equivalent atoms:

#1 x,-y+3/2,z #2 x,-y+1/2,z #3 x-1/2,y,-z+1/2

#4 -x+1/2,-y+1,z-1/2 #5 x+1/2,y,-z+1/2 #6 -x+1/2,y+1/2,z-1/2

#7 x-1/2,-y+3/2,-z+1/2

Table S6. Bond lengths [Å] and angles [°] in compound **2**.

Co(1A)-O(1A)	1.899(3)
Co(1A)-N(1A)	1.944(4)
Co(1A)-N(2A)	1.950(4)
Co(1A)-N(3A)	1.950(4)
Co(1A)-N(4A)	1.962(4)
Co(1A)-N(5A)	1.967(4)
Co(1B)-O(1B)	1.917(3)
Co(1B)-N(2B)	1.946(4)
Co(1B)-N(3B)	1.949(4)
Co(1B)-N(1B)	1.959(4)
Co(1B)-N(4B)	1.962(4)
Co(1B)-N(5B)	1.969(4)
Cr(1A)-O(3A)	1.622(3)
Cr(1A)-O(2A)	1.647(3)
Cr(1A)-O(4A)	1.648(3)
Cr(1A)-O(1A)	1.692(3)
Cr(1B)-O(2B)	1.633(3)
Cr(1B)-O(3B)	1.635(3)
Cr(1B)-O(4B)	1.654(4)
Cr(1B)-O(1B)	1.697(3)
Cr(3)-O(12)	1.610(4)
Cr(3)-O(10)	1.626(5)
Cr(3)-O(9)	1.636(5)
Cr(3)-O(11)	1.672(4)
O(1A)-Co(1A)-N(1A)	177.68(15)
O(1A)-Co(1A)-N(2A)	88.55(15)
N(1A)-Co(1A)-N(2A)	90.47(17)
O(1A)-Co(1A)-N(3A)	89.36(16)
N(1A)-Co(1A)-N(3A)	92.70(17)
N(2A)-Co(1A)-N(3A)	87.75(17)
O(1A)-Co(1A)-N(4A)	89.99(16)
N(1A)-Co(1A)-N(4A)	91.00(18)
N(2A)-Co(1A)-N(4A)	178.50(18)
N(3A)-Co(1A)-N(4A)	91.87(17)
O(1A)-Co(1A)-N(5A)	87.68(16)
N(1A)-Co(1A)-N(5A)	90.25(17)

N(2A)-Co(1A)-N(5A)	91.48(16)
N(3A)-Co(1A)-N(5A)	176.96(17)
N(4A)-Co(1A)-N(5A)	88.83(17)
O(1B)-Co(1B)-N(2B)	88.84(16)
O(1B)-Co(1B)-N(3B)	91.25(16)
N(2B)-Co(1B)-N(3B)	87.32(18)
O(1B)-Co(1B)-N(1B)	179.41(17)
N(2B)-Co(1B)-N(1B)	91.65(18)
N(3B)-Co(1B)-N(1B)	89.09(18)
O(1B)-Co(1B)-N(4B)	89.69(16)
N(2B)-Co(1B)-N(4B)	178.52(18)
N(3B)-Co(1B)-N(4B)	92.87(18)
N(1B)-Co(1B)-N(4B)	89.82(19)
O(1B)-Co(1B)-N(5B)	88.16(16)
N(2B)-Co(1B)-N(5B)	90.42(18)
N(3B)-Co(1B)-N(5B)	177.68(18)
N(1B)-Co(1B)-N(5B)	91.52(18)
N(4B)-Co(1B)-N(5B)	89.37(18)
O(3A)-Cr(1A)-O(2A)	109.79(17)
O(3A)-Cr(1A)-O(4A)	108.22(17)
O(2A)-Cr(1A)-O(4A)	110.37(17)
O(3A)-Cr(1A)-O(1A)	110.36(17)
O(2A)-Cr(1A)-O(1A)	108.08(16)
O(4A)-Cr(1A)-O(1A)	110.03(16)
O(2B)-Cr(1B)-O(3B)	109.46(18)
O(2B)-Cr(1B)-O(4B)	111.16(18)
O(3B)-Cr(1B)-O(4B)	106.99(18)
O(2B)-Cr(1B)-O(1B)	108.61(17)
O(3B)-Cr(1B)-O(1B)	110.94(17)
O(4B)-Cr(1B)-O(1B)	109.69(17)
O(12)-Cr(3)-O(10)	110.0(3)
O(12)-Cr(3)-O(9)	110.9(3)
O(10)-Cr(3)-O(9)	110.9(3)
O(12)-Cr(3)-O(11)	107.2(2)
O(10)-Cr(3)-O(11)	110.5(3)
O(9)-Cr(3)-O(11)	107.3(3)
Cr(1A)-O(1A)-Co(1A)	138.19(18)
Cr(1B)-O(1B)-Co(1B)	131.67(19)

Table S7. Torsion angles [°] in compound **2**.

O(3A)-Cr(1A)-O(1A)-Co(1A)	-62.4(3)
O(2A)-Cr(1A)-O(1A)-Co(1A)	177.5(3)
O(4A)-Cr(1A)-O(1A)-Co(1A)	56.9(3)
N(2A)-Co(1A)-O(1A)-Cr(1A)	136.2(3)
N(3A)-Co(1A)-O(1A)-Cr(1A)	48.4(3)
N(4A)-Co(1A)-O(1A)-Cr(1A)	-43.5(3)
N(5A)-Co(1A)-O(1A)-Cr(1A)	-132.3(3)
O(2B)-Cr(1B)-O(1B)-Co(1B)	-178.2(2)
O(3B)-Cr(1B)-O(1B)-Co(1B)	-57.9(3)
O(4B)-Cr(1B)-O(1B)-Co(1B)	60.1(3)

Table S8. Hydrogen bonds in compound **2**.

D-H...A	d(D-H)	d(H...A)	d(D...A)	<(DHA)
N(2A)-H(2AA)...O(2A)#1	0.91	2.07	2.952(5)	163.5
N(2A)-H(2AB)...O(1A)#2	0.91	2.51	3.080(5)	121.5
N(2A)-H(2AB)...O(2A)#2	0.91	2.47	3.154(5)	131.9
N(2A)-H(2AC)...O(2B)#3	0.91	2.12	2.995(5)	161.4
N(3A)-H(3AA)...O(4A)#4	0.91	2.10	2.981(5)	162.4
N(3A)-H(3AB)...O(4A)#1	0.91	2.12	3.022(5)	171.5
N(3A)-H(3AC)...O(3B)#3	0.91	2.16	3.050(5)	166.5
N(2B)-H(2BA)...O(3B)#5	0.91	2.60	3.112(5)	116.6
N(2B)-H(2BA)...O(4B)#5	0.91	2.24	3.098(5)	157.2
N(2B)-H(2BB)...O(2A)#2	0.91	2.09	2.978(5)	164.3
N(2B)-H(2BC)...O(11)#5	0.91	2.06	2.957(6)	166.7
N(3B)-H(3BA)...O(3B)	0.91	2.36	2.987(5)	126.0
N(3B)-H(3BA)...O(3A)#2	0.91	2.10	2.937(5)	151.7
N(3B)-H(3BB)...O(16)#6	0.91	2.02	2.923(6)	172.9
N(3B)-H(3BC)...O(11)#5	0.91	2.21	3.008(6)	145.4
N(3B)-H(3BC)...O(9)#5	0.91	2.53	3.329(6)	146.6
N(5B)-H(5BA)...O(12)#7	0.91	2.11	2.939(6)	151.7
N(5B)-H(5BB)...O(4B)#5	0.91	2.13	3.022(6)	164.9
N(5B)-H(5BC)...O(15)#8	0.91	2.12	3.001(6)	162.5
N(1A)-H(1AA)...O(4A)#1	0.91	2.37	3.099(5)	137.0
N(1A)-H(1AA)...O(13)#7	0.91	2.39	3.023(6)	126.5
N(1A)-H(1AB)...O(12)#7	0.91	2.22	3.074(6)	156.9
N(1A)-H(1AC)...O(2B)	0.91	2.01	2.913(5)	174.3
N(4A)-H(4AA)...O(11)#7	0.91	2.45	3.112(6)	129.8
N(4A)-H(4AB)...O(4A)	0.91	2.29	3.100(6)	147.6
N(4A)-H(4AC)...O(14)#9	0.91	2.13	2.996(7)	158.9
N(5A)-H(5AA)...O(12)#7	0.91	2.11	3.000(6)	165.9
N(5A)-H(5AB)...O(2B)#5	0.91	2.23	3.116(6)	162.9
N(5A)-H(5AC)...O(2A)#2	0.91	2.16	3.022(5)	158.7
N(1B)-H(1BA)...O(15)#8	0.91	2.45	3.289(6)	153.8
N(1B)-H(1BA)...O(10)#8	0.91	2.59	3.030(7)	110.4
N(1B)-H(1BB)...O(16)#8	0.91	2.18	3.082(6)	173.0
N(4B)-H(4BA)...O(15)#7	0.91	2.13	2.992(6)	157.5
N(4B)-H(4BB)...O(9)#6	0.91	2.09	2.988(6)	168.0
N(4B)-H(4BC)...O(4B)	0.91	2.39	2.977(6)	122.2

N(4B)-H(4BC)...O(16)#6	0.91	2.49	3.146(6)	128.8

Symmetry transformations used to generate equivalent atoms:

#1 x,y+1,z   #2 -x+1,y+1/2,-z+1/2   #3 -x+1,y-1/2,-z+1/2

#4 -x+1,-y,-z   #5 x,y-1,z   #6 -x+2,-y+2,-z+1

#7 x,-y+3/2,z-1/2   #8 -x+2,-y+1,-z+1   #9 x,-y+1/2,z-1/2

Table S9. Experimental and theoretical (at the DFT/B3LYP/6-311+G(d,p)(H,N,O,Cl)/LanL2DZ(Co,Cr) level of theory) bond lengths (in Å) of compound **1.**

Bond	Experimental	Theoretical
Co(1)-O(1)	1.917(3)	1.887
Co(1)-N(2)	1.948(4)	1.980
Co(1)-N(3)	1.955(3)	1.985
Co(1)-N(3)#1	1.955(3)	1.999
Co(1)-N(1)#1	1.967(3)	2.027
Co(1)-N(1)	1.967(3)	2.030
Cr(1)-O(2)	1.626(3)	1.584
Cr(1)-O(3)#1	1.641(2)	1.618
Cr(1)-O(3)	1.641(2)	1.635
Cr(1)-O(1)	1.699(3)	1.768
R		0.98
MAE [Å]		0.04

Table S10. Experimental and theoretical (at the DFT/B3LYP/6-311+G(d,p)(H,N,O,Cl)/LanL2DZ(Co,Cr) level of theory) bond angles (in °) of compound **1.**

Bond angles	Experimental	Theoretical
O(1)-Co(1)-N(2)	178.69(15)	173.80
O(1)-Co(1)-N(3)	88.05(11)	86.37
N(2)-Co(1)-N(3)	91.04(11)	85.38
O(1)-Co(1)-N(3)#1	88.04(11)	79.76
N(2)-Co(1)-N(3)#1	91.04(11)	89.28
N(3)-Co(1)-N(3)#1	90.53(17)	92.28
O(1)-Co(1)-N(1)#1	90.51(11)	86.97
N(2)-Co(1)-N(1)#1	90.41(11)	92.05
N(3)-Co(1)-N(1)#1	178.55(12)	175.71
N(3)#1-Co(1)-N(1)#1	89.29(12)	94.26
O(1)-Co(1)-N(1)	90.51(11)	96.63
N(2)-Co(1)-N(1)	90.41(11)	94.89
N(3)-Co(1)-N(1)	89.29(12)	91.86
N(3)#1-Co(1)-N(1)	178.55(12)	171.56
N(1)#1-Co(1)-N(1)	90.86(16)	92.05
O(2)-Cr(1)-O(3)#1	110.95(11)	106.17
O(2)-Cr(1)-O(3)	110.95(11)	110.24
O(3)#1-Cr(1)-O(3)	107.53(18)	112.81
O(2)-Cr(1)-O(1)	110.45(17)	109.66
O(3)#1-Cr(1)-O(1)	108.43(11)	109.66
O(3)-Cr(1)-O(1)	108.43(11)	113.18
Cr(1)-O(1)-Co(1)	134.14(19)	122.22
R		0.99
MAE [°]		3.99

Table S11. Experimental and theoretical (at the DFT/B3LYP/6-311+G(d,p)(H,N,O,Cl)/ LanL2DZ(Co,Cr) level of theory) bond lengths (in Å) of compound **2**.

Bond	Experimental	Theoretical
Co(1A)-O(1A)	1.899(3)	1.903
Co(1A)-N(1A)	1.944(4)	1.969
Co(1A)-N(2A)	1.950(4)	1.980
Co(1A)-N(3A)	1.950(4)	1.996
Co(1A)-N(4A)	1.962(4)	2.011
Co(1A)-N(5A)	1.967(4)	2.012
Co(1B)-O(1B)	1.917(3)	1.920
Co(1B)-N(2B)	1.946(4)	2.013
Co(1B)-N(3B)	1.949(4)	1.962
Co(1B)-N(1B)	1.959(4)	1.991
Co(1B)-N(4B)	1.962(4)	1.972
Co(1B)-N(5B)	1.969(4)	2.022
Cr(1A)-O(3A)	1.622(3)	1.600
Cr(1A)-O(2A)	1.647(3)	1.637
Cr(1A)-O(4A)	1.648(3)	1.656
Cr(1A)-O(1A)	1.692(3)	1.758
Cr(1B)-O(2B)	1.633(3)	1.594
Cr(1B)-O(3B)	1.635(3)	1.638
Cr(1B)-O(4B)	1.654(4)	1.667
Cr(1B)-O(1B)	1.697(3)	1.750
Cr(3)-O(12)	1.610(4)	1.604
Cr(3)-O(10)	1.626(5)	1.659
Cr(3)-O(9)	1.636(5)	1.672
Cr(3)-O(11)	1.672(4)	1.697
R		0.99
MAE [Å]		0.03

Table S12. Experimental and theoretical (at the DFT/B3LYP/6-311+G(d,p)(H,N,O,Cl)/LanL2DZ(Co,Cr) level of theory) bond angles (in °) of compound **2.**

Bond angle	Experimental	Theoretical
O(1A)-Co(1A)-N(1A)	177.68(15)	172.92
O(1A)-Co(1A)-N(2A)	88.55(15)	85.68
N(1A)-Co(1A)-N(2A)	90.47(17)	96.27
O(1A)-Co(1A)-N(3A)	89.36(16)	86.28
N(1A)-Co(1A)-N(3A)	92.70(17)	89.09
N(2A)-Co(1A)-N(3A)	87.75(17)	89.18
O(1A)-Co(1A)-N(4A)	89.99(16)	94.39
N(1A)-Co(1A)-N(4A)	91.00(18)	91.81
N(2A)-Co(1A)-N(4A)	178.50(18)	177.38
N(3A)-Co(1A)-N(4A)	91.87(17)	90.69
O(1A)-Co(1A)-N(5A)	87.68(16)	83.50
N(1A)-Co(1A)-N(5A)	90.25(17)	90.74
N(2A)-Co(1A)-N(5A)	91.48(16)	92.45
N(3A)-Co(1A)-N(5A)	176.96(17)	171.92
N(4A)-Co(1A)-N(5A)	88.83(17)	89.71
O(1B)-Co(1B)-N(2B)	88.84(16)	87.13
O(1B)-Co(1B)-N(3B)	91.25(16)	93.61
N(2B)-Co(1B)-N(3B)	87.32(18)	88.22
O(1B)-Co(1B)-N(1B)	179.41(17)	175.81
N(2B)-Co(1B)-N(1B)	91.65(18)	96.09
N(3B)-Co(1B)-N(1B)	89.09(18)	89.17
O(1B)-Co(1B)-N(4B)	89.69(16)	85.11
N(2B)-Co(1B)-N(4B)	178.52(18)	172.24
N(3B)-Co(1B)-N(4B)	92.87(18)	92.17
N(1B)-Co(1B)-N(4B)	89.82(19)	91.66
O(1B)-Co(1B)-N(5B)	88.16(16)	81.03
N(2B)-Co(1B)-N(5B)	90.42(18)	90.89
N(3B)-Co(1B)-N(5B)	177.68(18)	174.60
N(1B)-Co(1B)-N(5B)	91.52(18)	96.21
N(4B)-Co(1B)-N(5B)	89.37(18)	88.00
O(3A)-Cr(1A)-O(2A)	109.79(17)	113.62
O(3A)-Cr(1A)-O(4A)	108.22(17)	112.95
O(2A)-Cr(1A)-O(4A)	110.37(17)	109.31
O(3A)-Cr(1A)-O(1A)	110.36(17)	110.81
O(2A)-Cr(1A)-O(1A)	108.08(16)	103.51
O(4A)-Cr(1A)-O(1A)	110.03(16)	105.96
O(2B)-Cr(1B)-O(3B)	109.46(18)	112.33
O(2B)-Cr(1B)-O(4B)	111.16(18)	109.35
O(3B)-Cr(1B)-O(4B)	106.99(18)	112.78

O(2B)-Cr(1B)-O(1B)	108.61(17)	107.72
O(3B)-Cr(1B)-O(1B)	110.94(17)	110.25
O(4B)-Cr(1B)-O(1B)	109.69(17)	103.97
O(12)-Cr(3)-O(10)	110.0(3)	110.22
O(12)-Cr(3)-O(9)	110.9(3)	107.38
O(10)-Cr(3)-O(9)	110.9(3)	114.29
O(12)-Cr(3)-O(11)	107.2(2)	104.68
O(10)-Cr(3)-O(11)	110.5(3)	110.85
O(9)-Cr(3)-O(11)	107.3(3)	108.93
Cr(1A)-O(1A)-Co(1A)	138.19(18)	120.06
Cr(1B)-O(1B)-Co(1B)	131.67(19)	123.52
R		0.99
MAE [°]		3.17

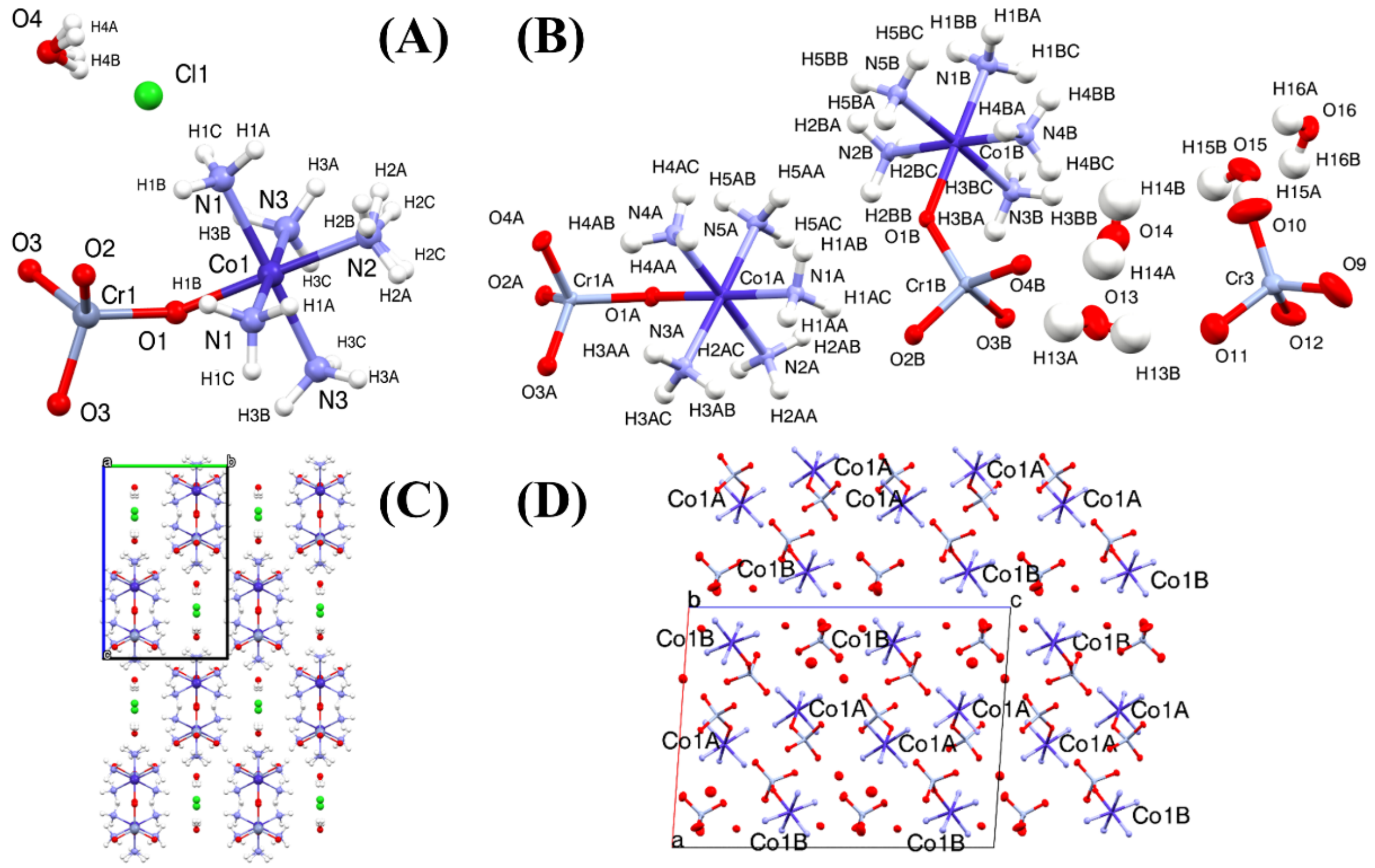

Figure S2. The asymmetric unit of compounds **1(a)** and **2(b),** and the packing viewed along the crystallographic *a* axis of compound **1(c)** and viewed along the crystallographic *b* axis of compound **2(d)**.

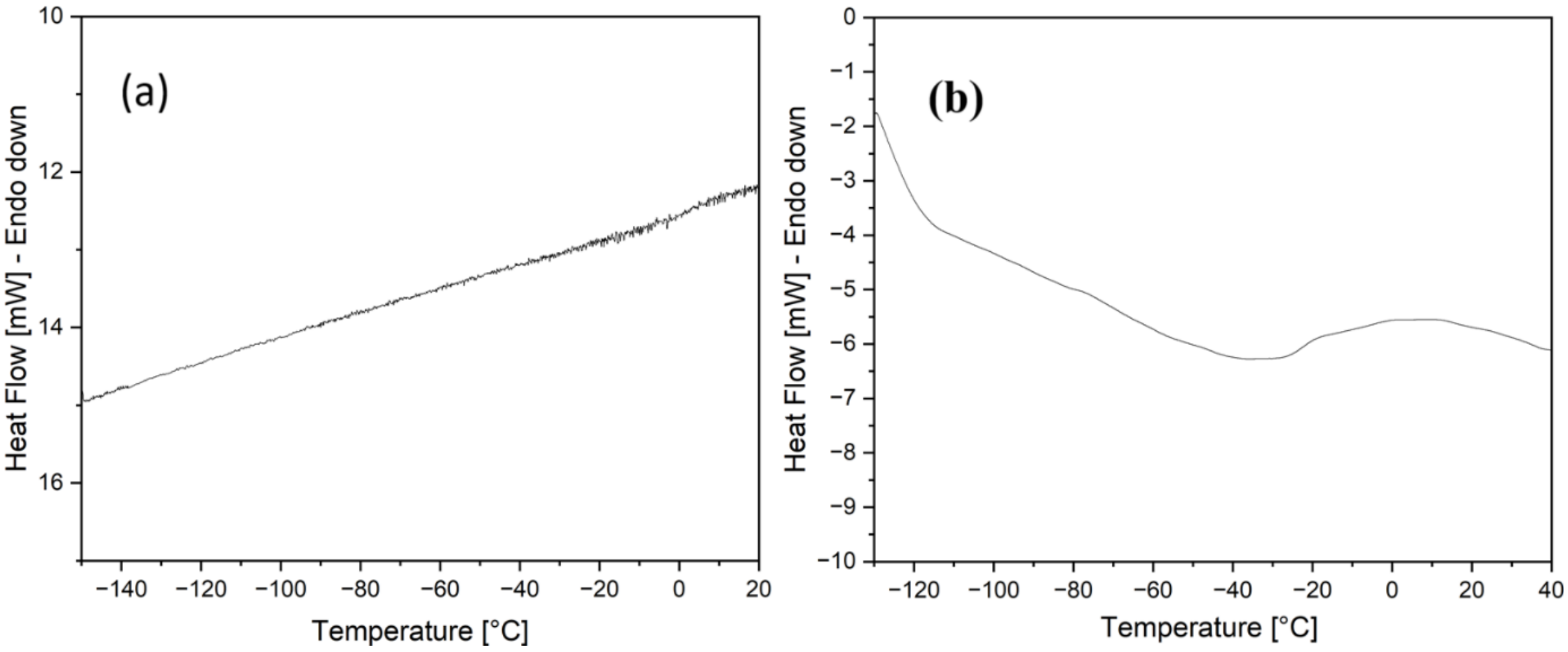

Figure S3. The cryo-DSC curves of compounds **1 (a)** and **2 (b)**.

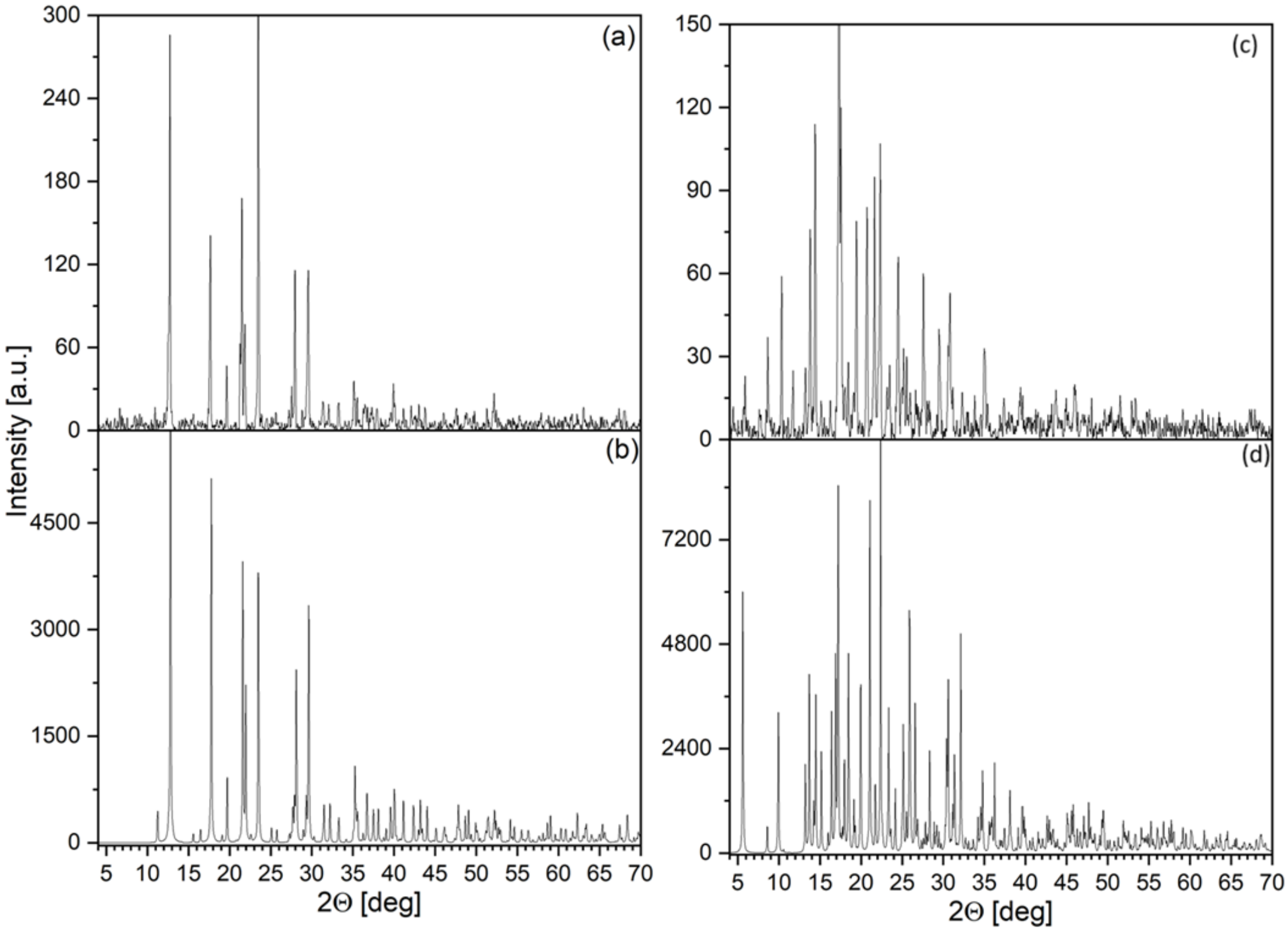

Figure S4. The measured and the calculated (from the SCXRD) PXRD of compounds **1 ((a)** and (**b),** respectively**)** and **2 ((c)** and (**d),** respectively**)**.

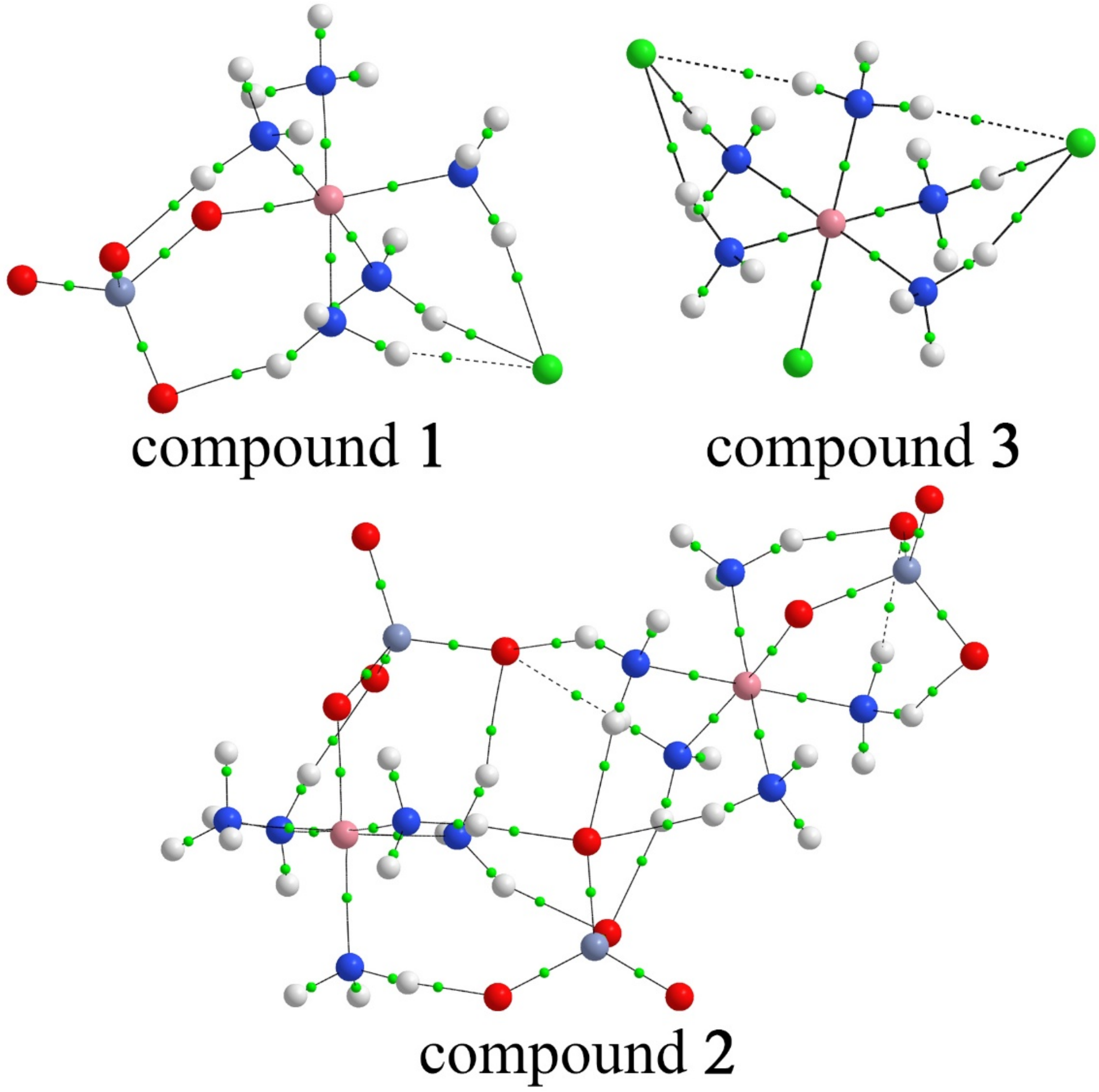

Figure S5. The Bond Critical Points in the optimized structure of compounds **1**-**3** (at the DFT/B3LYP/6-311+G(d,p)(H,N,O,Cl)/LanL2DZ(Co,Cr) level of theory).

Table S13. The calculated Bond Critical Point (BCP) properties at the DFT/B3LYP/6-311+G(d,p)(H,N,O,Cl)/LanL2DZ(Co,Cr) level of theory of complexes **1**-**3**.

Bond	ρ(r) [a.u.]	∇^2ρ(r) [a.u.]	*G*(r) [kJ mol^{-1}]	*V*(r) [kJ mol^{-1}]	*H*(r) [kJ mol^{-1}]	-*G*(r)/V(r)	E_{bond} [kJ mol^{-1}]
Compound **1**, $[Co(NH_3)_5CrO_4]Cl$							
Co−N	0.089	0.382	303.7	-357.0	-53.3	0.9	-178.5
Cr−O	0.265	1.115	1134.0	-1535.9	-401.9	0.7	-767.9
Cr−O(Co)	0.160	0.648	590.9	-756.4	-165.5	0.8	-378.2
Co−O	0.103	0.485	377.7	-437.2	-59.5	0.9	-218.6
NH···Cl	0.039	0.075	62.8	-76.2	-13.5	0.8	-38.1
Compound **2**, $[Co(NH_3)_5CrO_4]_2CrO_4$							
Co−N	0.089	0.400	315.8	-369.1	-53.3	0.9	-184.6
Cr−O_{coord}	0.244	1.00	1005.0	-1352.4	-347.3	0.7	-676.2
Cr−O_{anion}	0.242	0.989	991.7	-1334.3	-342.7	0.7	-667.2
Cr−O(Co)	0.164	0.650	607.9	-788.8	-181.0	0.8	-394.4
Co−O	0.090	0.472	350.4	-390.9	-40.5	0.9	-195.4
NH···O	0.059	0.167	134.8	-160.0	-25.2	0.8	-80.0
$[Co(NH_3)_5Cl]Cl_2$							
Co−N	0.088	0.393	308.5	-358.9	-50.5	0.9	-179.5
Co−Cl	0.056	0.187	139.9	-157.1	-17.3	0.9	-78.6
NH···Cl	0.032	0.081	57.6	-62.0	-4.4	0.9	-31.0

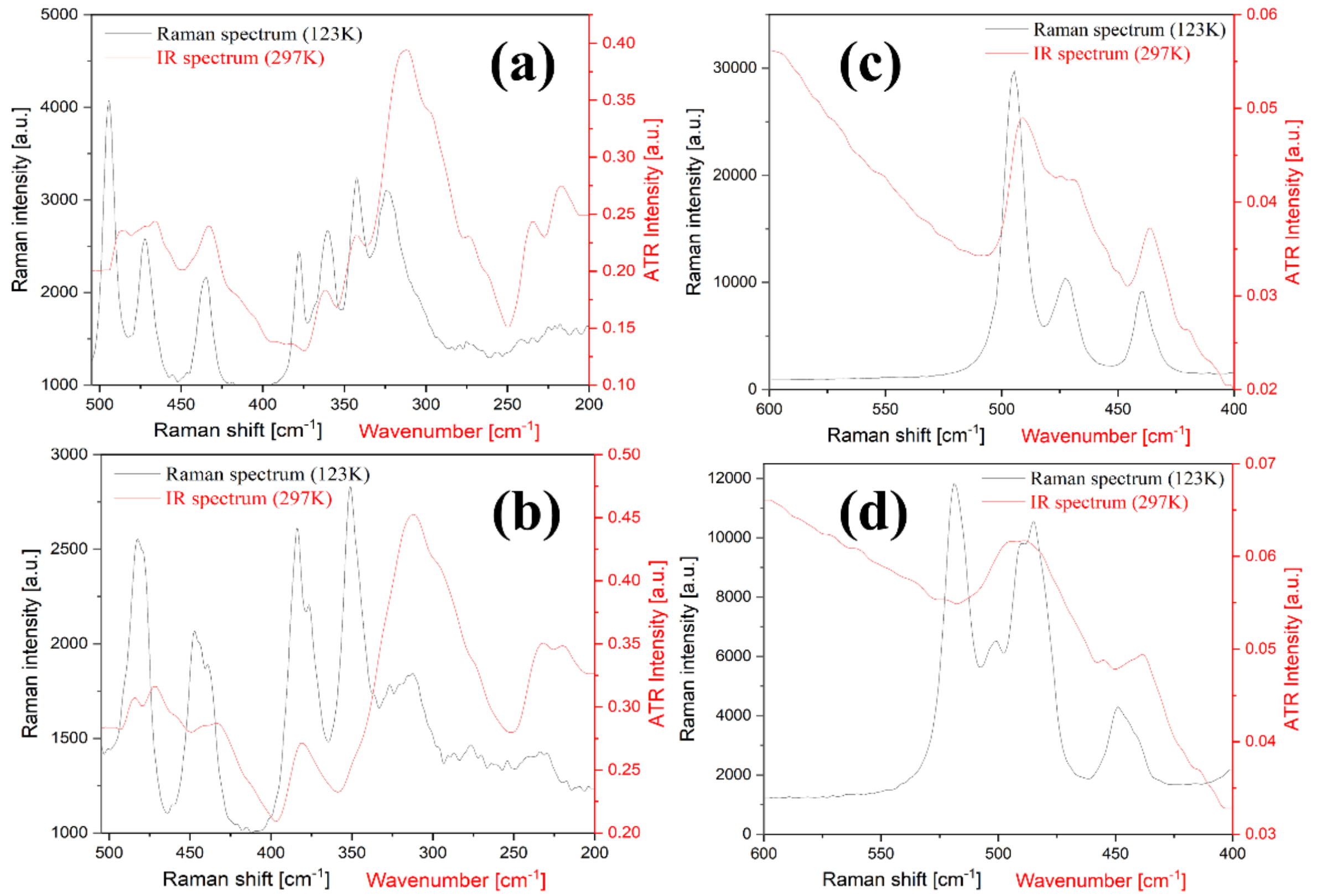

Figure S6. The region of anion and [CoN_5O] core vibrational modes of compound **1 (a,b)** and compound **2 (c,d)**.

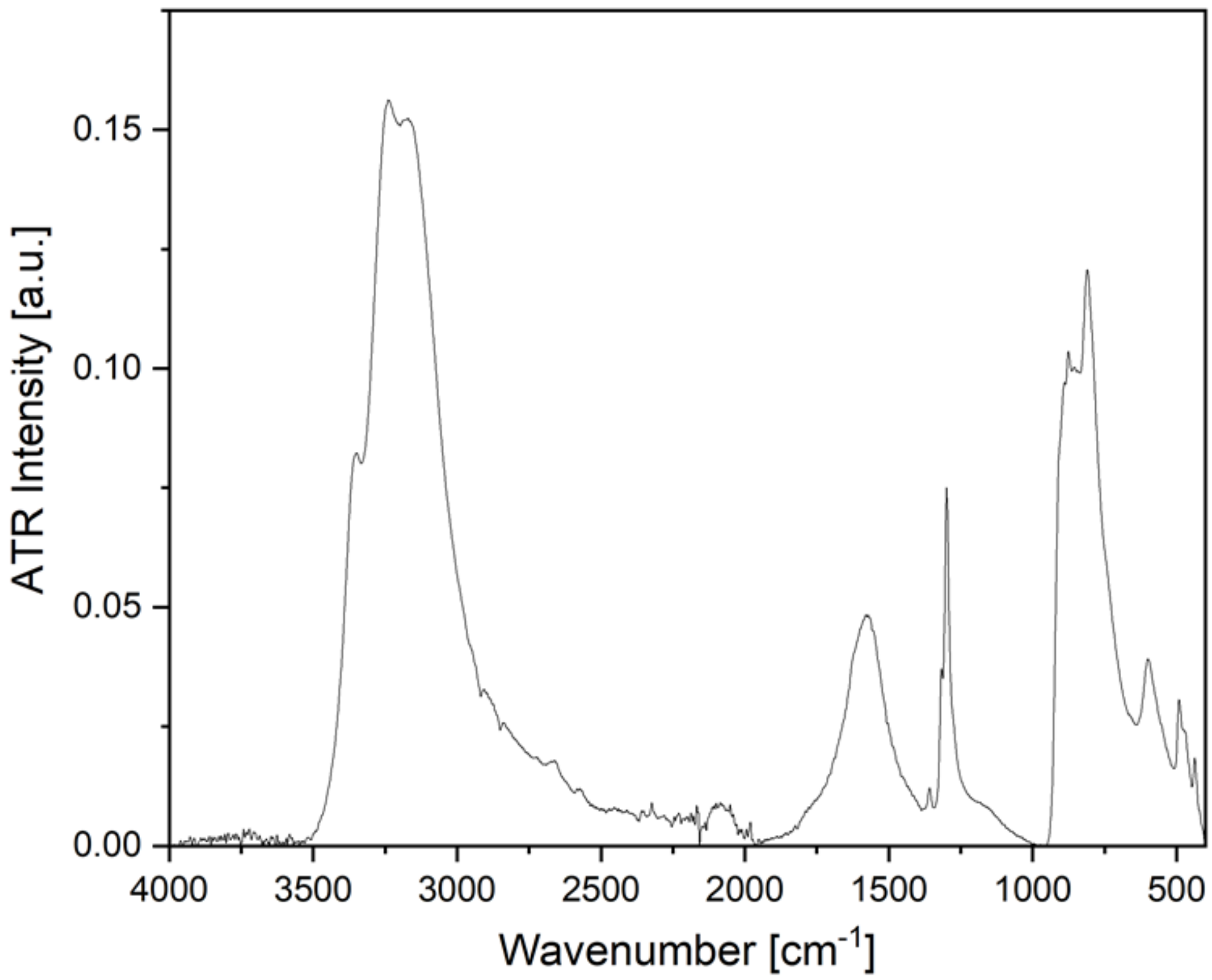

Figure S7. The analytic range IR spectra of $[Co(NH_3)_5CrO_4]Cl\cdot H_2O$.

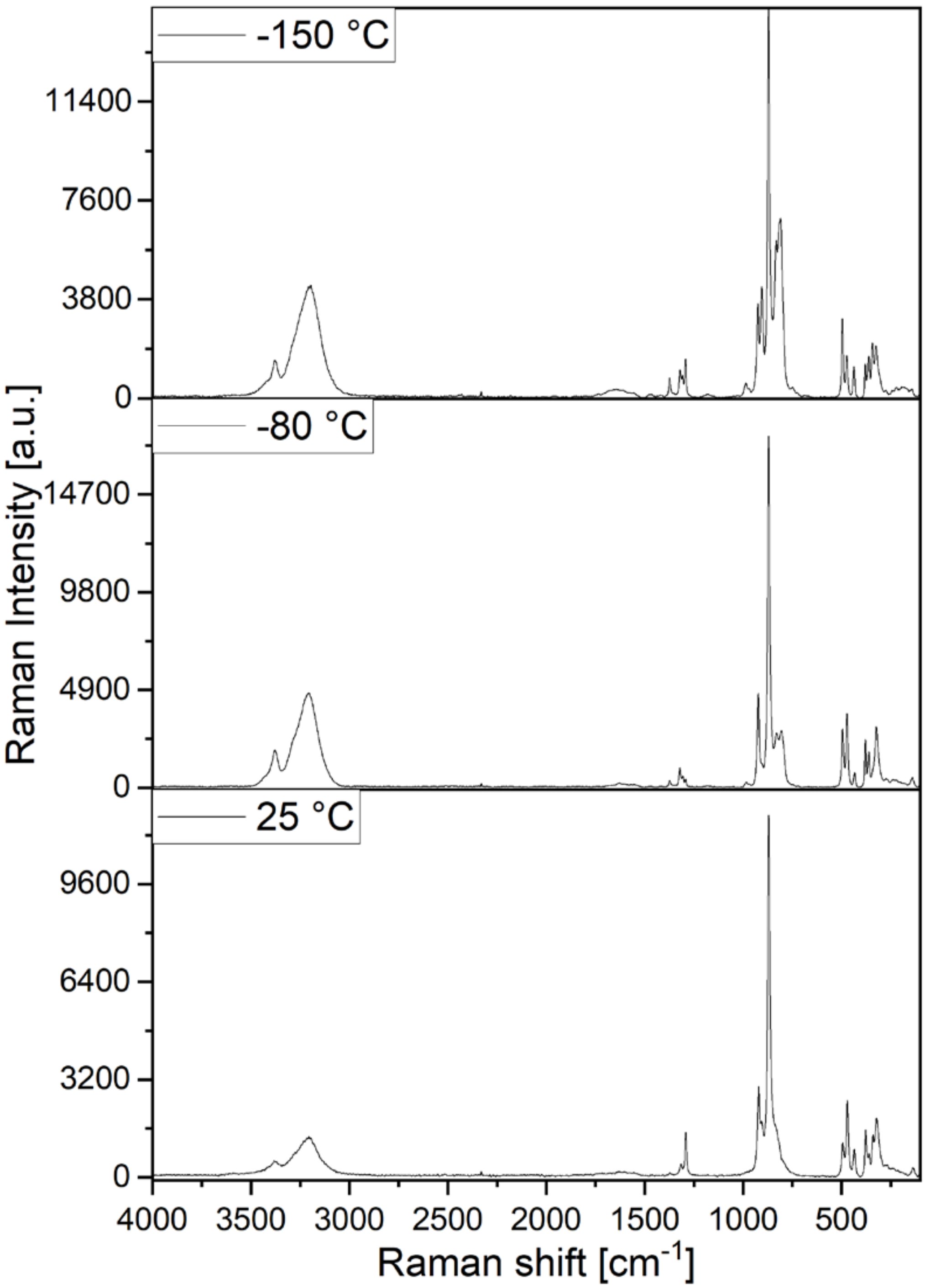

Figure S8. The Raman spectra of $[Co(NH_3)_5CrO_4]Cl{\cdot}H_2O$ measured at different temperatures 532 nm laser.

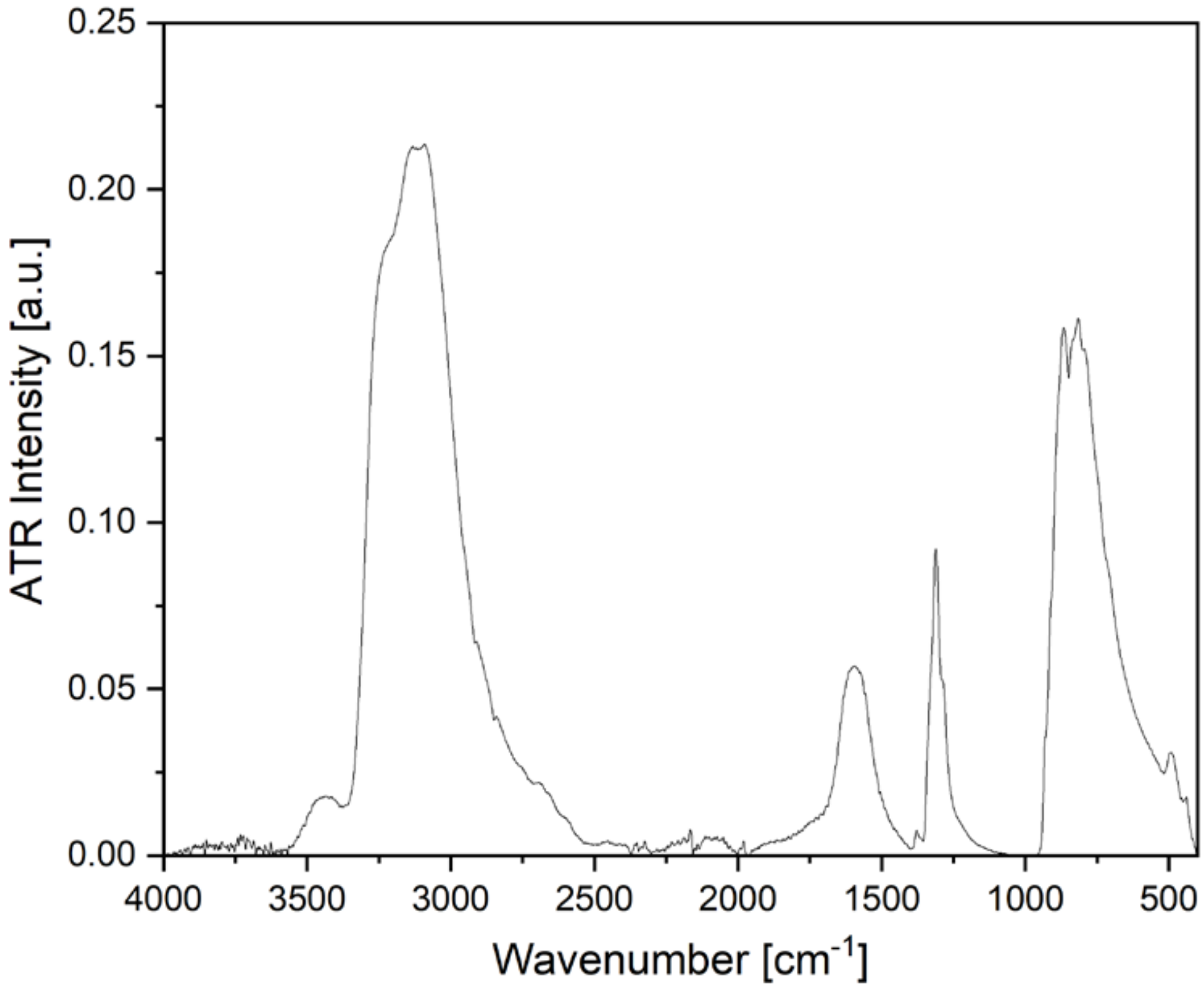

Figure S9. The analytic range IR spectra of $[Co(NH_3)_5CrO_4]_2CrO_4·4H_2O$.

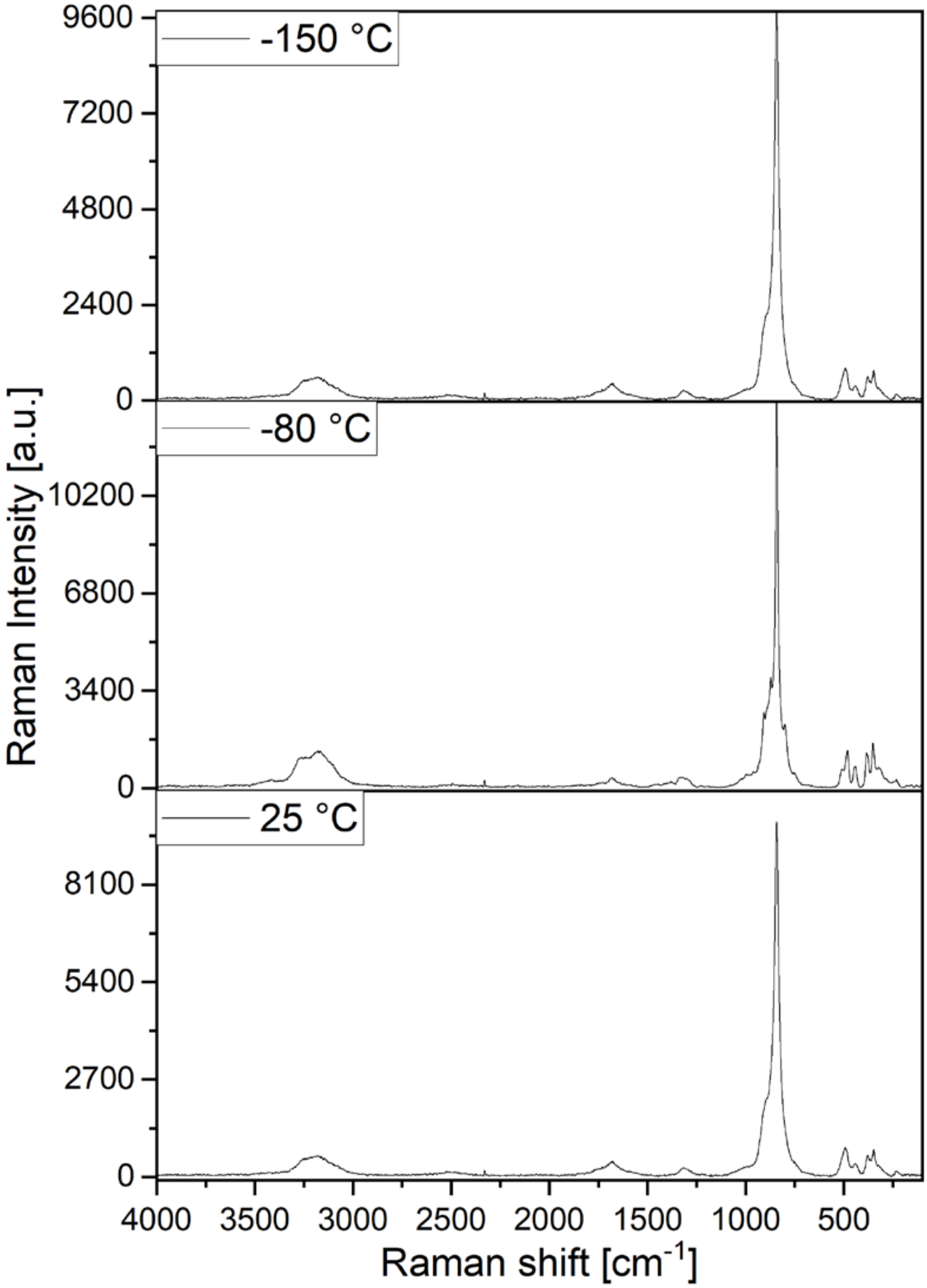

Figure S10. The Raman spectra of $[Co(NH_3)_5CrO_4]_2CrO_4{\cdot}4H_2O$ masured at different temperatures with 532 nm laser.

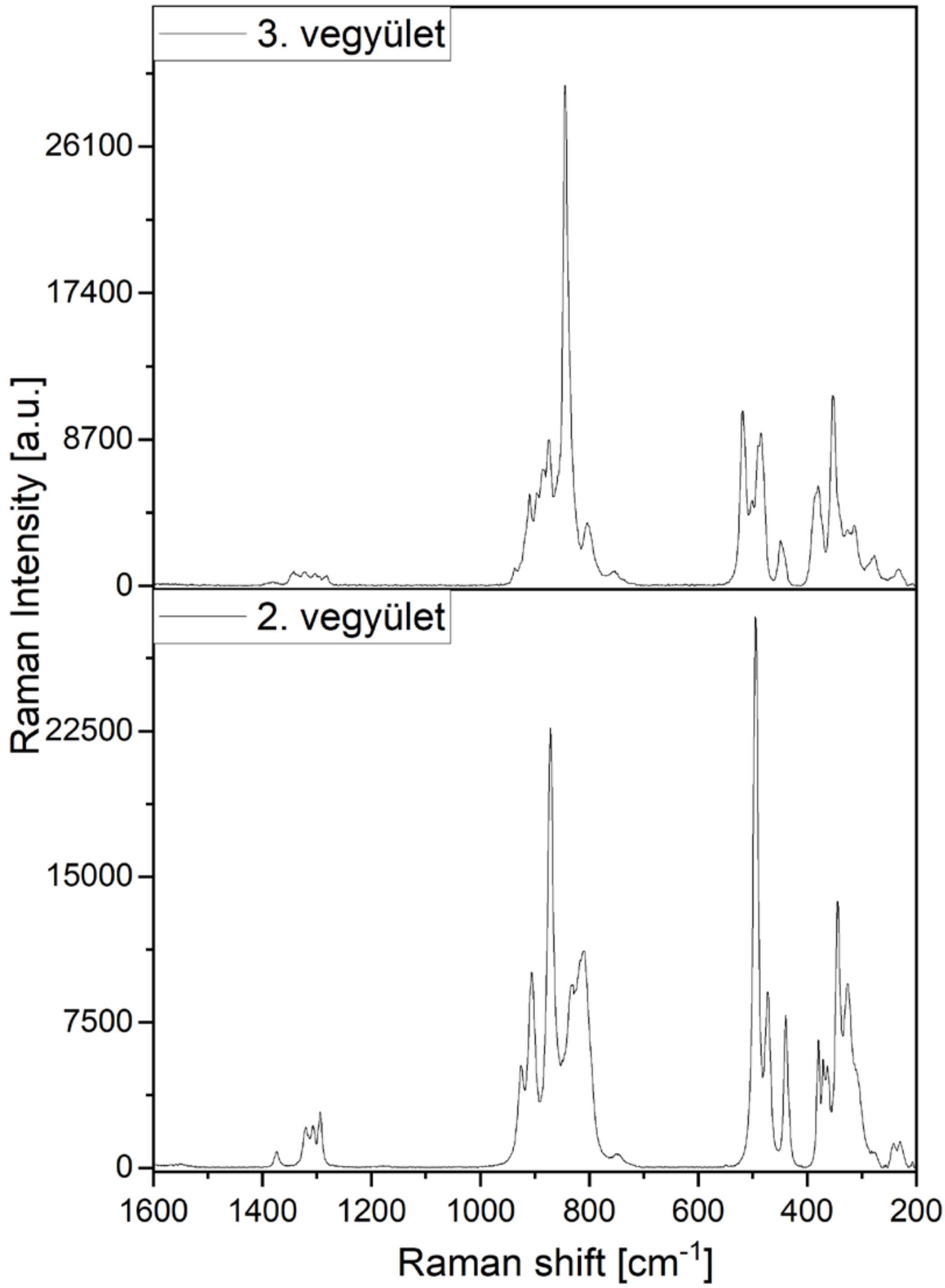

Figure S11. The Raman spectra of $[Co(NH_3)_5CrO_4]Cl{\cdot}H_2O$ **(a)** and $[Co(NH_3)_5CrO_4]_2CrO_4{\cdot}4H_2O$ **(b)** measured at -150 °C with 785 nm laser.

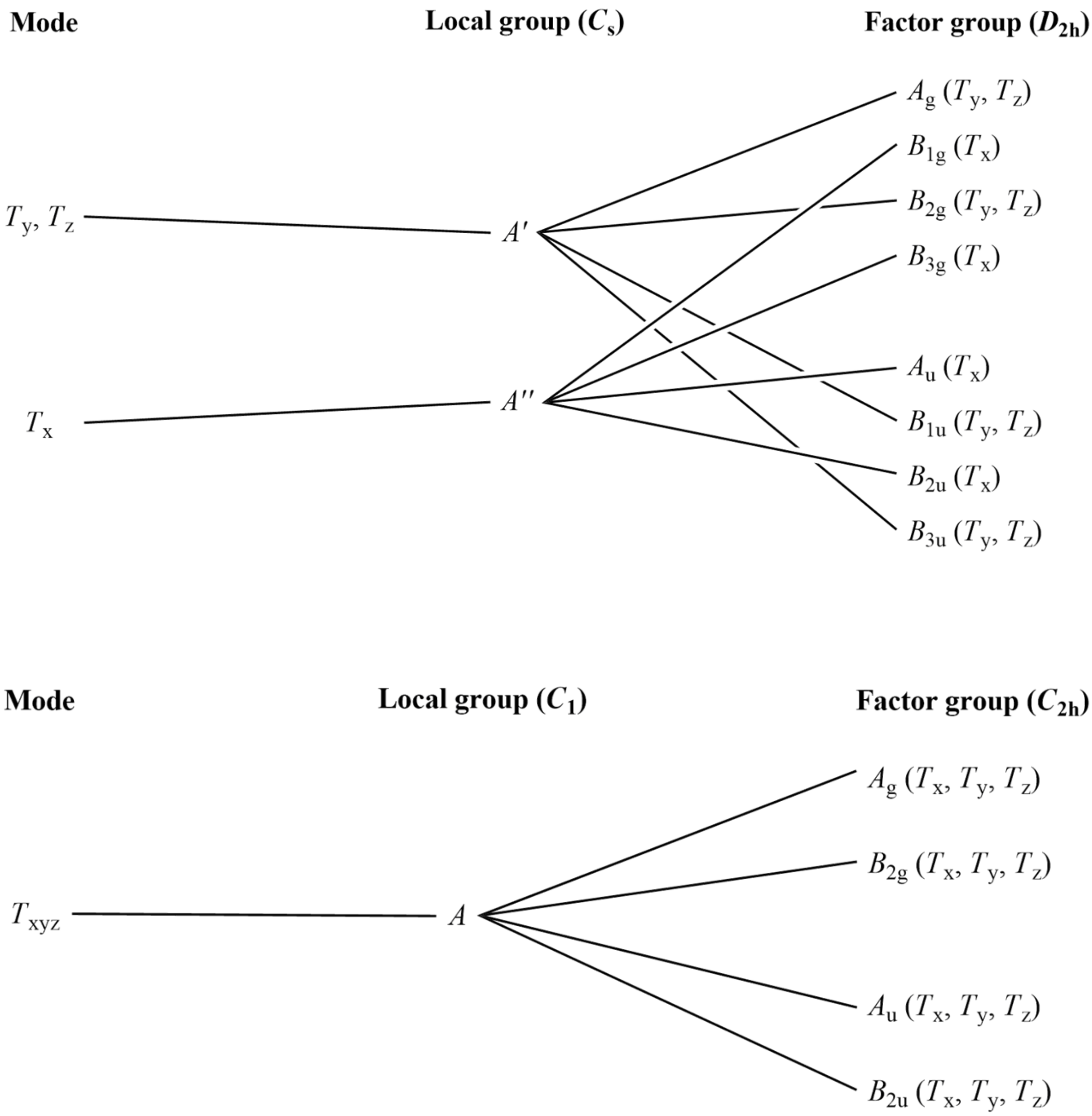

Figure S12. The hindered translations of the monoatomic ions: Co^{3+} and Cl^{-} for compound **1**(a) and Co^{3+} for compound **2**(b).

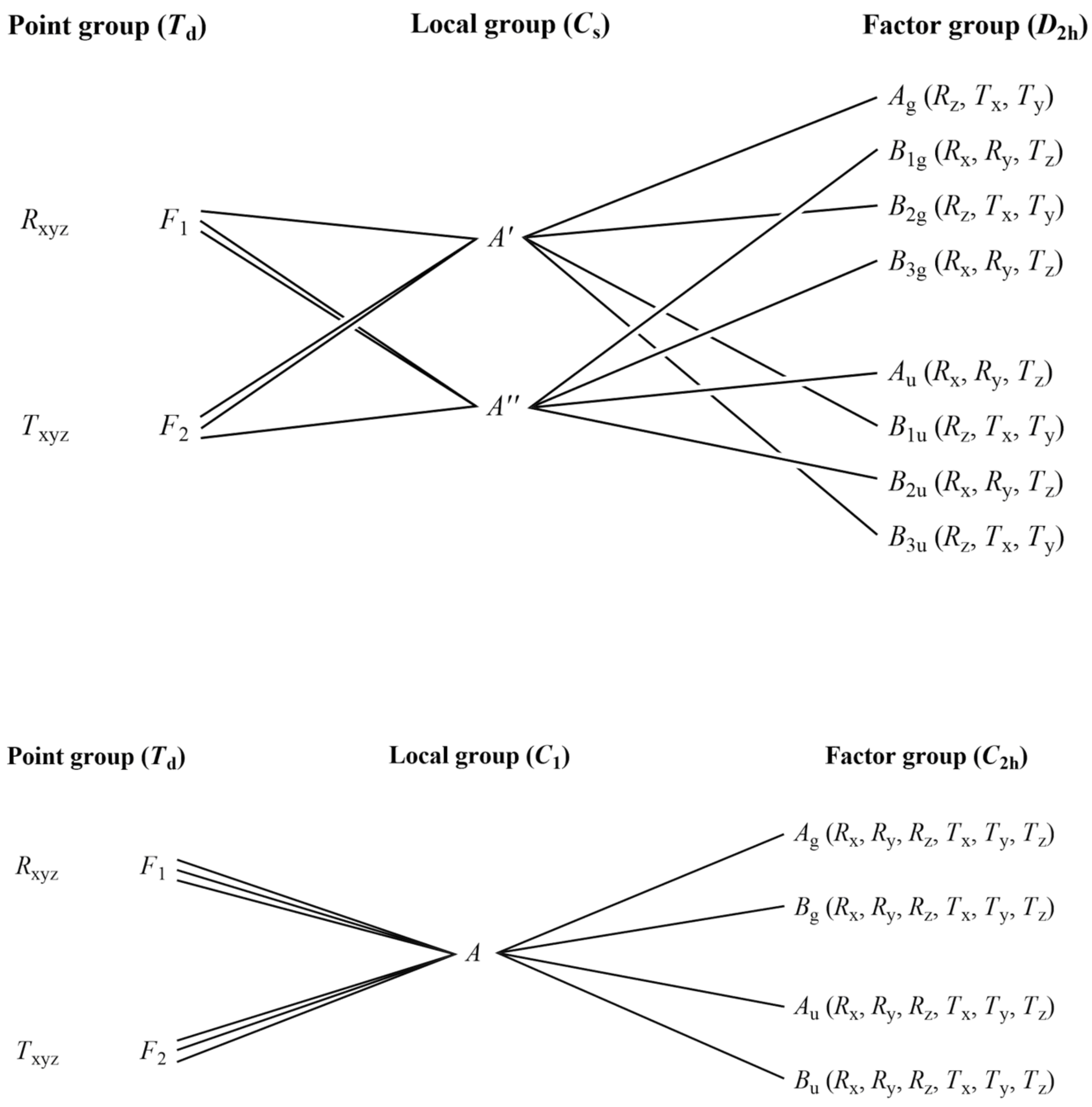

Figure S13 The external vibrations of the chromate ion in (a) compound **1** and **(b) 2**.

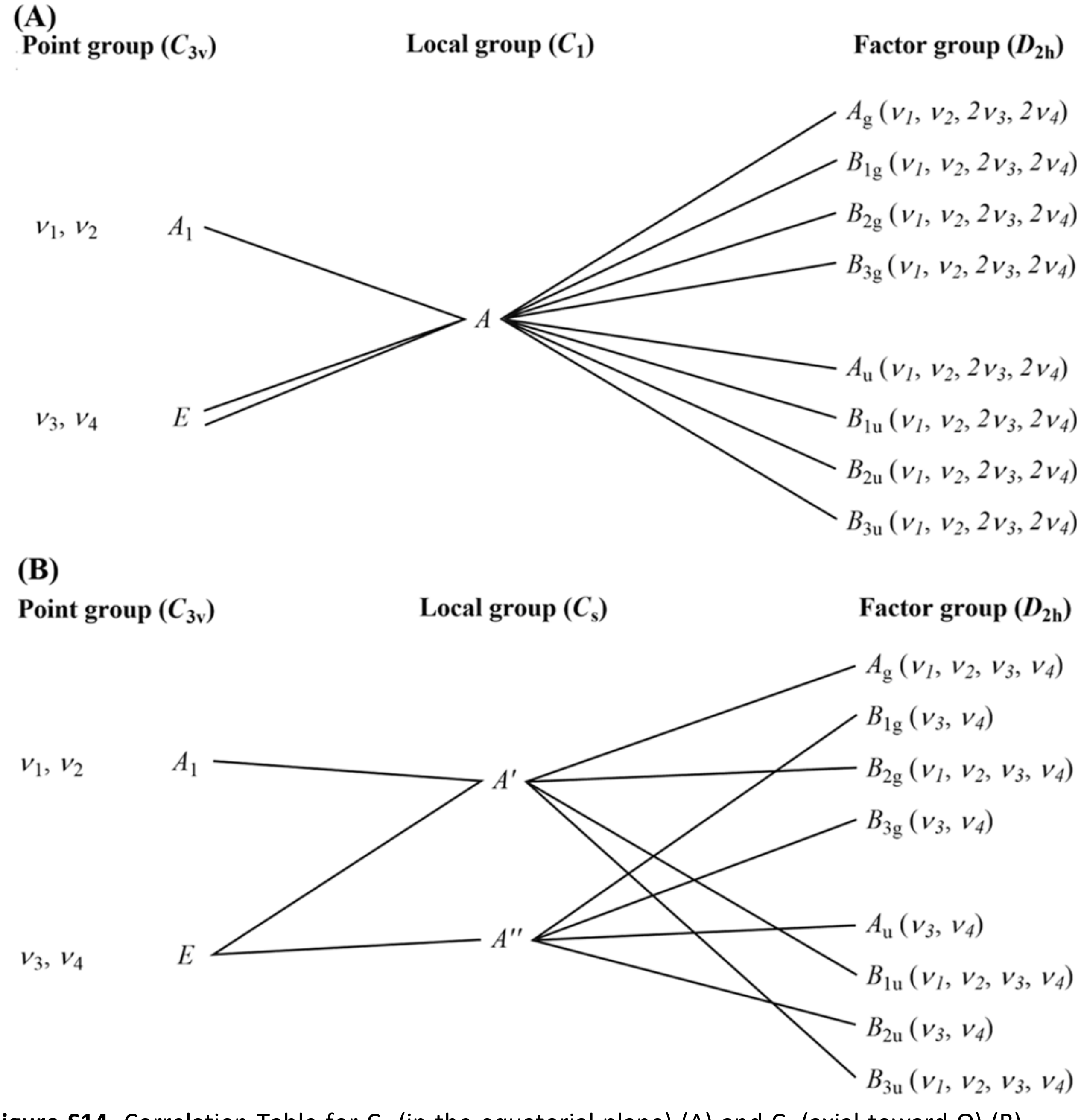

Figure S14. Correlation Table for C_1 (in the equatorial plane) (A) and C_s (axial toward O) (B) ammonia ligands in compound **1**.

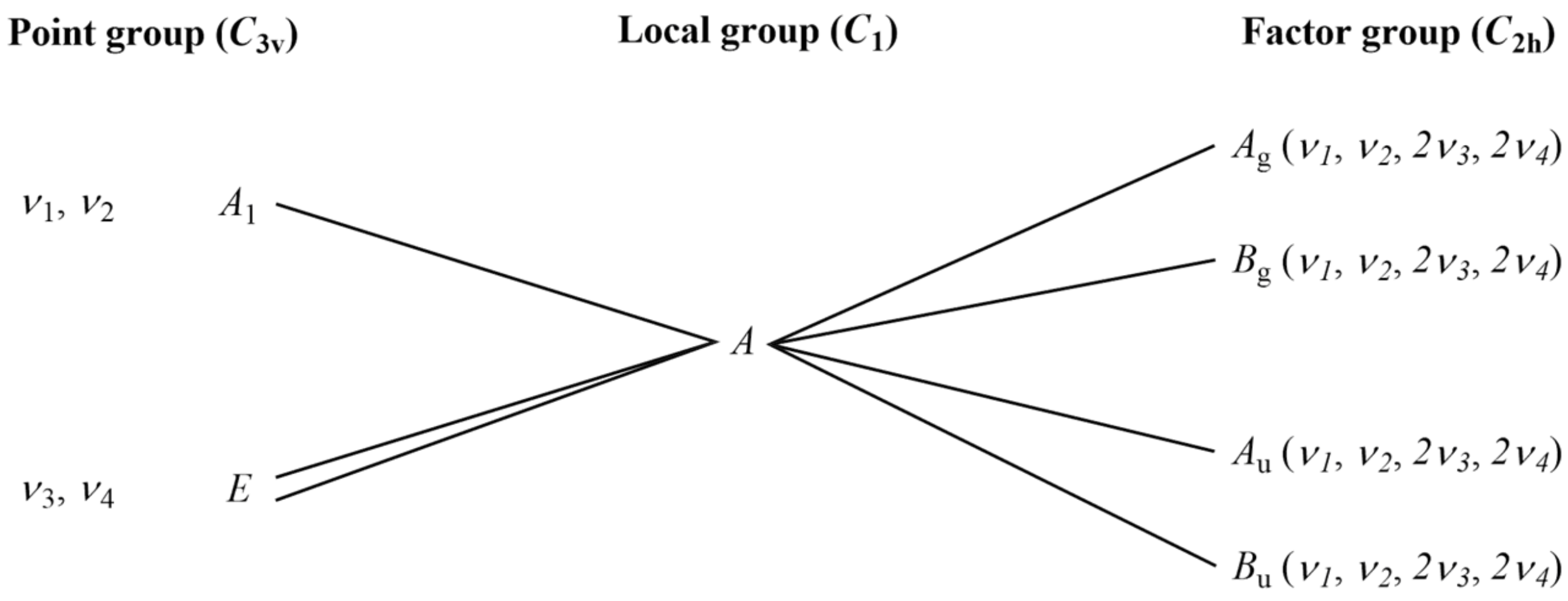

Figure S15. The correlation table for ammonia molecules in compound **2**.

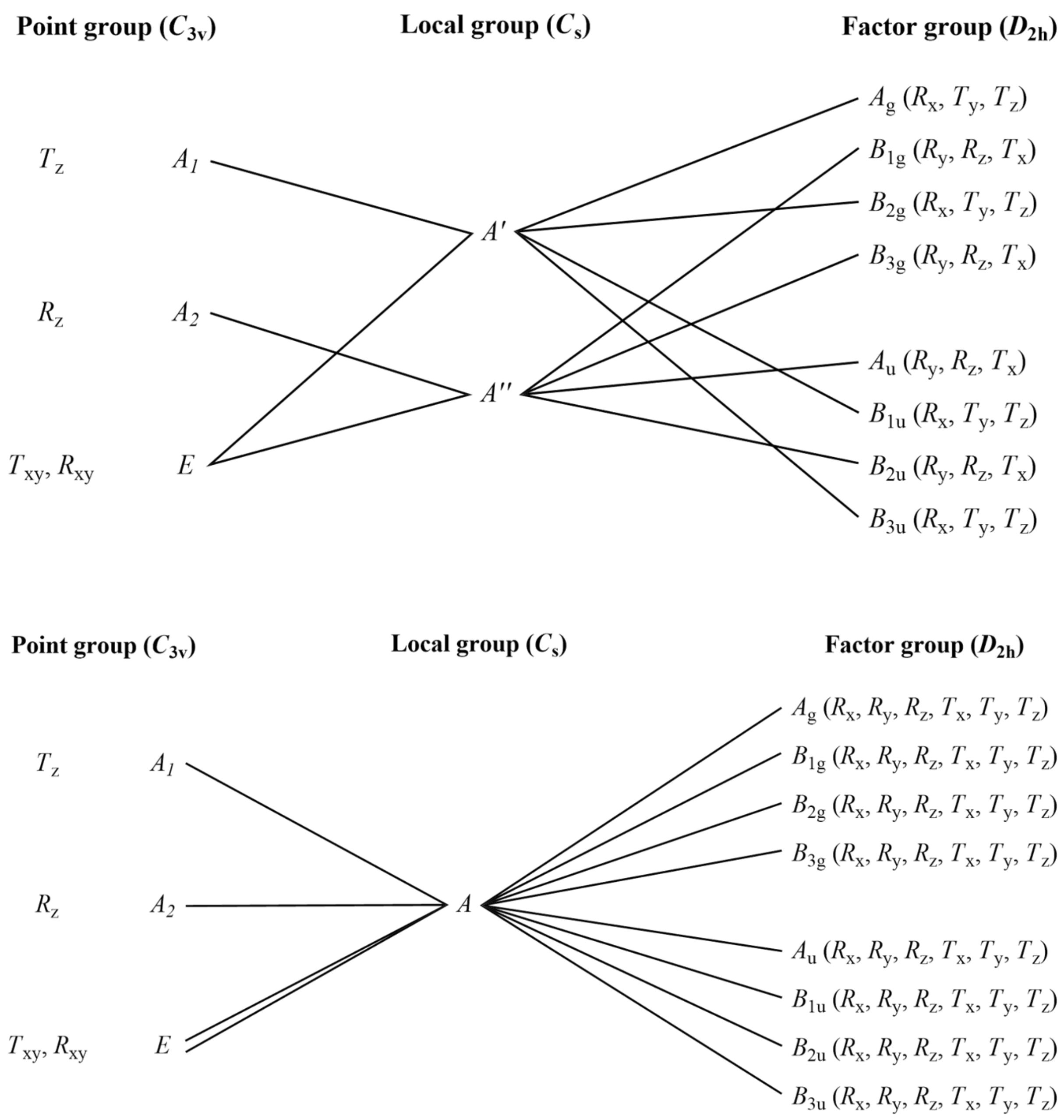

Figure S16. The external vibrations of the ammonia ligands in compound **1**

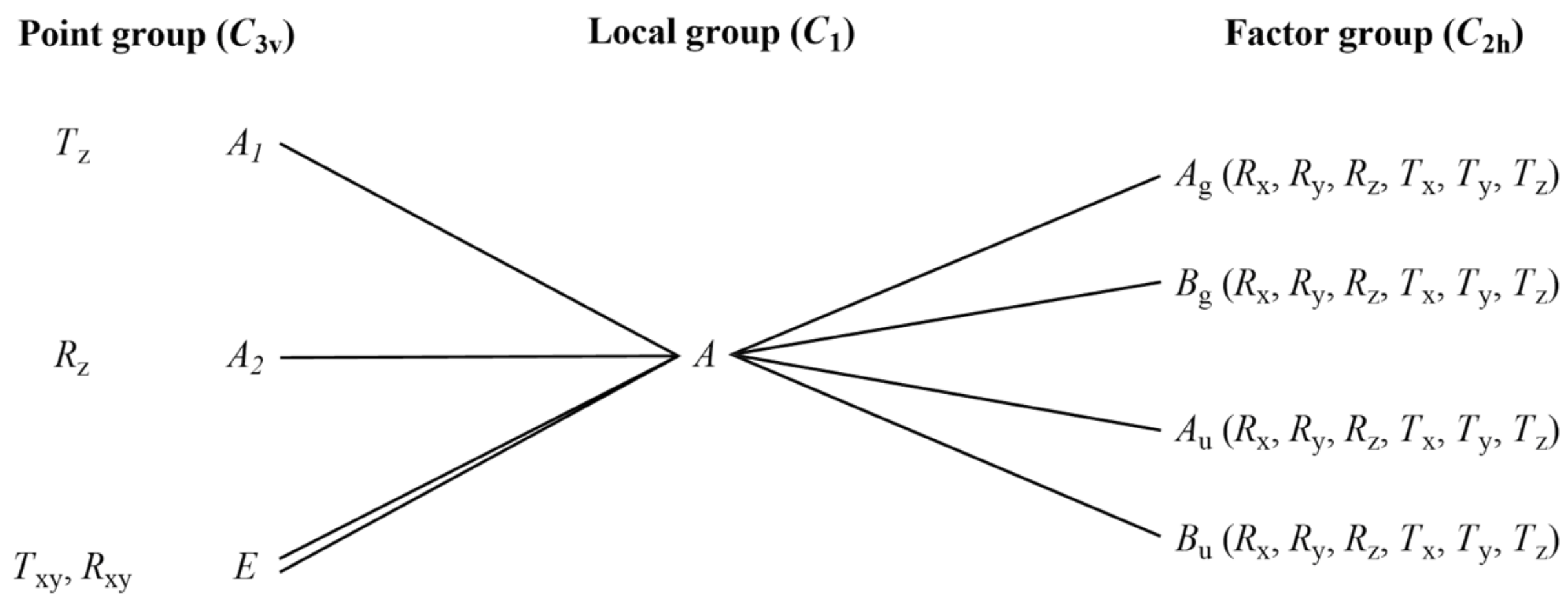

Figure S17. The external vibrations of the ammonia ligands in compound **2.**

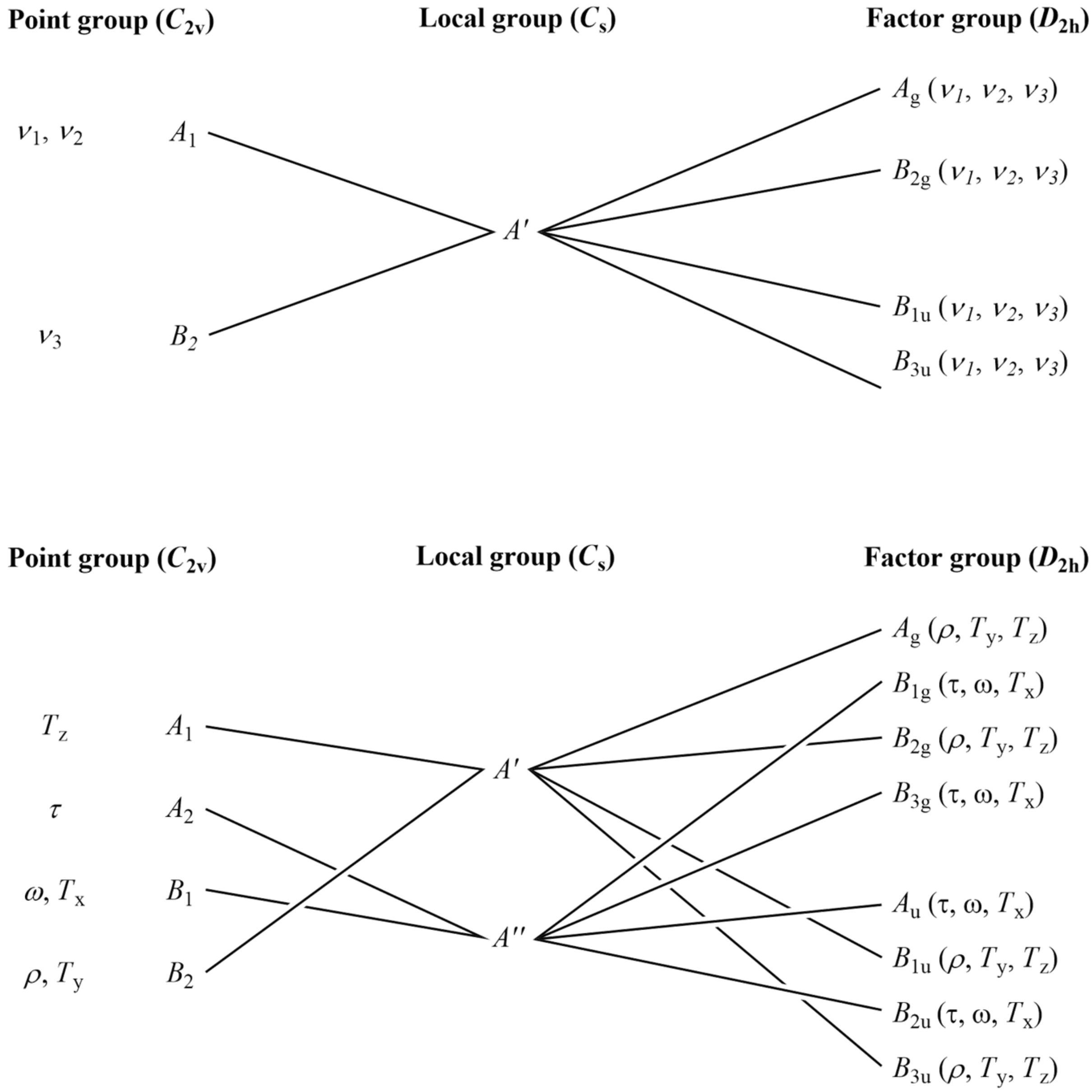

Figure S18. The internal (a) and external (b) vibrational modes of water molecules in compound **1**

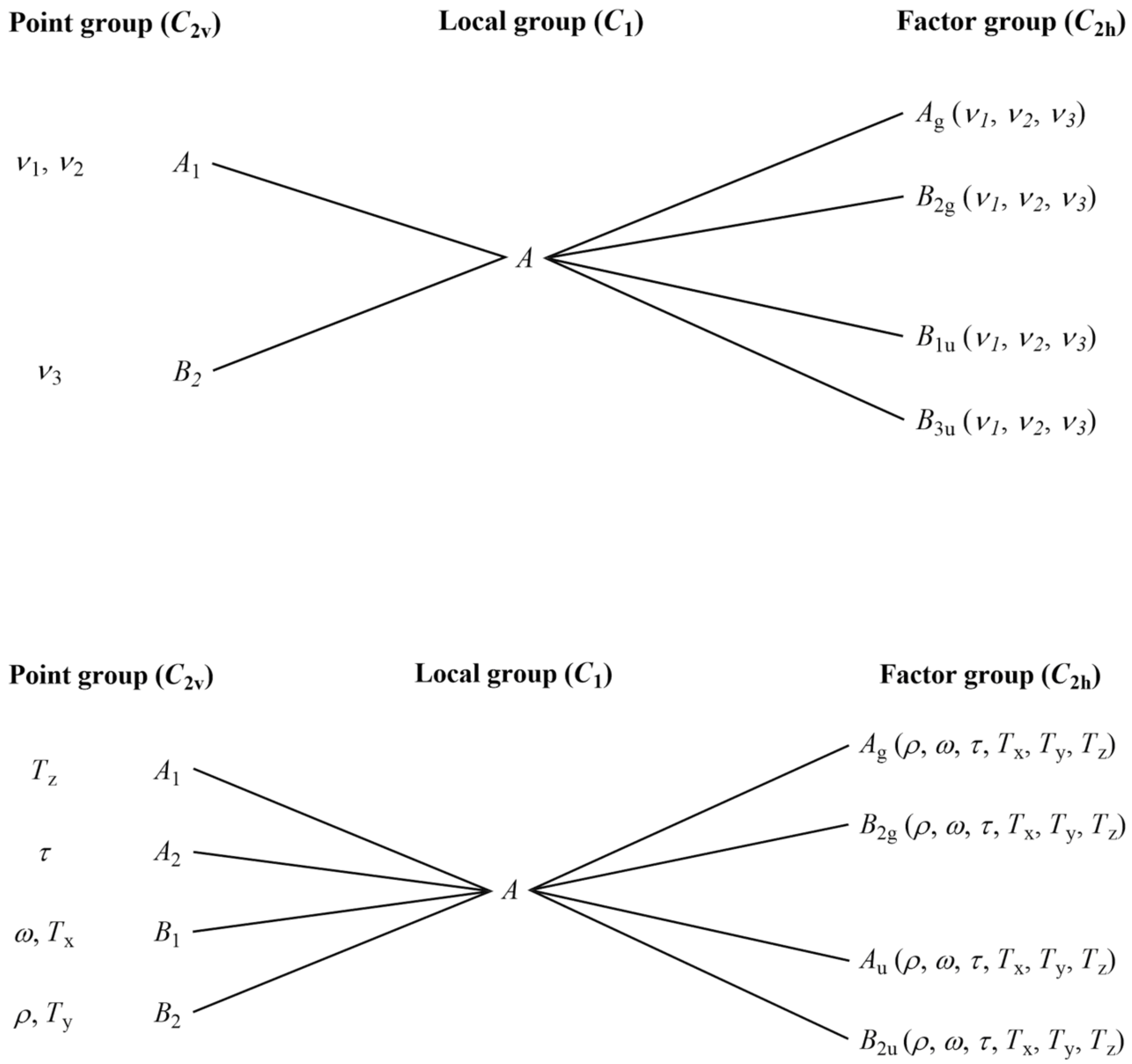

Figure S19. The internal (a) and external (b) vibrational modes of water molecules in compound **2.**

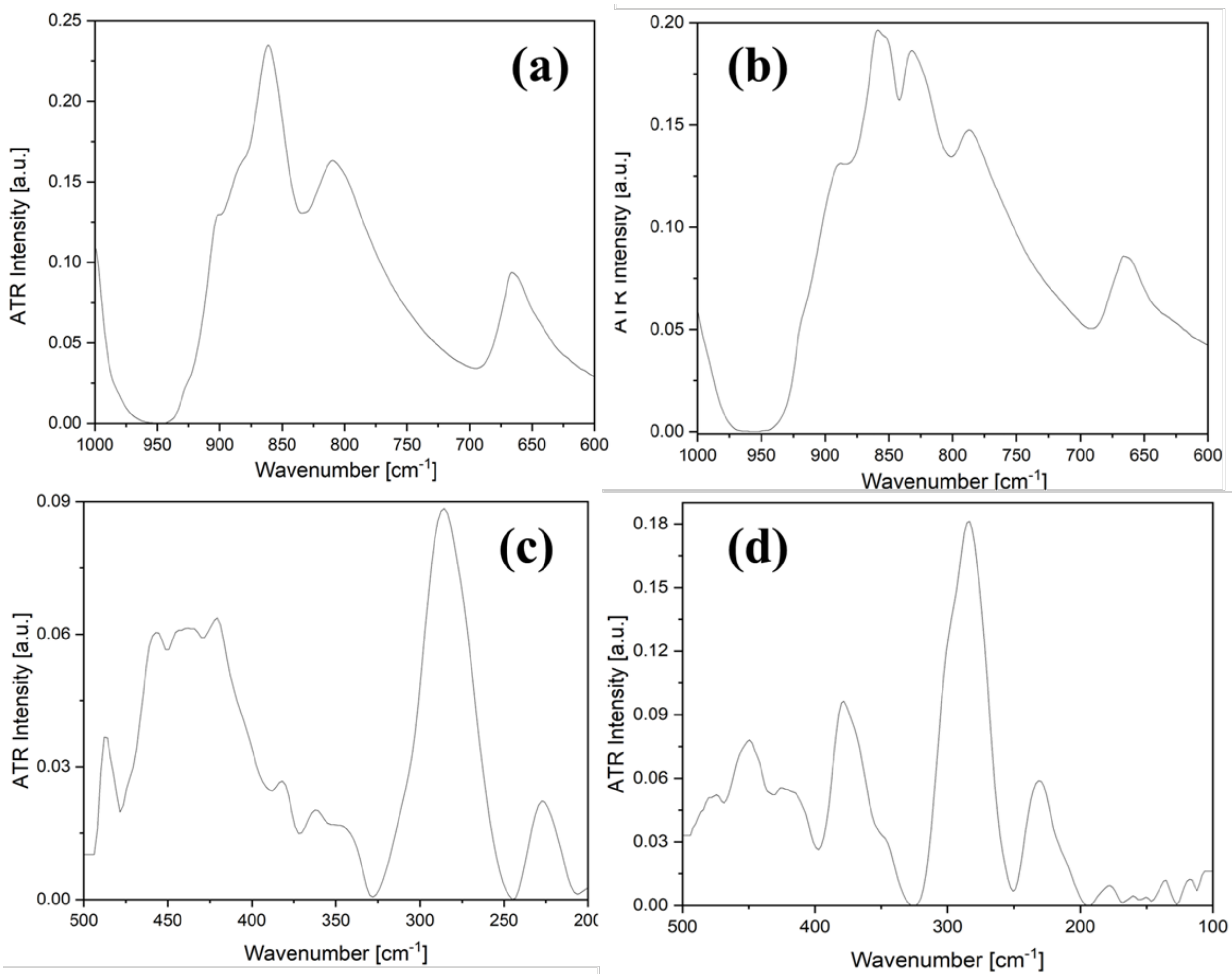

Figure S20. The analytical and far-range IR spectra of compounds **1-D (a,c)** and **2-D (b,d)**.

Table S14. Assignments of normal modes (cm^{-1}) in the IR and Raman spectra of compounds **1** and **2.**

Normal mode	Compound **1**		**1-D**	Compound **2**		**2-D**
	IR	LT Raman,	IR	IR	LT Raman	IR
ν_1(Cr-O) (ν_s)	889<br>875	871	861	833 (NC)<br>865 (C)	845 (NC)<br>874 (C)	832 (NC)<br>858 (C)
ν_2	343<br>314	342<br>324<br>313	347<br>311sh	312<br>296	326<br>314	348sh<br>311sh
ν_3(Cr-O) ν_{as}	909	925<br>905	902sh	911sh (NC)<br>930 (C)	885 (NC)<br>896 (NC)<br>910 (NC)<br>919 (C)<br>926 (C)<br>937 (C)	888 (NC)<br>919sh (C)
ν_4(as)	378<br>360	378<br>370sh<br>360	383<br>361	380<br>375sh<br>350	384<br>376<br>349sh	378<br>368sh

C-coordinated chromate (inner sphere); NC-non-coordinated chromate (outer sphere)

Text S3. The vibrational modes of [CoN_5O] skeleton and Co-O-Cr linkage in compounds 1 and 2.

The [CoN_5O] core vibrational modes might belong to Co-N and Co-O modes, and due to the coordinated chromate ion, it is obvious that the stretching and bending modes of the Co-O-Cr bridge should also appear. Coomber[5] identified three unassigned bands of compound **1**, at around 818, 601, and 215 cm^{-1}, which did not or hardly change their positions on deuteration, which supports the assumption that they do not belong to H-containing (NH_3 or H_2O) species. In comparison with M-O-M' bridged systems (M and M' are first-row transition metals) peak positions[6], two band positions can be assigned to the stretching modes of a mixed metal Co-O-Cr linkage in the IR spectra of compound **1,** at 807 cm^{-1} with high intensity, and at 595 cm^{-1} with medium intensity. This suggests that the band at 807 cm^{-1} is the antisymmetric mode, whereas the other at 595 cm^{-1} is the symmetric stretching mode of the Co-O-Cr linkage. The Raman band intensity at 808 cm^{-1} in the Raman spectrum of compound **1** is lower than its IR counterpart, as expected for antisymmetric stretching modes. The other band expected around 600 cm^{-1} did not appear in the Raman spectra –this band might be practically Raman inactive – because Co and Cr are different atoms in the Co-O-Cr linkage; thus the symmetric stretch may not produce a significant overall polarizability change. The antisymmetric Co-O-Cr bridge bands appeared at 793 and 787 cm^{-1} in the IR spectra of compounds **2** and **2-D**, respectively. No symmetric Co-O-Cr band was observed in the IR spectra of compounds **2** and **2-D**, and it was not present in the Raman spectrum of compound **2** either. The antisymmetric mode in the Raman spectrum of compound **2** was observed as a weak band at 804 cm^{-1} (Figs. 4 and S20). To assign the third unknown band found by Coomber[5] at 215 cm^{-1}, a detailed theoretical evaluation of the CoN_5O octahedral skeleton normal modes was performed. CoN_5O octahedra belong to the C_{4v} point group, and their 11 normal vibrations are active in Raman; among the $4A_1$, $2B_1$, B_2, and 4E modes, only A_1 and E are infrared-active. The correlation analysis of the ammonia ligand in compounds **1** and **2**, however, showed distortion of the octahedral structure; at least two different CoN_5O arrangements exist in compounds **1** and **2**. Consequently, the forbidden IR bands may become IR active; the Co-O and at least two kinds (*cis* and *trans* to oxygen) or more of Co-N stretching modes are expected to appear in the range of 400-600 and 300-500 cm^{-1} in the spectrum of compounds **1** and **2**, respectively. The N-Co-N and N-Co-O bending modes, and their mixed bands, are expected to show up between 150 and 350 cm^{-1}. [7]

The CoN_5O octahedral core vibrational modes are evaluated as follows. The wavenumber order is expected to be ν_{as}Co-N(*cis* to O)>ν_s(Co-N(*cis* to O)>ν_{as}Co-O>ν_s(Co-O)>ν_{as}Co-N(*trans* to O)>ν_sCo-N(*trans* to O) because of the *trans*-effect and possible π interactions in the Co-O bond. Comparing the intensity relations of bands in the IR and Raman (ν_{as} bands are more intense in the IR than in Raman, ν_s bands are more intense in Raman than in IR), the antisymmetric and symmetric Co-N(*cis* to O) stretching modes are assigned at 520 cm^{-1} and 485 cm^{-1} (very weak) in the IR of

compound **1**. There was no antisymmetric Co-N(*cis* to O) band, and the symmetric one appeared at 494 cm^{-1} in the Raman spectrum of compound **1** (Fig. S6). The antisymmetric and symmetric Co-O bands were weak and very weak at 485 cm^{-1} and at 469 cm^{-1}, respectively, in the IR. There was neither a peak of the antisymmetric mode nor a weak peak of the symmetric mode at 472 cm^{-1} in the Raman spectrum of compound **1**. The antisymmetric and symmetric $\nu_{Co\text{-}N}$ (*trans* to O) bands are a shoulder at 465 cm^{-1} and a weak band at 433 cm^{-1} in the IR, respectively, and there was no first mode, whereas there was a second mode band at 434 cm^{-1} in the Raman spectrum of compound **1**. The same distorted CoN_5O skeleton is present in compound **2** and compound **1**; thus, according to the previous considerations, the antisymmetric and symmetric Co-N (*cis* to O) stretching modes were assigned at 526 cm^{-1} and 483 cm^{-1} (very weak) in the IR, and at 526 and 484 cm^{-1} in the Raman spectrum. The antisymmetric and symmetric Co-O bands, as peaks or shoulders (sh), were located at 472 cm^{-1} and 462sh cm^{-1} (IR), and at 461 and 447 cm^{-1} (Raman). The antisymmetric and symmetric $\nu_{Co\text{-}N}$ (*trans* to O) bands/shoulders were at 443sh cm^{-1} and 434 cm^{-1} (IR), and at 454sh and, as a band system, at 447,443 and 439 cm^{-1} (Raman).

The order of CoNO and CoN_2 bending band positions in a CoN_5O octahedron is: *trans-asym*> *cis-asym*> *trans-sym*> *cis-sym*. Accordingly, for compound **1**, the shoulders at 302 cm^{-1} and 276 cm^{-1}, and the peaks at 234 cm^{-1} and 216 cm^{-1} in the IR spectrum, without appropriate Raman bands, may belong to the *trans* or *cis* antisymmetric modes of CoNO and CoN_2 linkages, respectively. For compound **2**, the *trans* and *cis* antisymmetric CoON and CoN_2 modes were found at 312 and 296sh cm^{-1}, and 231 and 220 cm^{-1} in its IR spectrum, respectively. The weak or missing Raman peaks (Fig. S6) confirm the antisymmetric nature of these modes. The symmetric CoNO and CoN_2 bending modes are probably located below 200 cm^{-1} in the spectra of both compounds.

Text S4. The correlation analysis of water molecules in compounds **1** and **2**.

The correlation analysis of water molecules showed that all three bands (ν_1-ν_3) of crystalline water and the vibration rocking, twisting, and wagging modes are active in the IR and Raman spectra for both compounds. The ν(OH) modes (the antisymmetric and symmetric ones) were found as an overlapped complex band at 3350 and 3424 cm^{-1} for compounds **1** and **2**, respectively, the scissoring mode coinciding with $\delta_{as}(NH_3)$ as has already been discussed above. The correlation analysis results for crystalline water and single Co^{III} and chloride ions for compounds **1** and **2** are shown in Figs. S12.

Table S15. The assignments of ammonia vibrations for compounds **1**, **1-D**, **2** and **2-D**, and their isotope shift parameter (φ).

Mode	**1**	**1-D**	*φ*	**2**	**2-D**	*φ*
ν_{as}(N-H)	3238	2520<br>2426	1.29-1.33	3208	2368<br>2342	1.35<br>1.37
ν_s(N-H)	3170	2361<br>2322<br>2275	1.34-1.39	3132<br>3085	2324<br>2261	1.35<br>1.36
δ_{as}(N-H)	1580*<br>1541**	1162	1.33	1599*<br>1562**	1167<br>1140	1.37
δ_s(NH)	1316<br>1299	1027<br>1000	1.28<br>1.29	1312<br>1289	1022<br>1008	1.28<br>1.28
$\rho(NH_3)$	813**	666	1.22	815	665	1.22

*Coinciding bands with $\delta_{as}(H_2O)$; **Calculated δ_{as}(N-H) position (shoulder) from the isotope shift ratio

Table S16. The summary of weight losses during the decomposition of Compound **1.**

Lost component	Compound **1**		
	Calculated	Exp.	
		Inert	Air
$-H_2O$	6.09		
$-5NH_3$	28.76		
-1.5O	8.12		
-Cl	12.01		
Sum with r due: $CoO+0.5Cr_2O_3$ = $CoCrO_{2.5}$ = $Co[Co_{0.5}Cr_{1.5}O_{3.75}]$	54.98	50.4	57.2

Table S17. The summary of weight losses during the decomposition of Compound **2.**

Lost component	Compound **2**		
	Calculated	Exp.	
		Inert	air
$-4H_2O$	11.31		
$-10NH_3$	26.72		
-8.9O	20.37		
Sum with r due: $2CoO+1.5Cr_2O_3$ = Co_2Cr_3O5=$Co[Co_{0.2}Cr_{1.8}O_{3.90}]$	58.44	43.9	43.3

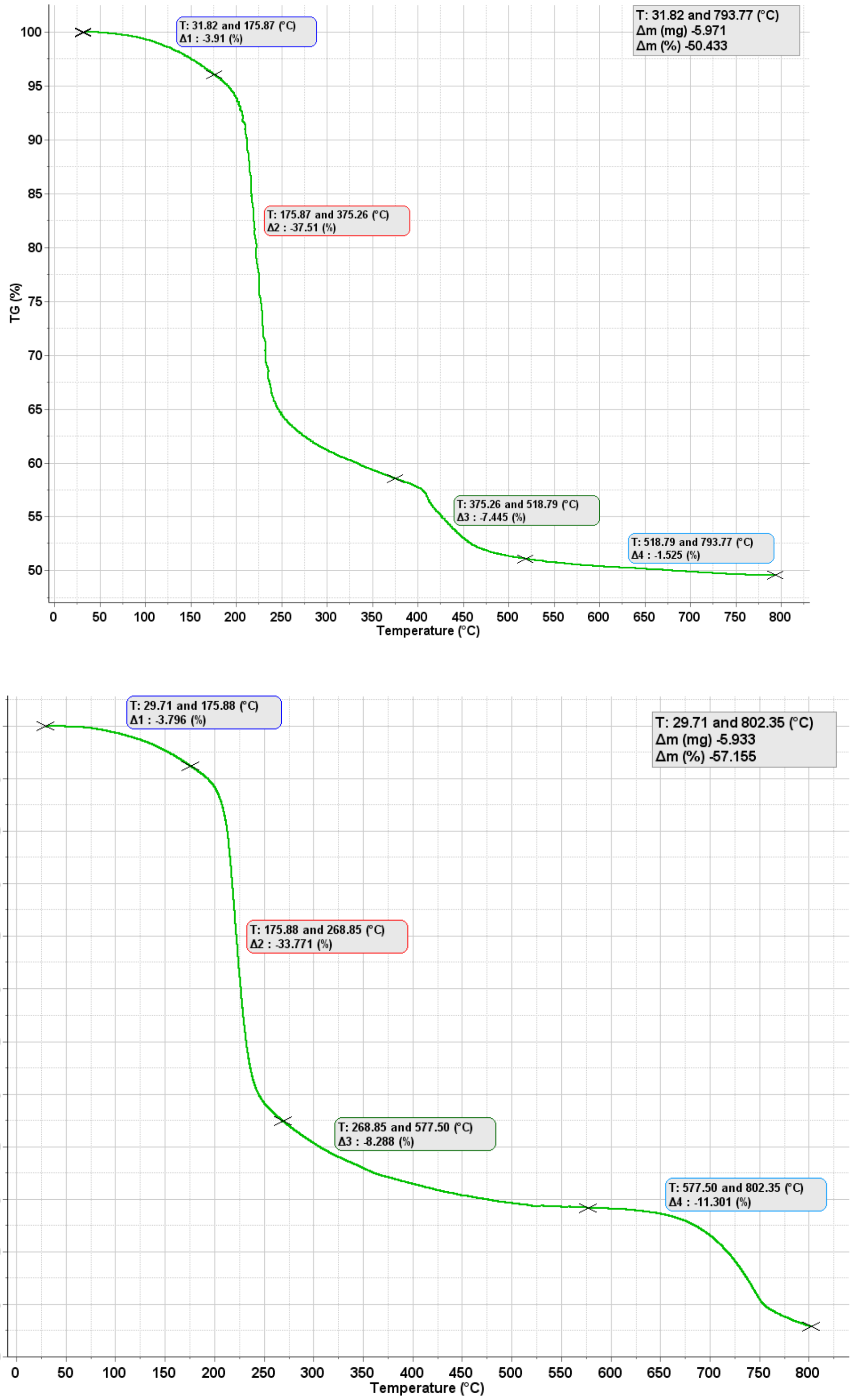

Figure S21. The oxidative (a) and inert (b) atmosphere TG curve of compound **1**.

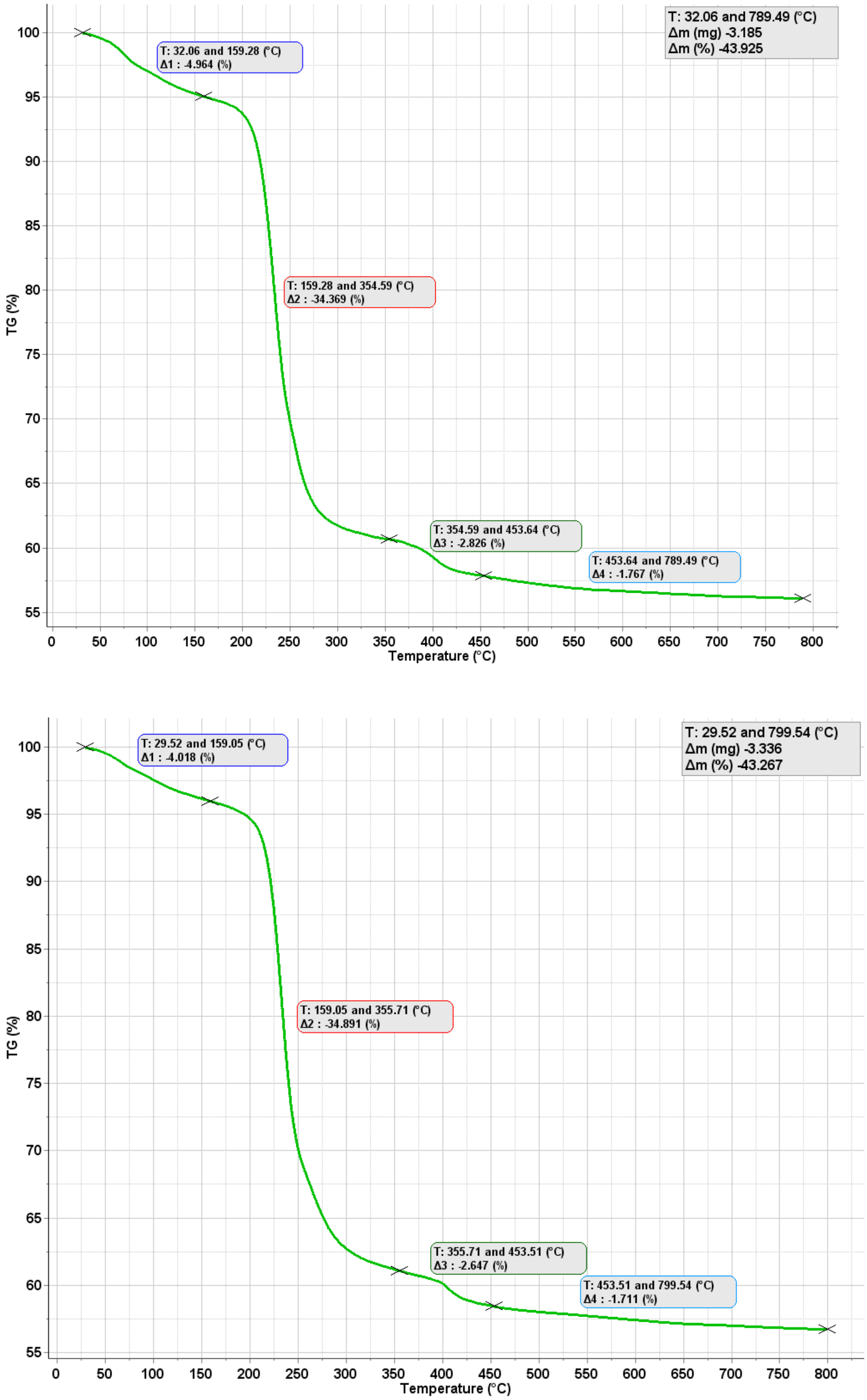

Figure S22. The oxidative (a) and inert (b) atmosphere TG curve of compound **2**.

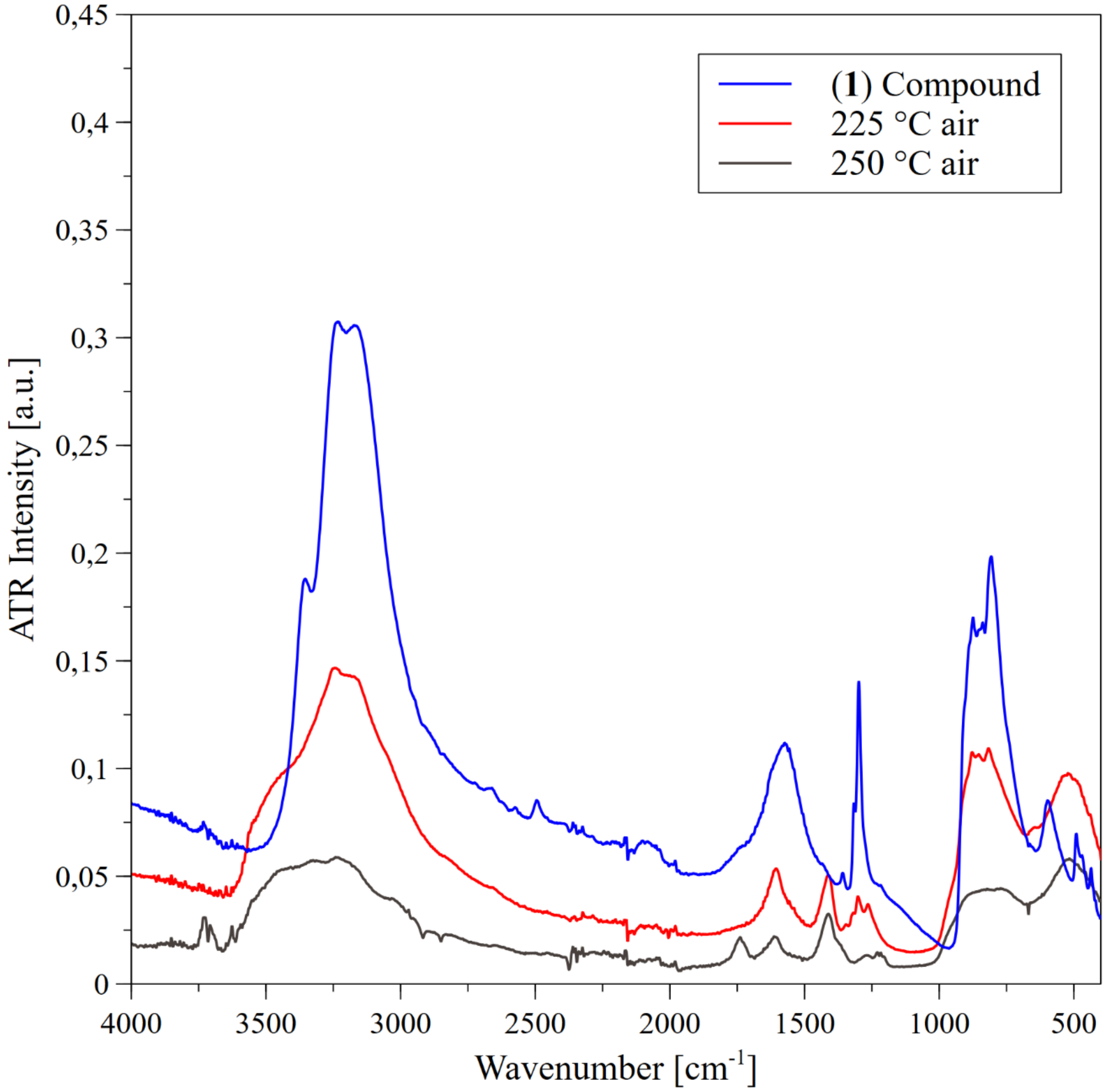

Figure S23. The IR spectra of the thermal decomposition intermediates of compound **1** in air.

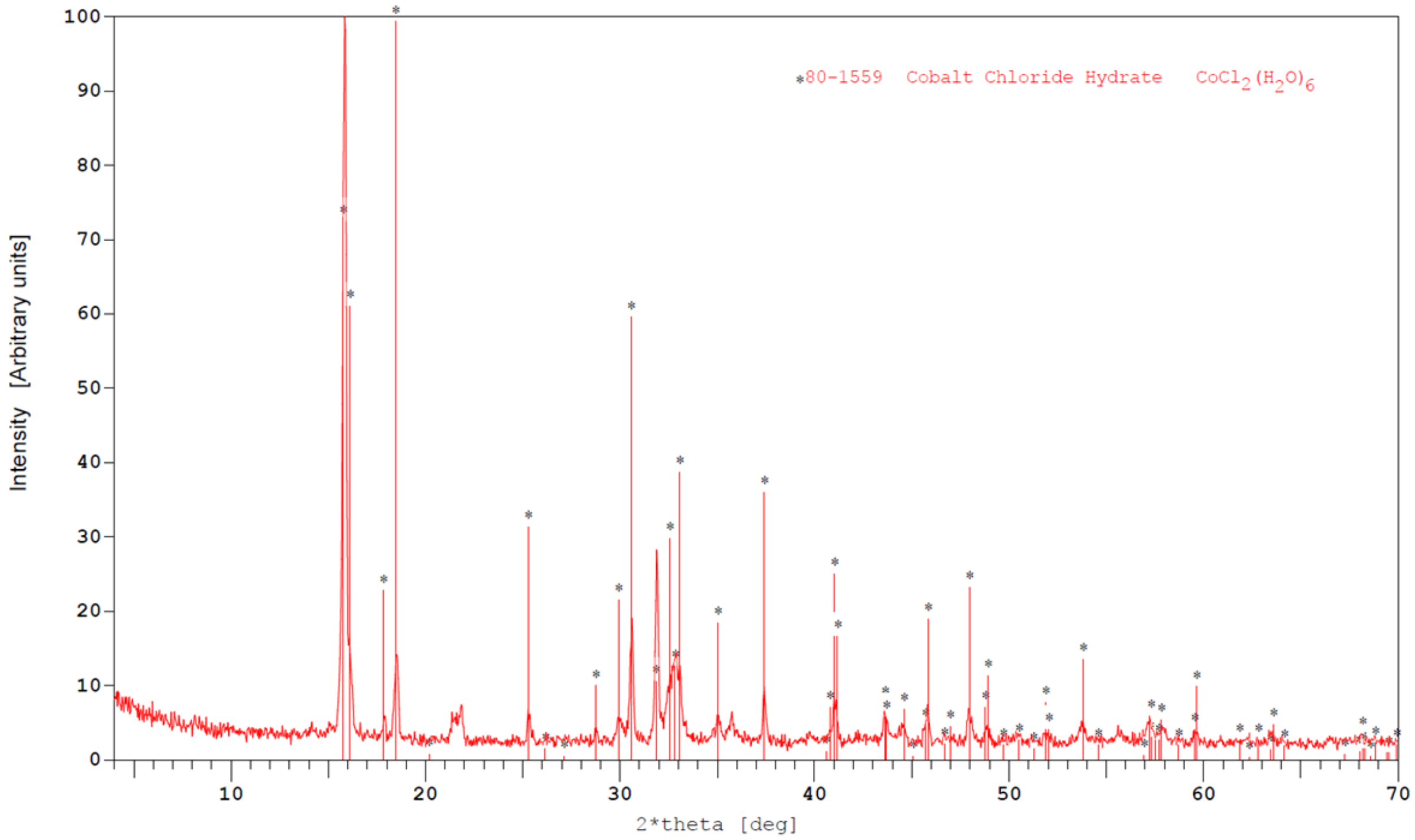

Figure S24. The PXRD of the leached phase produced during the thermal decomposition of compound **1** in air at 250 °C.

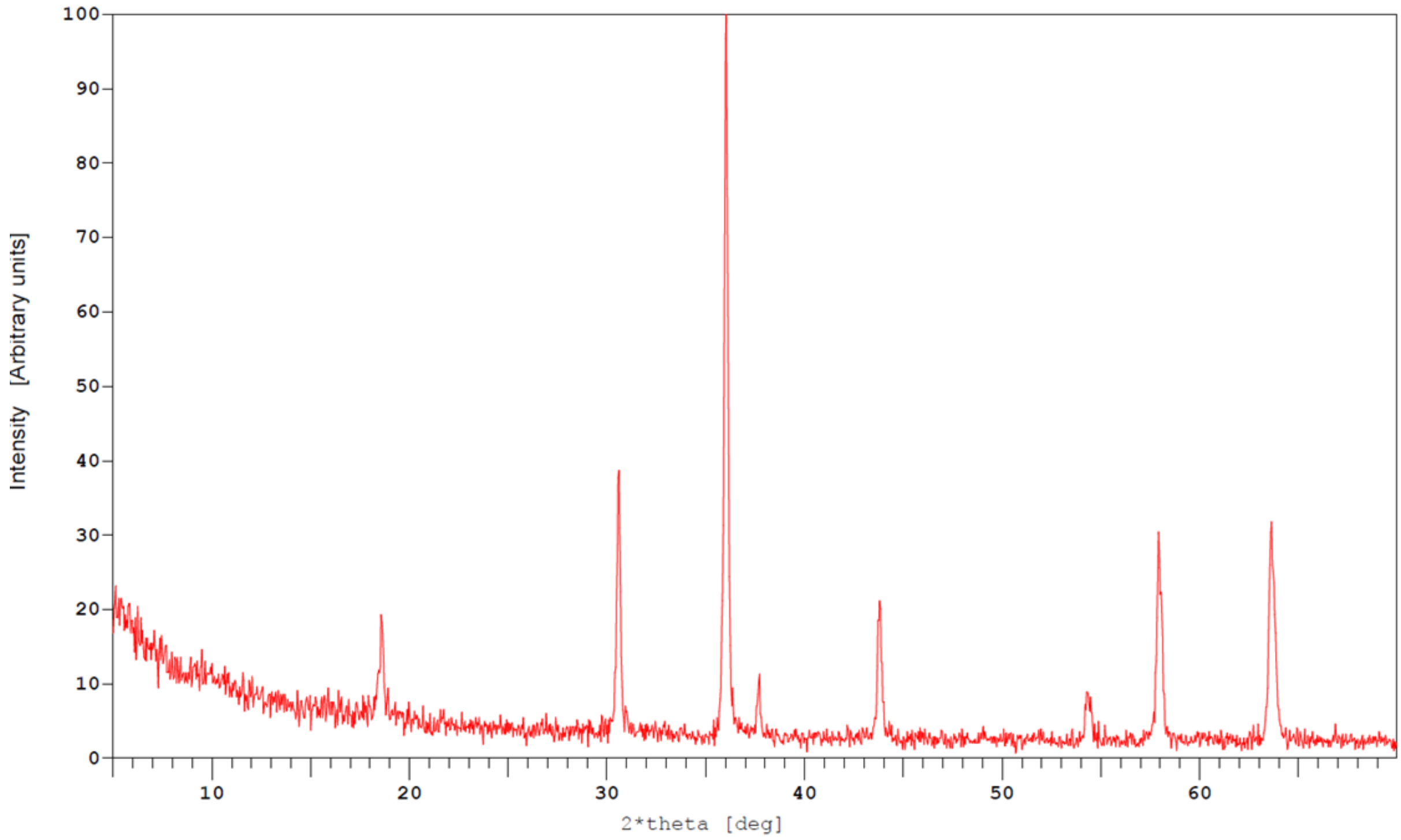

Figure S25. The PXRD of the thermal decomposition product of compound **1** in air at 800 °C.

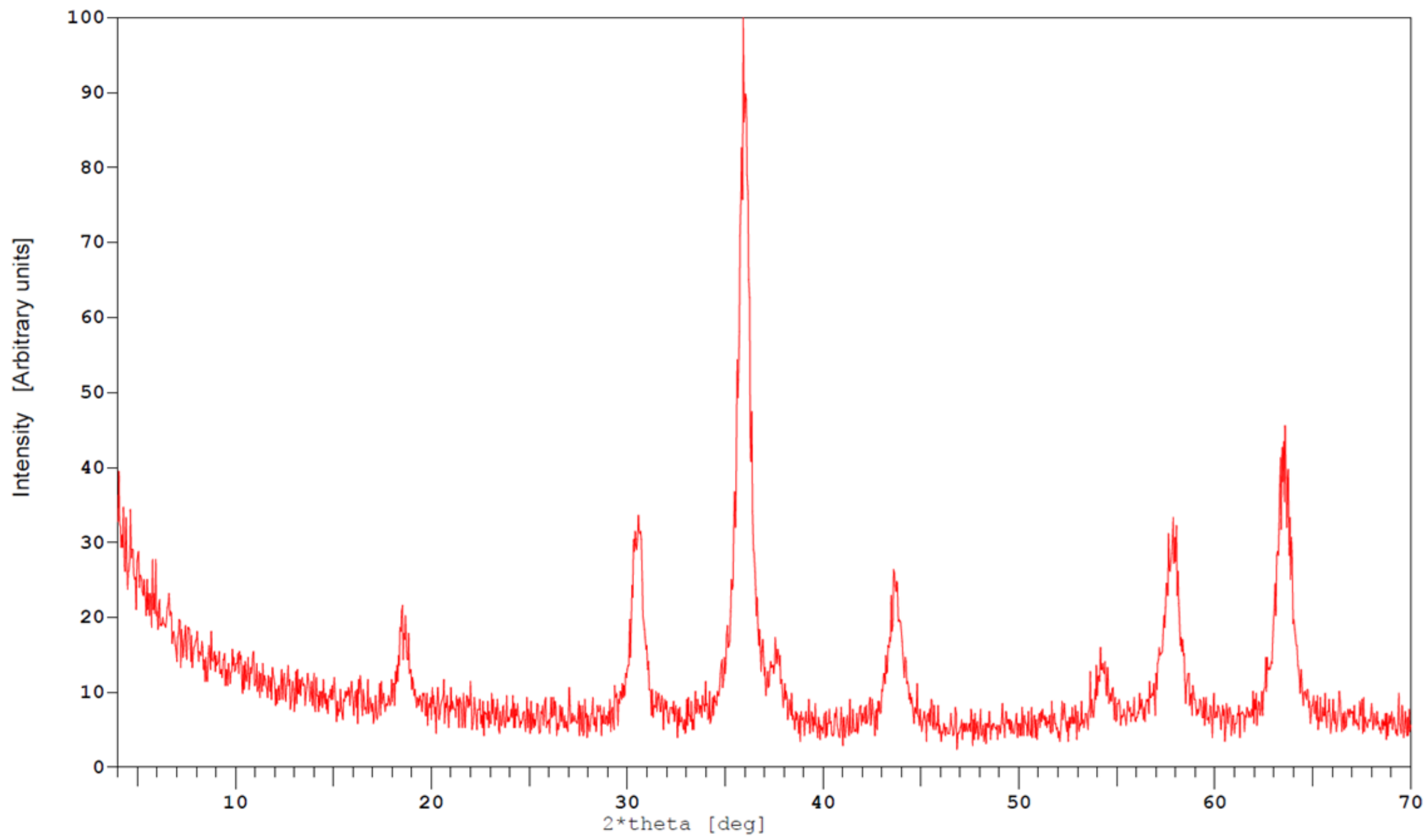

Figure S26. The PXRD of the thermal decomposition product of compound **1** in air at 500 °C after the aqueous leaching.

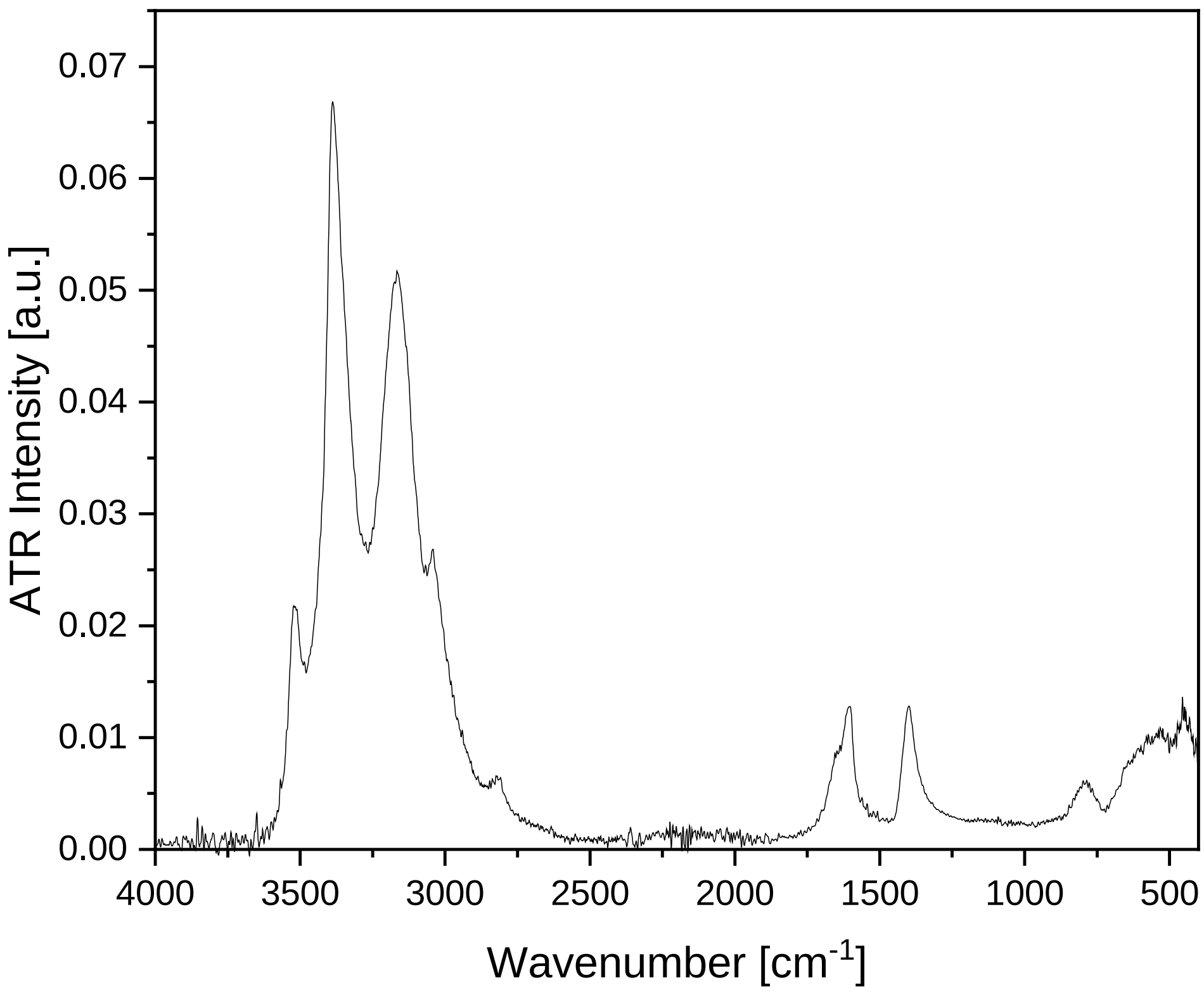

Figure S27. The IR spectra of the thermal decomposition intermediates of compound **1** in inert atm..

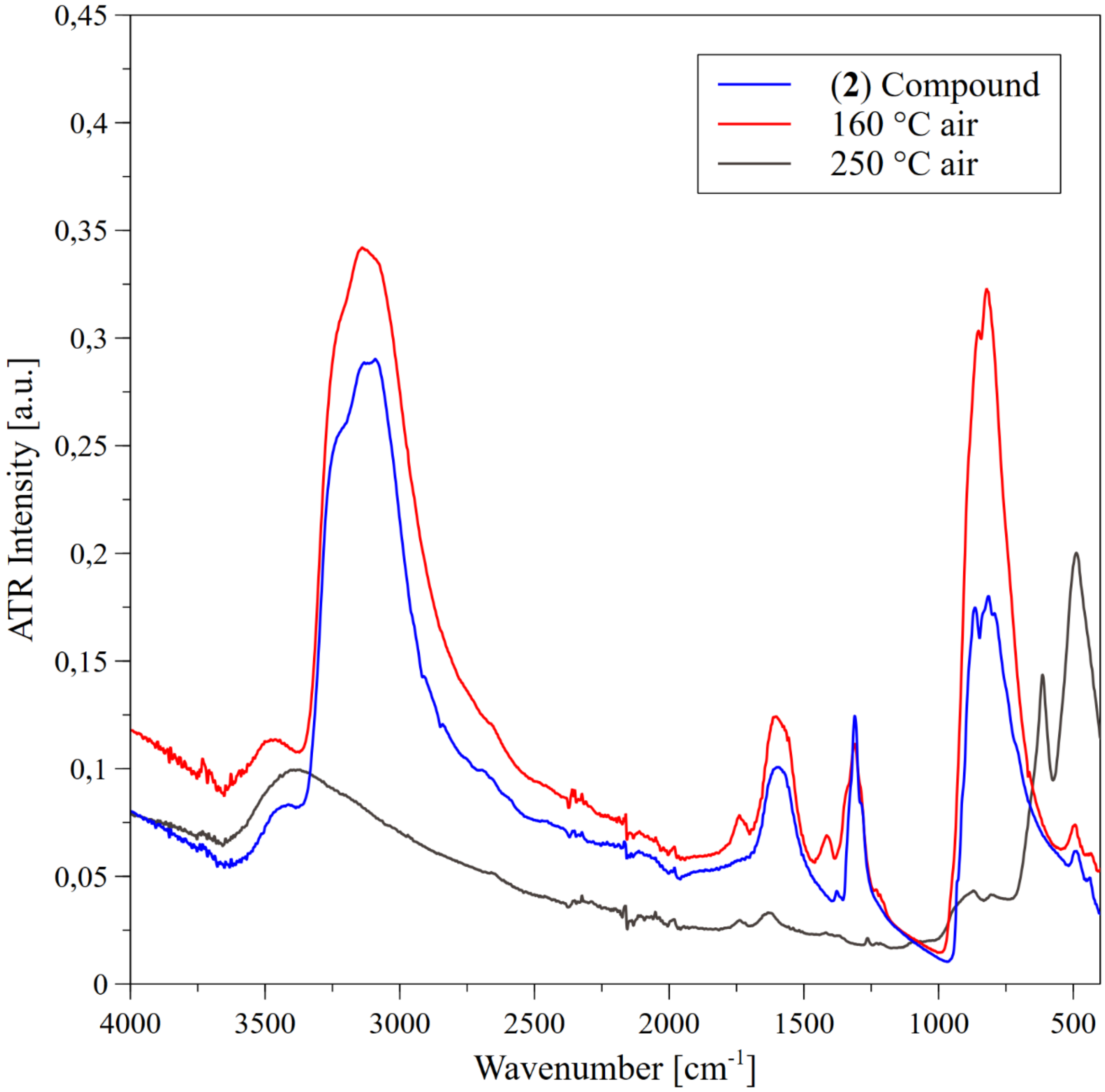

Figure S28. The IR spectra of the thermal decomposition intermediates of compound **2** in air.

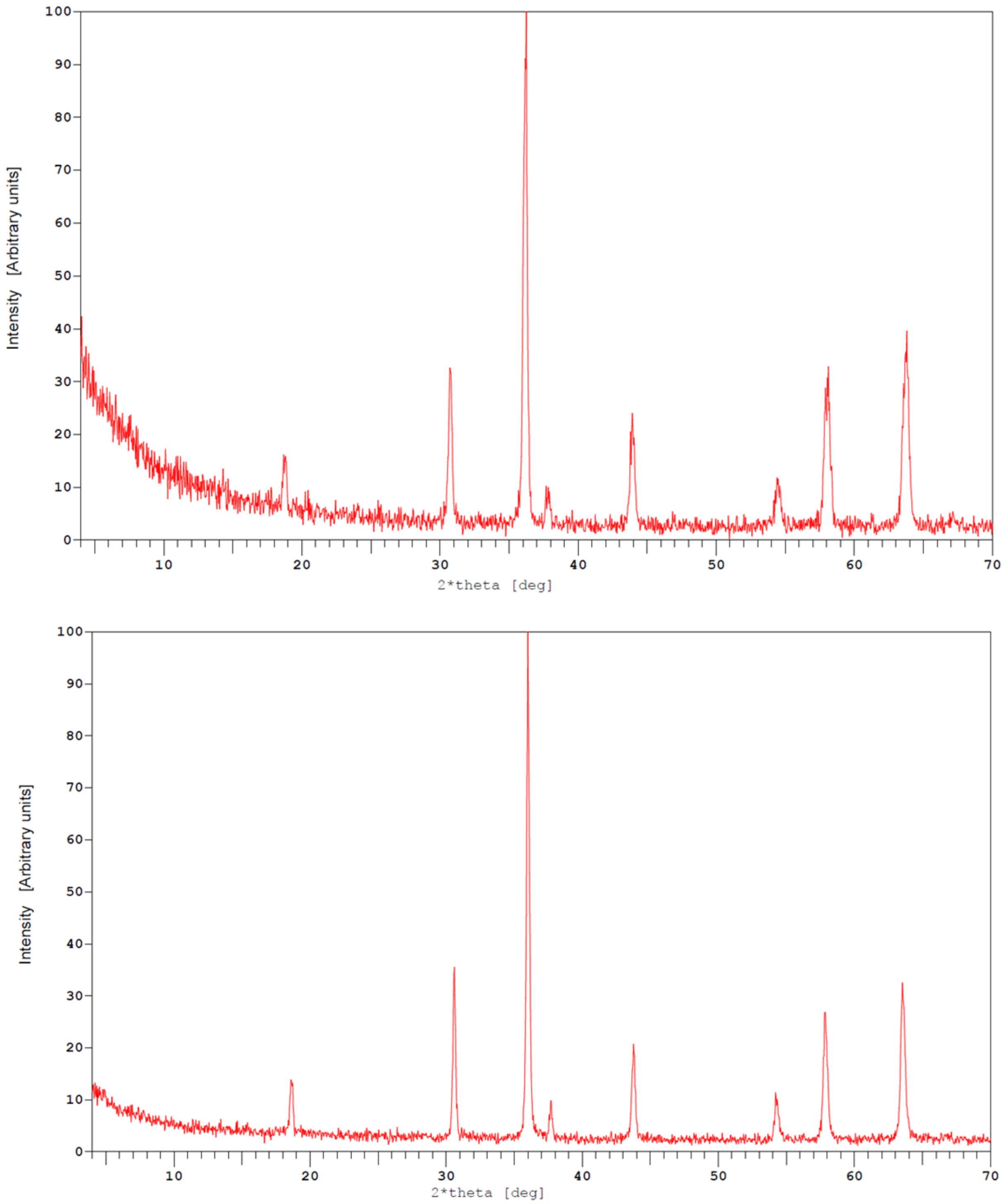

Figure S29. The PXRD of the thermal decomposition product of compound **2** in **(a)** air and (b) inert atm. at 800 °C.

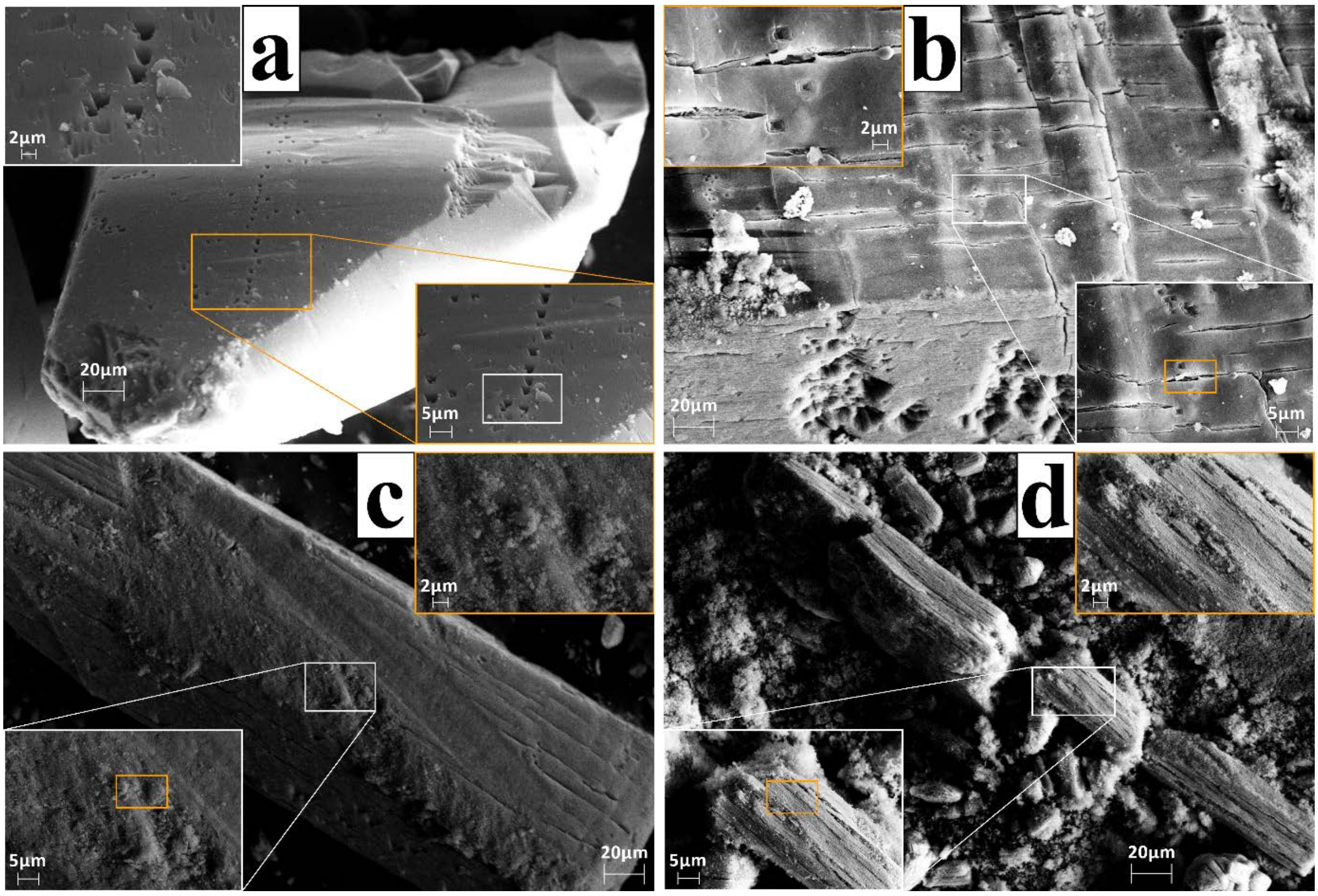

Figure S30. The SEM pictures of compound 1 (a) and its thermal heat treatment product at 250 °C (b), 500 °C (c), and 800 °C (d) in oxidative atm..

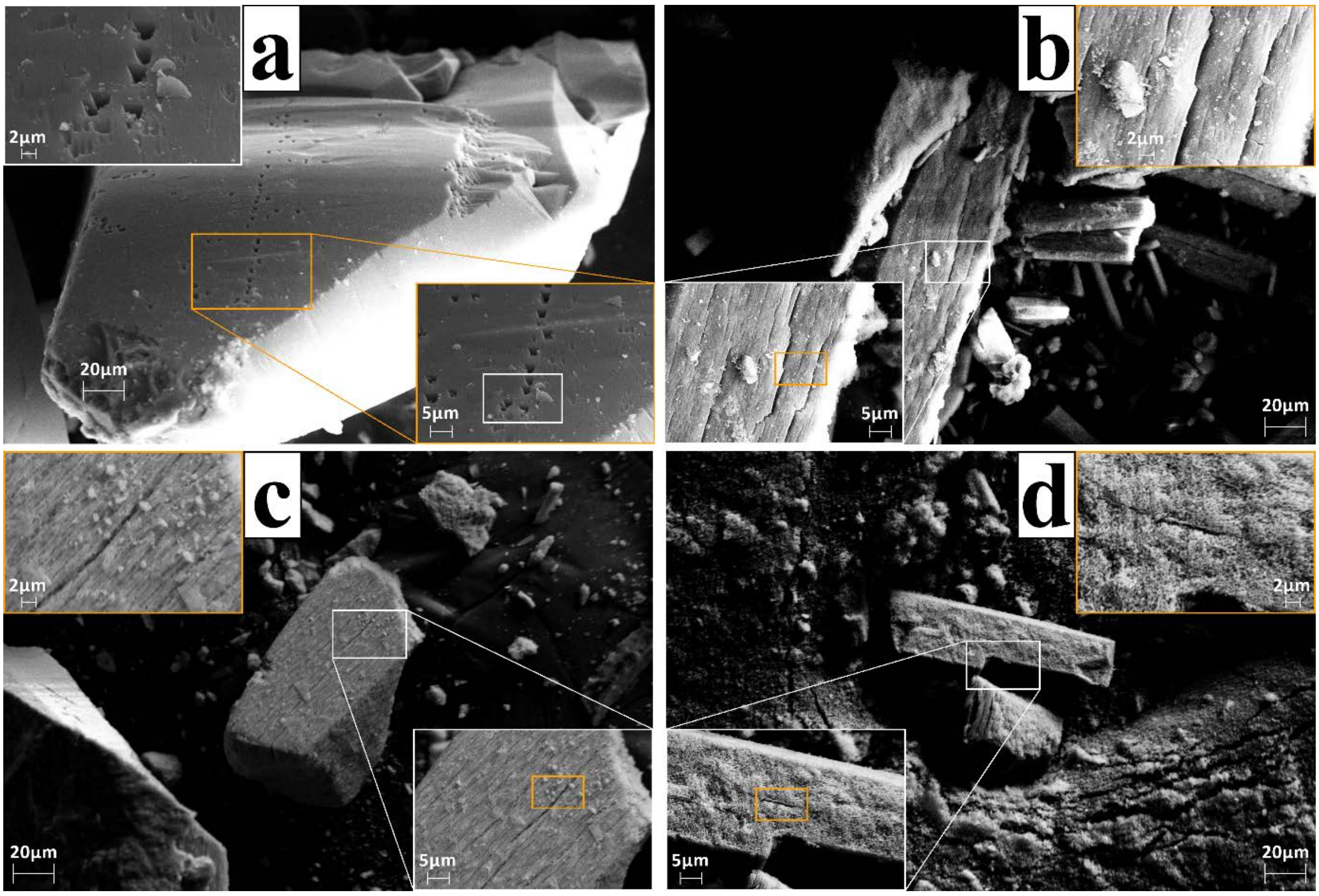

Figure S31. The SEM pictures of compound 1 (a) and its thermal heat treatment product at 250 °C (b), 500 °C (c), and 800 °C (d) in inert atm..

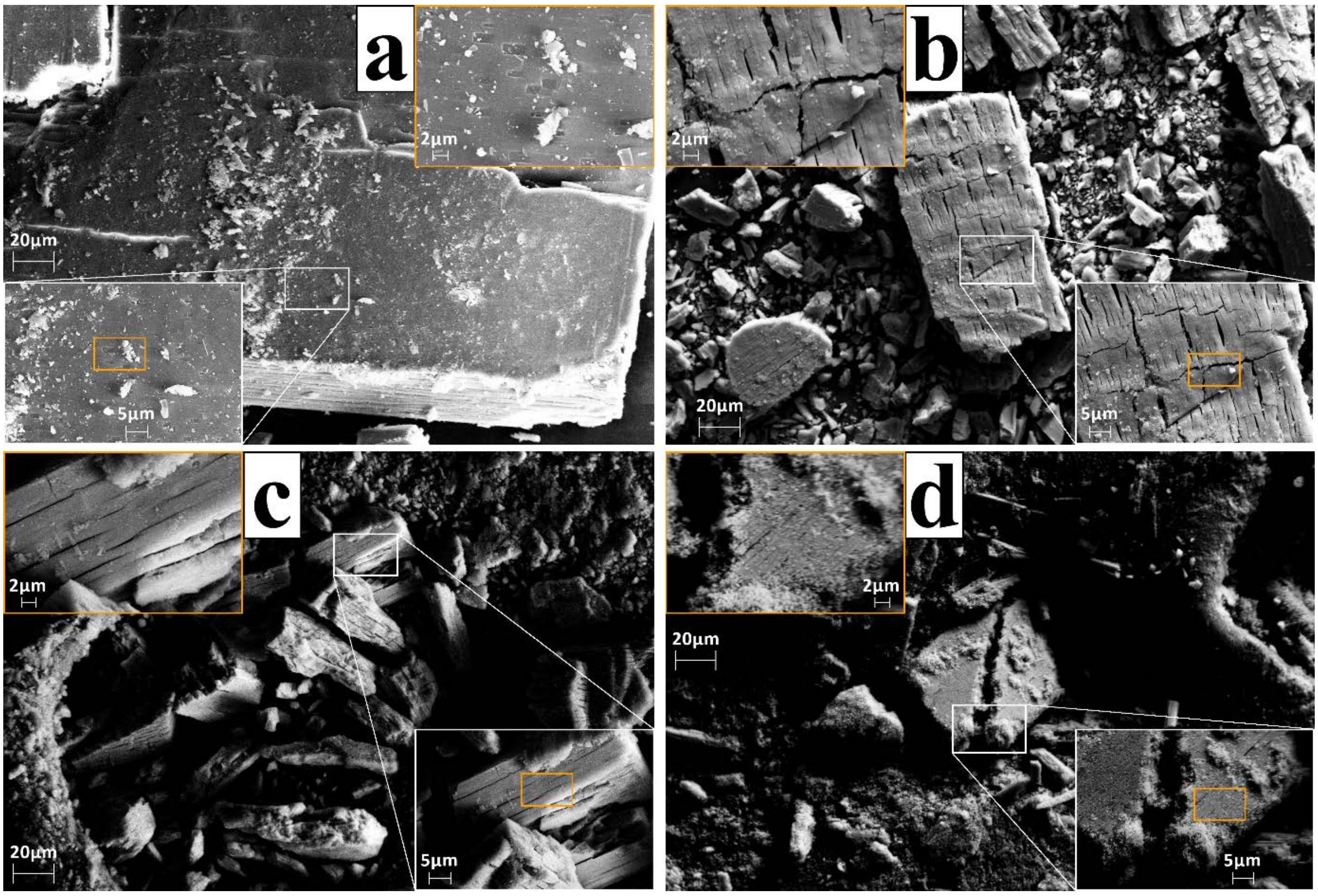

Figure S32. The SEM pictures of compound **2** (a) and its thermal heat treatment product at 250 °C (b), 500 °C (c), and 800 °C (d) in oxidative atm..

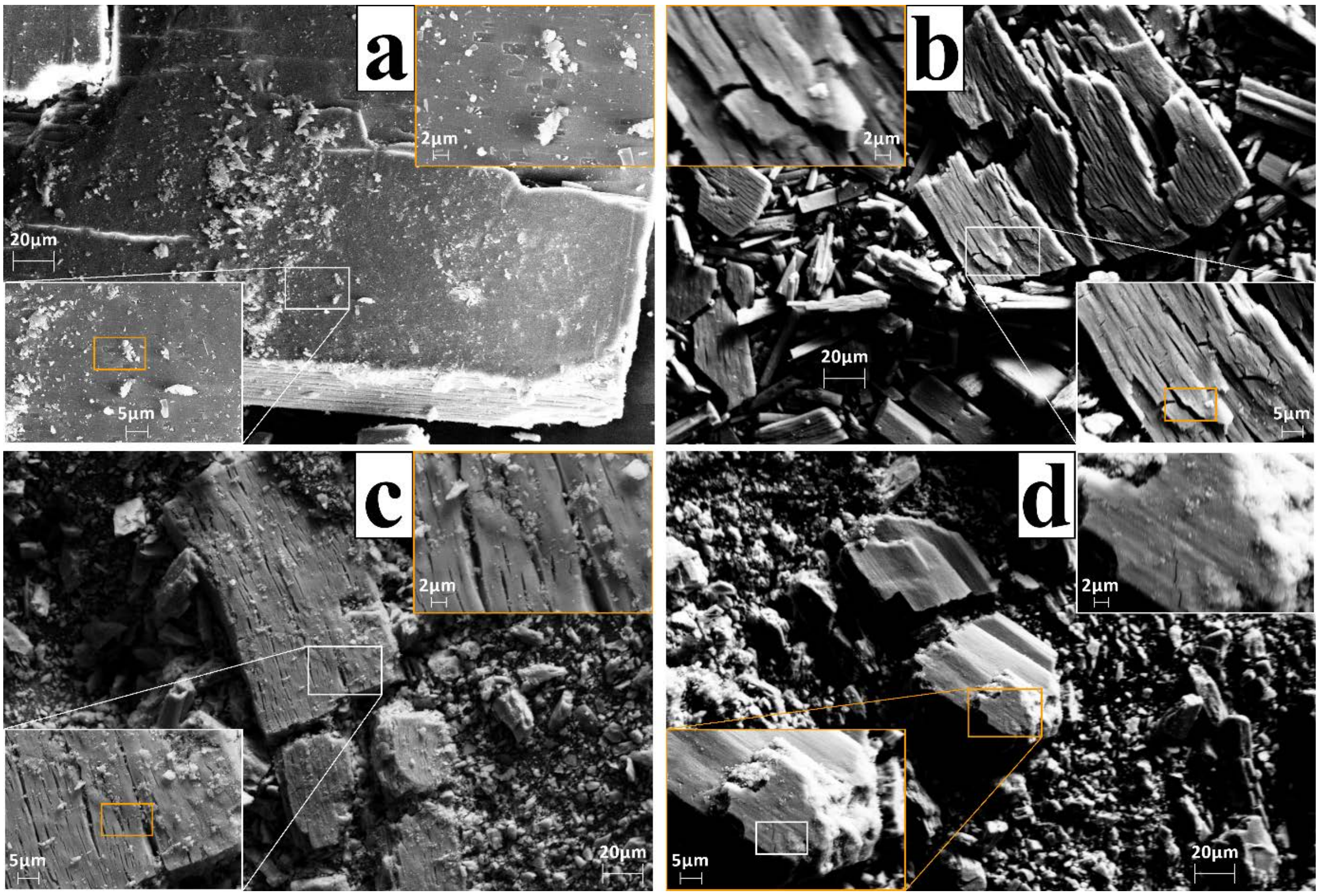

Figure S33. The SEM pictures of compound 2 (a) and its thermal heat treatment product at 250 °C (b), 500 °C (c), and 800 °C (d) in inert atm..

Table S18. The BET surface area of the different Co-Cr oxides in m^2/g.

Compound	**1**		**2**	
Heat treatment atmosphere and temperature	air	inert	air	inert
250 °C	9.3	12.4	53.9	77.5
500 °C	58.4	83.2	41.7	68.5
800 °C	6.9	7.1	7.2	62.5

Table S19. The summary of surface areas of co-chromites prepared in various reaction routes.

Method	Starting materials	Preparation of the spinel	Heat treatment / Calcination temperature	Specific Surface Area	Ref.
**Hydrothermal**	$Co(NO_3)_2$, $Cr(NO_3)_3$	$Co(NO_3)_2$ and $Cr(NO_3)_3$ were dissolved in dist. water. KOH was added until precipitation was complete. The hydroxides were transferred to a Teflon-lined steel autoclave. The autoclave was sealed and kept at temperatures ranging from 270 °C to 300 °C. The products were washed with dist. water and dried at 80 °C.	270 °C - 300 °C	309.28 – 371.9 m^2/g	8
	$Co(NO_3)_2$, $Cr(NO_3)_3$	$Co(NO_3)_2$ and $Cr(NO_3)_3$ were dissolved in 100 mL of dist. water. NH4F and urea were mixed separately; the two solutions were mixed and kept in a Teflon-lined stainless-steel autoclave at 120 °C for 24 h. The obtained sample was rinsed with dist. water and ethanol, dried, then annealed at 600 °C for 2 h.	120 °C 24 h, then 600 °C, 2 h	27 m^2/g	9
	$CoCl_2$, K_2CrO_4	$CoCl_2$ and K_2CrO_4 solutions were mixed with continuous stirring for 2 h. The resulting solution was transferred to a Teflon-lined stainless-steel autoclave and kept at 120 °C for 12 h. The resulting precipitate was then washed with a water-ethanol mixture and dried. The obtained powder was annealed at 700 °C for 4 h.	120 °C, 12 h, then 700 °C, 4 h	7.2 m^2/g	10
	$Co(NO_3)_2$, $Cr(NO_3)_3$ ethylene glycol	$Co(NO_3)_2$ and $Cr(NO_3)_3$ were added to dist. water with a const. stirring for 90 min. Ethylene glycol was added to the mixture. The solution was sealed in a hydrothermal reactor and was heated in an oven at 190 °C for 6 h. The obtained product was then rinsed with dist. water and ethanol then dried. This was followed by annealing at 760 °C.	190 °C, 6h, then 760 °C	215 m^2/g	11
**Coprecipitation**	$Co(NO_3)_2$, $Cr(NO_3)_3$	$Co(NO_3)_2$ and $Cr(NO_3)_3$ were dissolved in dist. water. Diluted ammonia was added until the pH was 9.30. The precipitates were filtered, washed and dried. The dried materials were calcined at 700 °C for 4 h.	700 °C, 4 h	12.8 – 38.6 m^2/g	12

	$Co(NO_3)_2$, $Cr(NO_3)_3$	$Co(NO_3)_2$ and $Cr(NO_3)_3$ were dissolved in dist. water. A small amount of conc nitric acid was added, then the pH of the solution was adjusted to 10 with NaOH. The filtrate was washed, until the pH becomes neutral and dried at 100 °C overnight. The solid was then calcined at 700 °C for 6 h.	700 °C, 6 h	46.9 m^2/g	13
	$Co(NO_3)_2$, $Cr(NO_3)_3$	$Cr(NO_3)_3$ was dissolved in dist. water. To this solution, $Co(NO_3)_2$ solution was added slowly. Diluted aqueous ammonia was added to the mixture until the pH of the solution became 9. The precipitate was filtered, washed several times and dried in an oven at 120 °C overnight, followed by calcination at 600 °C for 2 h.	600 °C, 2 h	43.8 m^2/g	14
	$CoCl_2$, K_2CrO_4	$CoCl_2$ and K_2CrO_4 solutions were mixed. The pH of the solution was adjusted to 9 with diluted ammonia solution, then the stirring was continued for 2 h to ensure the completion of the reaction. The precipitate was washed with water-ethanol mixture, dried, then annealed at 700 °C for 4 h.	700 °C, 4h	37.1 m^2/g	15
**Sol-gel**	$Co(NO_3)_2$, $Cr(NO_3)_3$	$Co(NO_3)_2$ and $Cr(NO_3)_3$ were dissolved in ethanol, with propylene oxied added as a chelating agent. The mixed solution was ultrasonicated and the resulted gel was aged for 24 h. Then it was dried at 90 °C for 24 h and calcined at different temperatures of 400, 550 and 700 °C for 1, 4 and 8 hours.	400-700 °C, 1-8 h	37.3 – 143.8 m^2/g	16
	$Co(NO_3)_2$, $Cr(NO_3)_3$	$Co(NO_3)_2$ and $Cr(NO_3)_3$ were dissolved in dist. water in stoichiometric amounts and the appropriate amount of glucose was added. The solvent evaporation was followed by a drying step at 110 °C. The solid was then calcined firstly at 500 °C for 3 h then at 1400 °C for 3 h.	500 °C, 3 h, then 1400 °C, 3 h	0.4 m^2/g	17
	Co-acetylacetonate, Cr-acetylacetonate	$Co(acac)_2$ and $Cr(acac)_3$ were dissolved in propionic acid. The solution was evaporated and the resulting gel was treated with liquid nitrogen. The cooled solid was shredded and calcined at two different temperatures ranging from 450 °C to 685 °C.	450, 685 °C	9 - 103 m^2/g	18
	Co(Ac)2, Cr(Ac)3, citric acid, ethylene glycol	$Co(Ac)_2$ and $Cr(Ac)_3$ were dissolved in dist. water. Citric acid was added, followed by a dropwise addition of ethylene glycol. The solution was heated and stirred until it was viscous then dried in oven at 180 °C for 10 h. The resulting solids were calcined in a furnace at temperatures ranging from 500 to 600 °C for 4 h.	500-600 °C, 4 h	36.6 – 74.6 m^2/g	19

**Solution combustion**	$Co(NO_3)_2$, $Cr(NO_3)_3$ Tetraformal trisazine	$Co(NO_3)_2$ and $Cr(NO_3)_3$ were dissolved in dist. water. To the solution, tetraformal trisazine was added, and the mixture was put in a furnace set to 350 °C	350 °C (the reactor heated up to 900 °C under the reaction)	20.5 m^2/g	20
	$Co(NO_3)_2$, $Cr(NO_3)_3$ urea	$Co(NO_3)_2$, $Cr(NO_3)_3$, and urea were dissolved in dist. water. The solution was then heated to 350 °C. The solution boiled, dehydrated and decomposed.	350 °C	8.3 m^2/g	21
	$Co(NO_3)_2$, $Cr(NO_3)_3$ urea	$Co(NO_3)_2$ and $Cr(NO_3)_3$ were dissolved in dist. water along with urea. The solution was put into a furnace heated to 430 °C for 20 min. The resultant powder was then ground and calcined at 550 °C for 3 h.	430 °C, then 550 °C, 3 h	11.7 m^2/g	22
**Microwave**	$CoCl_2$, K_2CrO_4	$CoCl_2$ was dissolved in dist. water, to which a solution of K_2CrO_4 was added. The mixture was transferred to a microwave oven and heated for 30 s at a power of 150 W. The resultant precipitate was washed with a water-ethanol mixture and dried. Then the solid was annealed at 700 °C for 4 h.	700 °C, 4h	20.0 m^2/g	15
**Precursor**	$Co(NO_3)_2$, $Cr(NO_3)_3$, tartaric acid, gluconic acid	$Co(NO_3)_2$ and $Cr(NO_3)_3$ were dissolved in minimal amount of dist. water and mixed with aqueous solution of tartaric/gluconic acid. Ethanol was added to the mixture until a lavender-colored precipitate was formed. Ammonia-ethanol mixture was added to the solution until the pH of the solution was raised to 6. After 24 h of standing at 4 °C the solids were heat treated at two different temperatures, 700 and 800 °C for 1 and 4 hours.	700 °C, 1h and 800 °C, 4h	14.9 – 50.6 m^2/g	23
	$Co(NO_3)_2$, $Cr(NO_3)_3$, citric acid, glucose, acrylamide	$Co(NO_3)_2$, $Cr(NO_3)_3$, citric acid, glucose, and acrylamide were dissolved in dist. water. The mixture was heated to 80-100 °C, the resultant gel was dried and sintered at 800 °C for 5 h.	800 °C, 5h	34.7 m^2/g	24
**Thermolysis**	$CoCl_2$, $CrCl_3$, 2-mercaptopyridine n-oxide Na	$CoCl_2$ and $CrCl_3$ solutions were mixed and 2-Mercaptopyridine N-oxide was added. The flask was heated up to 100 °C with an IR heating lamp for 5 minutes. The resultant precipitate was washed with dist. water and calcined at temperatures of 600 and 800 °C for 3 to 5 hours.	600 - 800 °C, 3 - 5 h	78.2 m^2/g	25

Table S20. The XPS study of the different Co-Cr oxides.

Sample	Cr_2O_3	Co_2O_3	Co	O bal.
Comp. **1** at 500 °C in air	33.2 %	66.8 %	-	0.90
Comp. **2** at 500 °C in air	49.5 %	36.4 %	14.1 %	1.09
Comp. **2** at 500 °C in N_2	21.4 %	47.7 %	11.0 %	0.78

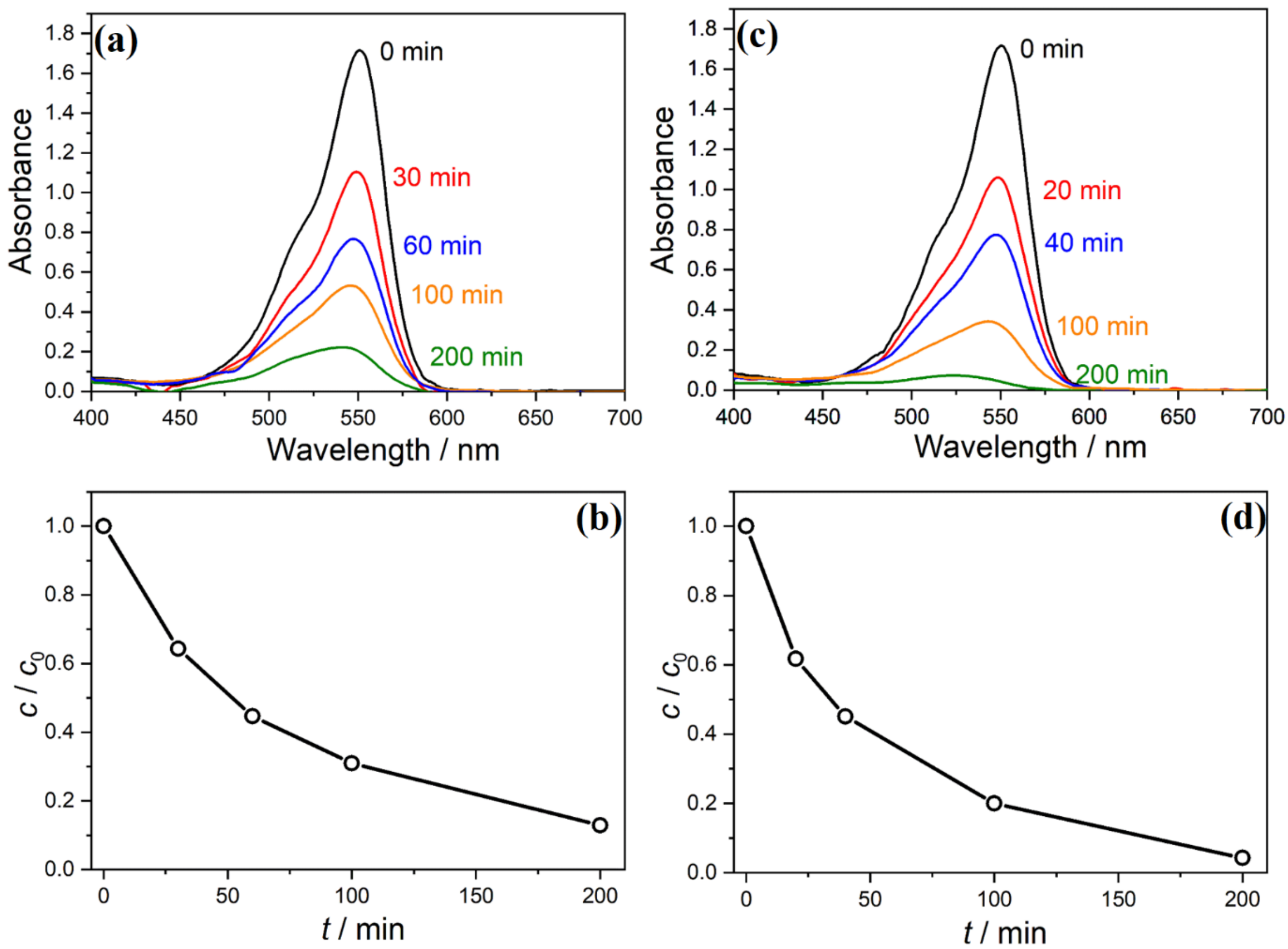

Figure S34. The results of visible light photocatalyst test in a heterogeneous photo-Fenton reaction with the rhodamine B dye: sample *CrO_4-Cl-500-air* **(a)** and **(b)**, *CrO_4- CrO_4-500-air* **(c)** and **(d)**.

Table S21. The results of the photocatalytic degradation of the Rhodamine B with the various catalysts.

**Catalyst**	**Efficiency [%]**	**Rate constant (*k*) ($\cdot 10^{-2}$) [min^{-1}]**	**BET surface area [m^2/g]**
CrO_4-Cl-250-a	97.4	1.845±0.5	9.3
CrO_4-Cl-250-i	79.4	0.803±0.06	12.4
CrO_4-Cl-370-a	50.7	0.335±0.07	65.8
CrO_4-Cl-500-a	87.1	0.995±0.6	58.4
CrO_4-Cl-500-a (after leaching)	73.9	0.686±0.044	41.9
CrO_4-Cl-500-i	22.5	0.109±0.003	83.2
CrO_4-CrO_4-250-a	93.8	1.394±0.3	53.9
CrO_4-CrO_4-250-i	26.4	0.156±0.003	77.5
CrO_4-CrO_4-500-a	95.7	1.529±0.5	41.7
CrO_4-CrO_4-500-i	19.6	0.129±0.006	68.5

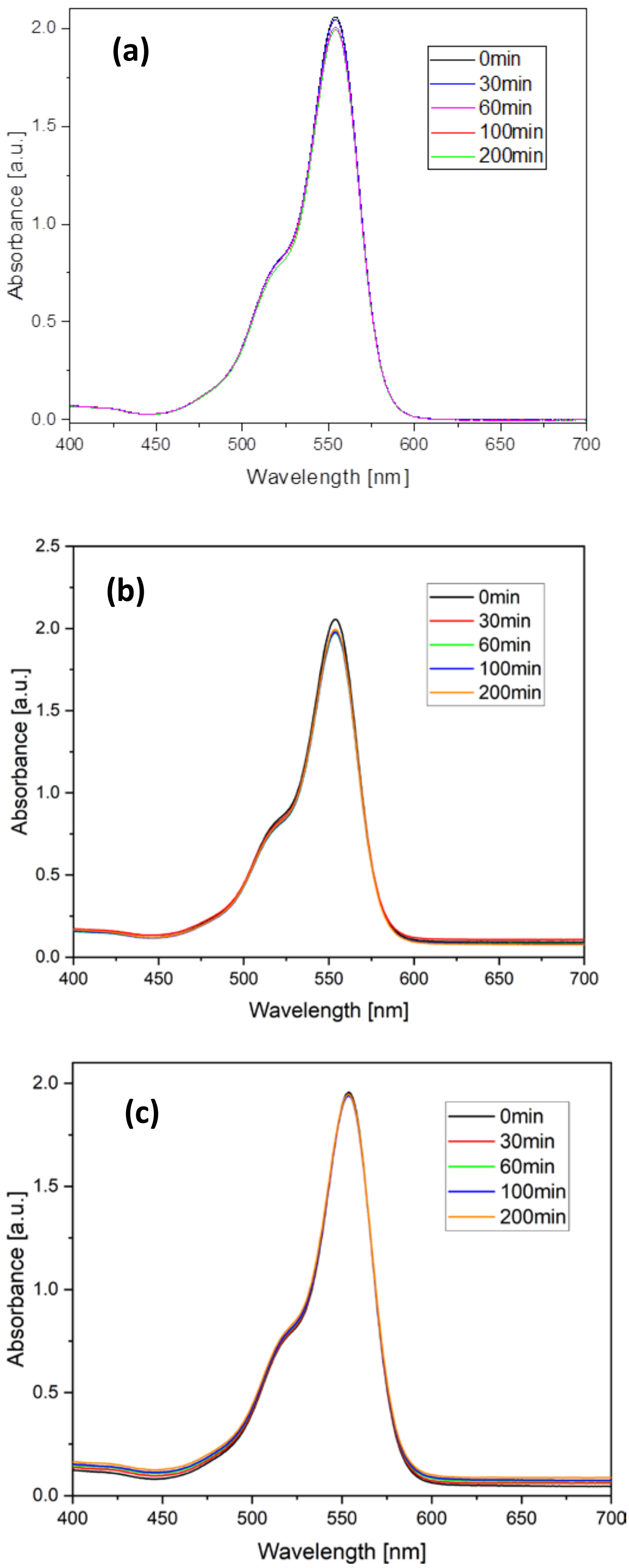

Figure S35. The results of visible light photocatalyst test in a heterogeneous reaction with the rhodamine B dye: without a catalyst with H_2O_2 and irradiation **(a)**, without H_2O_2 with catalysts and irradiation **(b)**, with catalyst and H_2O_2 but without irradiation with visible light **(c)**.

Table S22. The ICP studies of the reaction solution after catalytic test done with CrO_4-Cl-*250-a* and CrO_4-Cl-*250-a*.

**mg/liter (ppm)**	**Cr**	**Co**
Water (background)	*0,001*	*0,009*
CrO_4-Cl-*250-a*	**11,47**	**15,14**
CrO_4-Cl-*250-a*	**3,10**	**15,00**

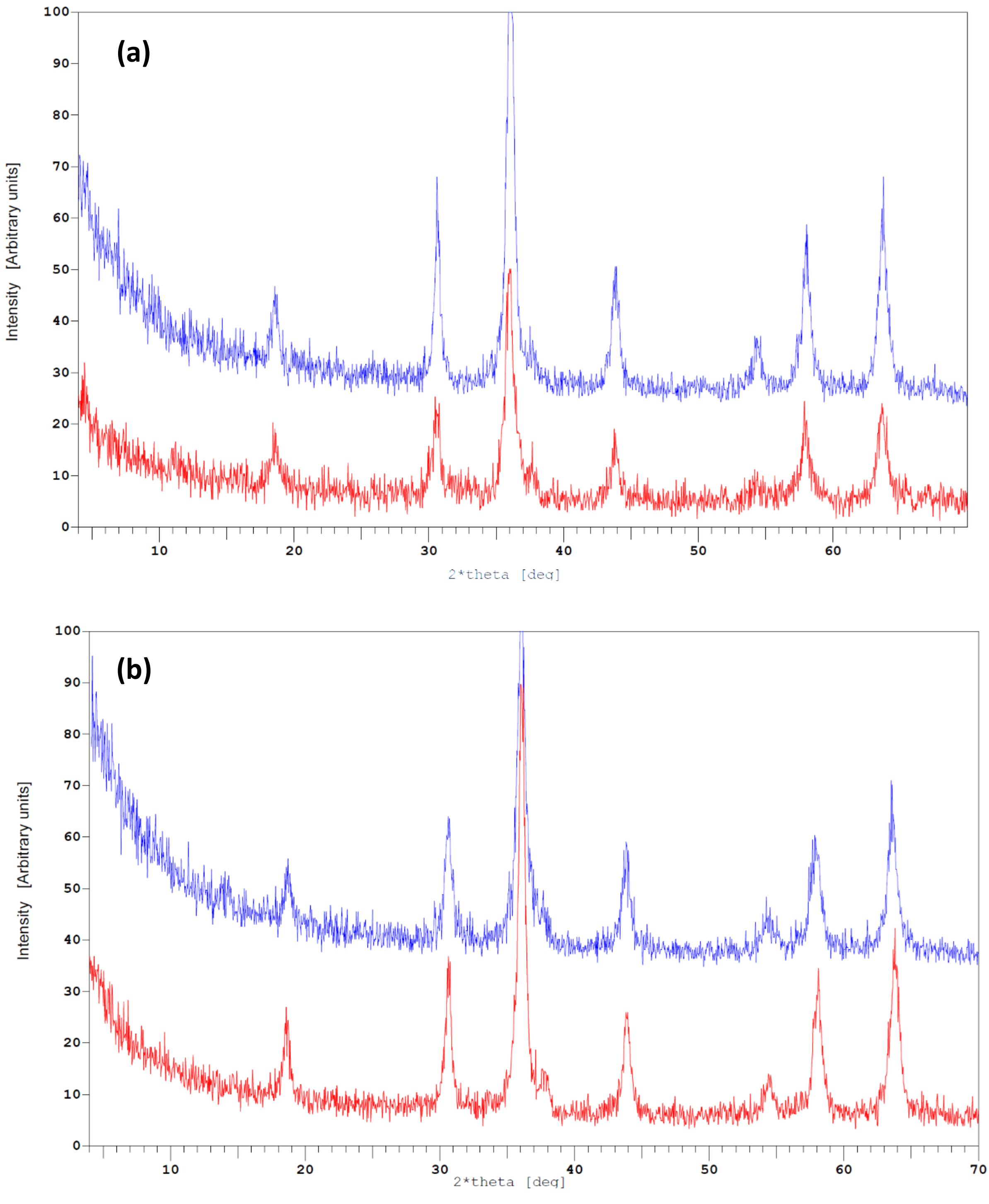

Figure S36. The PXRD of the *CrO_4-Cl-500-a* **(a)** and *CrO_4-CrO_4-500-a* **(b)** before (marked with red) and after (marked with blue) the catalytic reactions.

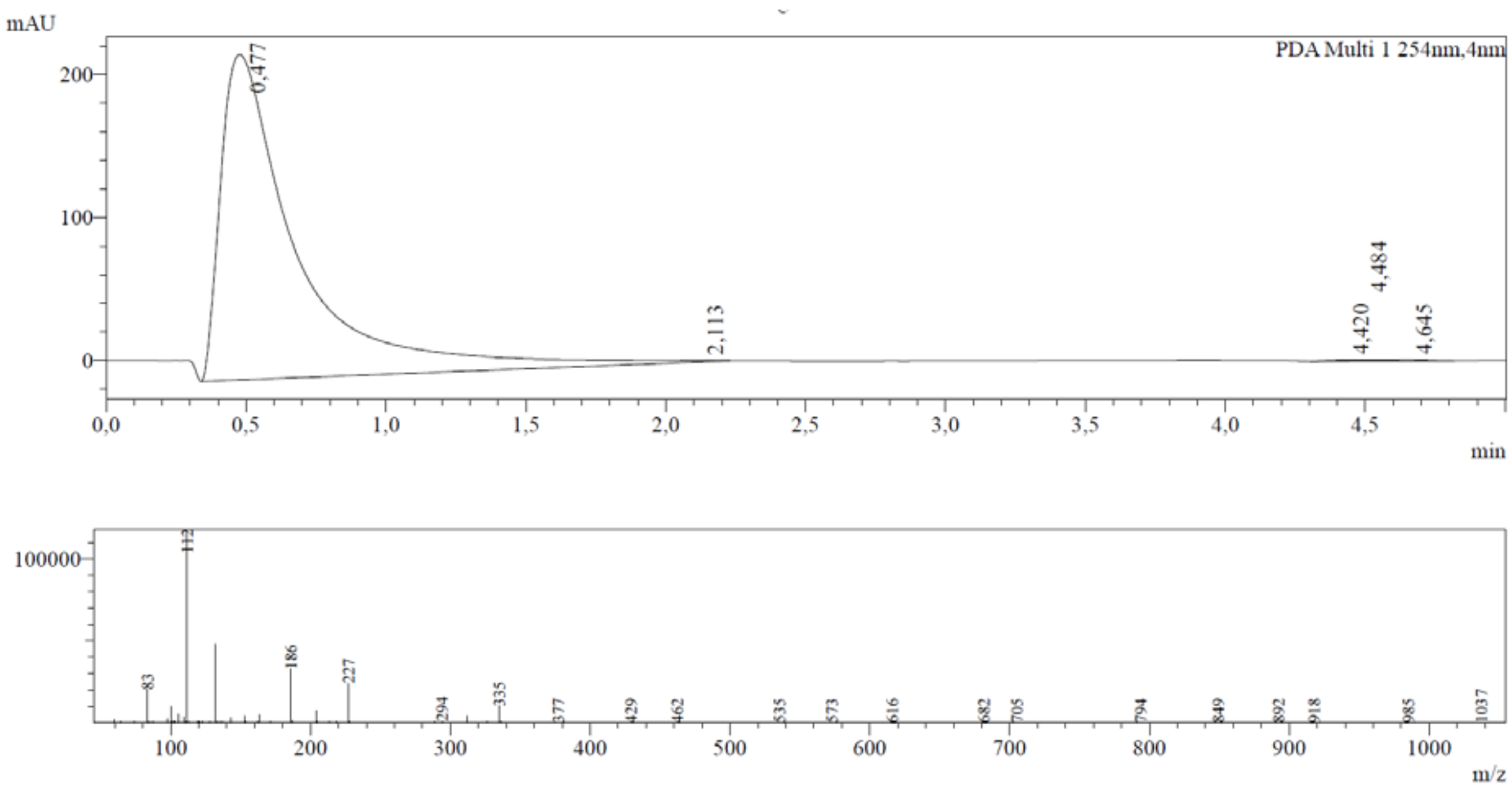

Figure S37. The chromatogram and the MS results of the HPLC-MS measurements of the RhB decomposition process.

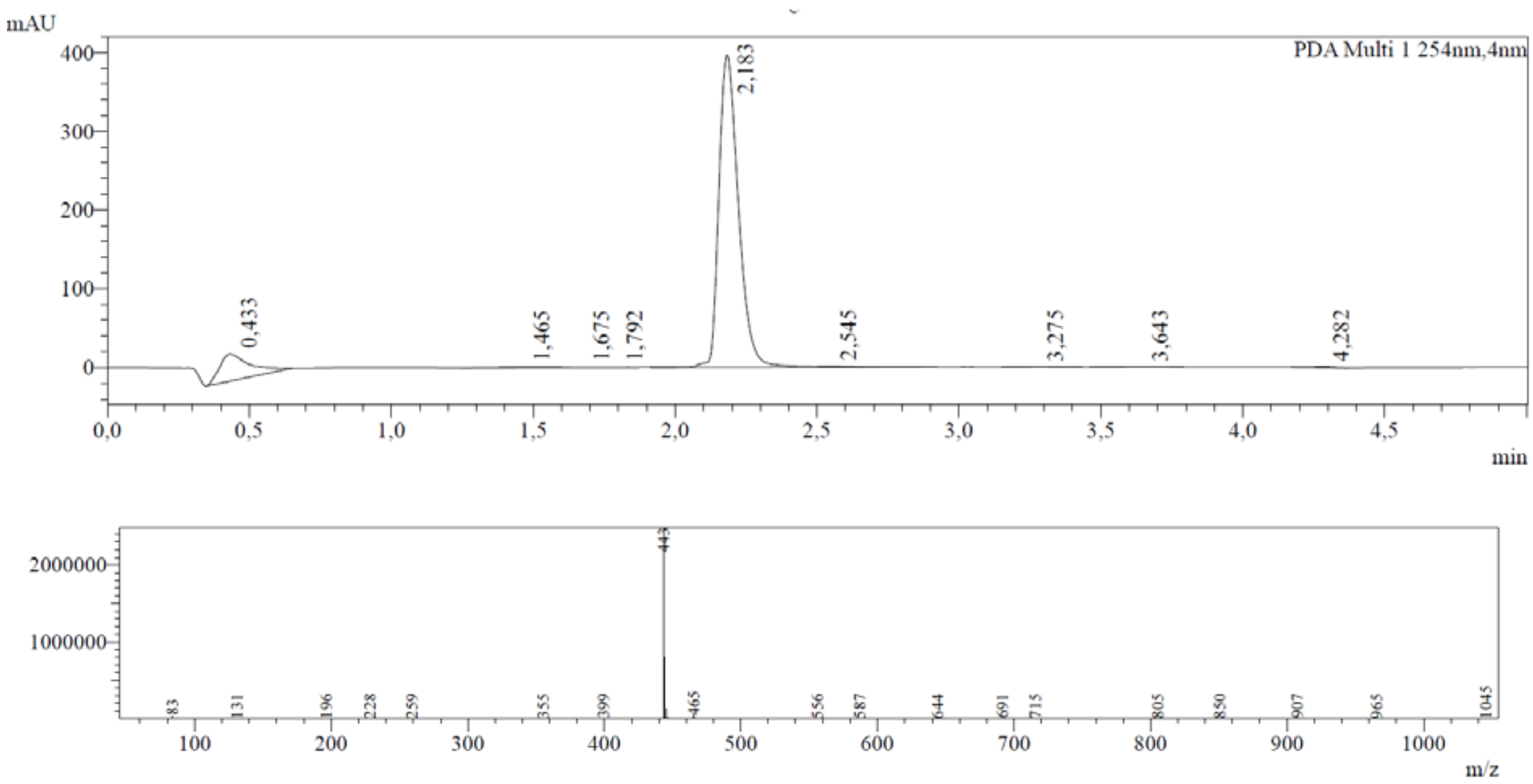

Figure S38. The chromatogram and the MS results of the HPLC-MS measurements of the RhB.

Text S5. Detailed Experimental descriptions

Ammonia (25 % aq. solution), hydrochloric acid (32 % aq. solution), sodium hydrogen carbonate, ammonium carbonate, cobalt(II) nitrate hexahydrate, silver nitrate, potassium chromate, ethanol, benzene, diethyl ether, chloroform, toluene, D_2O and other analytical reagents were supplied by Deuton-X Ltd (Érd, Hungary).

The preparation of $[Co(NH_3)_5Cl]Cl_2$

The [chloridopentaamminecobalt(III)] chloride precursor and the target compounds were prepared according to the slightly modified method of Briggs[26]. 122 g (0.42 mol) of cobalt(II) nitrate hexahydrate was dissolved in 250 mL of water and heated up to 60 °C. 71g (0.84 mol) of $NaHCO_3$ was dissolved in 700 mL of hot water, then this solution was added slowly to avoid intense foaming under intensive stirring. The mixture was filtered off, the precipitate was washed with cold water, ethanol, and diethyl ether, dried in open air overnight, then in an oven at 70 °C for half a day. The yield was 46 g (0.42 mol, 92 %). Cobalt(II) carbonate (20 g, 0.17 mol) was dissolved in 40 mL of 32 % hydrochloric acid and 50 mL of water, and a solution containing 5,6 g (0,58 mol) $(NH_3)_2CO_3$ in 180 mL of water and 180 ml of 25 % aq. NH_3 was added with the formation of a dark purple solution. The solution was heated up to 60 °C and 30 mL of 30 % aq. H_2O_2 was added dropwise to control the intensive foaming, then the solution was evaporated to 380 mL, then a 32 % aq. HCl solution was added dropwise until the gas evolution ceased (35 mL). A new portion (16 mL) of conc. NH_3 solution was added, and the solution was diluted with water up to 500 mL final volume, then boiled for an hour to evaporate, 150 mL of 32 % aq. HCl was added, and evaporated for a further 30 min. The solution was left to cool, then the precipitate was filtered off, washed with cold water, ethanol, and Et_2O, and dried in a d ccator. The yield of the reddish-purple powder was 20.1 g (0.08 mol), 47 % of the theoretical amount. Its solubility in water was found to be 9.960 g/L (23 °C), the pH of the saturated and 10-fold diluted solutions was found to be 3.54 and 4.01, and the electrolytic conductivity was 8.20 and 1.08 mS (23 °C), respectively.

The preparation of $[Co(NH_3)_5Cl]Cl_2$

$[Co(NH_3)_5Cl]Cl_2$ (20.1 g (0,08 mol) was dissolved in a mixture of 550 mL water and 55 mL 25 % aq. NH_3, then the mixture was heated up to 70 °C. The solution was neutralized (approximately the same amount of 20 % aq. HCl had to be added as ammonia solution). 12.8 g (0.066 mol) of K_2CrO_4 dissolved in 100 mL of water was added to the solution under mixing, then the mixture was allowed to cool. The precipitate was filtered off, washed with ice-cold water, ethanol, and diethyl ether, and dried in a d ccator. The mass of the brownish-red needles was found to be 7.4 g (0.0236 mol), that is 29.5 % of the theoretical amount.

The preparation of $[Co(NH_3)_5CrO_4]_2CrO_4\cdot 4H_2O$ (compound 2)

0.029 mol (9 g) of $[Co(NH_3)_5CrO_4]Cl\cdot H_2O$ was dissolved in 500 ml water at 70 °C. Freshly prepared silver(I) chromate precipitate (prepared by mixing 6 g (0.35 mol) of $AgNO_3$ in 20 mL of water with 3 g (0.15 mol) K_2CrO_4) in 20 mL of water) was added to the solution with stirring for 5 min. The white precipitate (AgCl) was filtered off, and the solution was allowed to cool. The precipitate was washed with ice-cold water, ethanol, and diethyl ether, and dried in a d ccator. The yield of compound **2** was 43.3 % (4.3 g, 0.0062 mol).

The decomposition of $[Co(NH_3)_5CrO_4]_2CrO_4\cdot 4H_2O$ (compound 2)

The oxide catalysts are prepared in tubular laboratory furnaces in both oxidative and inert (N_2) atmosphere with a heating rate of 20 $min\cdot s^{-1}$.
The measurement conditions with the PXRD (Philips PW Bragg-Brentano goniometer, 25 °C, graphite monochromator, 40 kV, 35 mA, CuKa source), the IR and far-IR (Bruker Tensor 27 Platinum, ATR, 4000-400 cm^{-1} and Biorad-Digilab FTS-30-FIR, 500-100 cm^{-1}, ATR, respectively), Raman (Horiba Jobin-Yvon LabRAM microspectrometer, lasers at

532 nm and 785 nm, Olympus BXC-40 microscope, at 25, -80 and -150 °C) and UV-Vis(Unicam UV500, 600-400 nm), the surface characterization (SEM, Zeiss EVO 40, 20 kV voltage, and BET, Autosorb IC) techniques, and the thermal studies (TG-DSC-MS (STD2960, Hiden Analytical HPR-20/QIC, 30-800 °C, 20 °/min), cryo-DSC (Setaram DSC92)), magnetic measurements (Quantum D gn MPMS-5S SQUID) and XPS (Kratos XSAM 800, Mg Kα1,2 excitation, 1253.6 eV, fixed analyzer transmission mode), were described in our previous papers. [27–30]

The single crystal X-ray diffraction

Single-crystal X-ray diffraction measurement of compound **1** was performed at 100 K on a RIGAKU RAXIS-RAPID diffractometer equipped with a graphite monochromator using MoKα radiation. Crystal data and details of the structure determination and refinement are listed in Table 1 and Tables S1-S11. Rigaku RAPID-AUTO software was used for data collection and data reduction. Olex2[31] and Mercury[32] were used for molecular graphics and analysing crystal packing. The structure was solved using the software SIR2014[33]. Refinement was carried out using Olex2[31] and SHELXL[34] with the full-matrix least-squares method on F^2. All non-hydrogen atoms were refined anisotropically. Hydrogen atoms were found in difference Fourier maps, and their positions were refined by the riding model except for hydrogens of the water molecule (H4A and H4B attached to O4). The positions of these hydrogens did not converge during refinement; they were fixed in the positions that resulted in the lowest R_1 value using "AFIX 1" instructions. Analytical absorption correction was applied to the data.
Single-crystal X-ray diffraction measurement of compound **2** was performed on a Rigaku XTALab Synergy-R diffractometer using Cu *Kα* radiation (λ = 1.54184 Å) on a single crystal. Crystal data and details of the structure determination and refinement are listed in Table 1 and Tables S1-S11. CrysAlisPro v 1.171.42.58a was used for data collection and data reduction. Olex2[31] and Mercury[32] were used for molecular graphics and analysing crystal packing.
Data were collected at 100 K using 38 ω scans. A total of 4904 frames were collected (0.5° rotation, 0.1 s and 0.4 s exposure time/frame for the low and high Θ regions, respectively). The structure was solved using the intrinsic phasing method as implemented in SHELXT. Refinement was carried out using Olex2 and SHELXL with the full-matrix least-squares method on F^2. All non-hydrogen atoms were refined anisotropically. Hydrogen atoms were found in difference Fourier maps, and their positions were refined by the riding model. Analytical absorption correction was applied to the data. The Hirshfeld surface analysis was performed using CrystalExplorer 21.3.[35]
Crystallographic data of compound **1** and compound **2** were deposited to the Cambridge Structural Database[36] under accession numbers 2544732 and 2544731, respectively.
The optimization of structures was performed in the Gaussian 09 Program Package[37] starting from the crystallographic structures of the obtained complexes. The selected level of theory included the B3LYP functional[38] in conjunction with 6-311++G(d,p) basis set[39] for non-metallic atoms and LanL2DZ[40] for cobalt and chromate ions. This level of theory was previously used to predict structural parameters and describe intramolecular interactions in similar compounds[41–43]. The optimizations were performed without any geometrical constraints. The intramolecular interactions and the stability of compounds were assessed by the Quantum Theory of Atoms in Molecules (QTAIM) approach[44,45]. The QTAIM analysis was performed using the AIMAll program package[46] and .wfx files generated by Gaussian 09.[37]

The XAFS measurements

The measurements of XAFS (XANES/EXAFS) for the Cr-K and Co-K edges in transmission mode were carried out at beamline BL-12C of the High Energy Accelerator Research Organization (KEK, Tsukuba, Japan). Before the XAFS measurements, the appropriate sample amount was estimated using the SAMPLEM4M software. The mixtures of sample and boron nitride (BN, Special Grade, 028-02281) were pelletized by pressing

at 10 MPa for 1 min. The X-ray beam generated by the 2.5 GeV synchrotron was monochromatized using a Si(111) double crystal. The Cr-K and Co-K edge energies were approximately 5989 and 7709 eV, respectively, and the energy scale was calibrated using the corresponding metallic Cr and Co foils available at KEK. The front ionization chamber was filled with a gas mixture of N2 and Ar (30 vol% N2 and 70 vol% Ar), while the rear ionization chamber was filled with 100 vol% N2. The edge energies were determined from the maximum of the first derivative of the XANES spectra of the corresponding metallic reference foils. The Co K-edge XANES spectra were analysed over the energy range of 7680–7780 eV. The pre-edge range was set from −150 to −30 eV, while normalization was performed over 50–1000 eV. The EXAFS spectra in the form of the Fourier-transformed radial distribution function were obtained over the range between 3.0 and 11.0 Å-1 by multiplying the original signals at *k*3.

Referneces

1 B. F. Abrahams, M. R. Grace, B. F. Hoskins and P. A. Tregloan, *Inorg. Chim. Acta.*, 1991, **182**, 135–138.
2 R. Tsuchida, *Bull. Chem. Soc. Jpn.*, 1938, **13**, 436–450.
3 R. Tsuchida and M. Kobayashi, *Bull. Chem. Soc. Jpn.*, 1938, **13**, 471–480.
4 H. Yoneda and M. Kobayashi, *Nippon Kagaku Zasshi*, 1954, **75**, 1192–1195.
5 R. Coomber and W. P. Griffith, *J. Chem. Soc., A*, 1968, **1**, 1128–1131.
6 K. Nakamoto, *Infrared and Raman Spectra of Inorganic and Coordination Compounds: Part A: Theory and Applications in Inorganic Chemistry*, Wiley, 1st edn., 2008.
7 Y. Chen, D. H. Christensen, G. O. Sørensen, O. F. Nielsen and E. Pedersen, *J. Mol. Struct.*, 1993, **299**, 61–72.
8 Z. Tian, C. Zhu, J. Wang, Z. Xia, Y. Liu and S. Yuan, *J. Magn. Magn. Mater.*, 2015, **377**, 176–182.
9 A. Ahmad, S. Khan, M. S. Javed, S. Osman, H. Li, S. Majeed and R. Luque, *ACS Appl. Mater. Interfaces*, 2024, **16**, 6920–6930.
10 R. Madhu, A. Karmakar and S. Kundu, *Inorg. Chem.*, 2023, **62**, 2726–2737.
11 S. Fareed, N. Ahmmad, N. K. Almaymoni, M. Amami, H. M. Abo-Dief, M. Y. Awaji, R. U. Hassan and A. Kumar, *Ceram. Int.*, 2026, **52**, 22217–22226.
12 J. Chen, X. Zhang, H. Arandiyan, Y. Peng, H. Chang and J. Li, *Catal. Today*, 2013, **201**, 12–18.
13 Y. Benrighi, N. Nasrallah, T. Chaabane, V. Sivasankar, A. Darchen and O. Baaloudj, *Opt. Mater.*, 2021, **122**, Article ID: 111819.
14 D. Chaibeddra, M. Benamira, M. Colmont, H. Boulehbal, H. Lahmar, I. Avramova and M. Trari, *Inorg. Chem. Commun.*, 2023, **155**, Article ID: 111116.
15 R. Madhu, A. Karmakar and S. Kundu, *Inorg. Chem.*, 2023, **62**, 2726–2737.
16 M. I. Miranda-López, F. F. Contreras-Torres, D. Cavazos-Cavazos, P. F. Martínez-Ortiz, N. Pineda-Aguilar, M. B. Hernández and J. A. Aguilar-Martínez, *J. Phys. Chem. Solids*, 2023, **178**, Article ID: 111315.
17 J. Hu, W. Zhao, R. Hu, G. Chang, C. Li and L. Wang, *Mater. Res. Bull.*, 2014, **57**, 268–273.
18 J. González-Cobos, B. Mylonoyannis, G. Chai, W. Zhang, C. Tian, A. Kaddouri and S. Gil, *Appl. Catal. A: Gen.*, 2023, **657**, Article ID: 119162.
19 D.-R. Kong, Y. Gao, Y.-Y. Xin, B. Li, X.-F. Zhang, Z.-P. Deng, L.-H. Huo and S. Gao, *Ceram. Int.*, 2023, **49**, 19206–19215.
20 S. S. Manoharan, N. R. S. Kumar and K. C. Patil, *Mater. Res. Bull.*, 1990, **25**, 731–738.
21 S. S. Manoharan and K. C. Patil, *J. Am. Ceram. Soc.*, 1992, **75**, 1012–1015.
22 K. Manjunatha, S.-L. Yu, Y.-E. Wu, M.-K. Ho, T.-E. Hsu, Y.-T. Yu, B. Chethan, M. P. Ravikumar, S. Mohan, T.-T. Lin, Y.-R. Hsu, M.-C. Chen, H. Nagabhushana and S. Y. Wu, *Chem. Eng. J.*, 2026, **535**, Article ID: 175200.
23 D. Gingasu, I. Mindru, D. C. Culita, L. Patron, J. M. Calderon-Moreno, P. Osiceanu, S. Preda, O. Oprea, V. Parvulescu, V. Teodorescu and J. P. S. Walsh, *Mater. Res. Bull.*, 2015, **62**, 52–64.
24 H. J. Gao, S. F. Wang, L. M. Fang, G. A. Sun, X. P. Chen, S. N. Tang, H. Yang, G. Z. Sun and D. F. Li, *Mater. Today Chem.*, 2021, **22**, Article ID: 100593.
25 M. Edrissi and A. R. Keshavarz, *Nano-Micro Lett.*, 2012, **4**, 83–89.

26 S. H. C. Briggs, *J. Chem. Soc., Trans.*, 1919, **115**, 67–76.
27 K. A. Béres, Z. Homonnay, L. Kvitek, Z. Dürvanger, M. Kubikova, V. Harmat, F. Szilágyi, Z. Czégény, P. Németh, L. Bereczki, V. M. Petruševski, M. Pápai, A. Farkas and L. Kótai, *Inorg. Chem.*, 2022, **61**, 14403–14418.
28 K. A. Béres, Z. Homonnay, L. Bereczki, V. M. Petruševski, A. Farkas, Z. Czégény, P. Németh, P. Pekker, F. Béres-Szilágyi, T. Stryšovský, L. Kvitek, Á. Gömöry and L. Kótai, *Inorg. Chem.*, 2025, **64**, 3427–3444.
29 K. A. Béres, Z. Dürvanger, Z. Homonnay, L. Bereczki, B. Barta Holló, A. Farkas, V. M. Petruševski and L. Kótai, *Inorganics*, 2024, **12**, 94–122.
30 L. Kótai, K. Lázár, L. F. Kiss and K. Szentmihályi, *Molecules*, 2024, **29**, 890–905.
31 O. V. Dolomanov, L. J. Bourhis, R. J. Gildea, J. A. K. Howard and H. Puschmann, *J. Appl. Crystallogr.*, 2009, **42**, 339–341.
32 C. F. Macrae, I. Sovago, S. J. Cottrell, P. T. A. Galek, P. McCabe, E. Pidcock, M. Platings, G. P. Shields, J. S. Stevens, M. Towler and P. A. Wood, *J. Appl. Crystallogr.*, 2020, **53**, 226–235.
33 M. C. Burla, R. Caliandro, B. Carrozzini, G. L. Cascarano, C. Cuocci, C. Giacovazzo, M. Mallamo, A. Mazzone and G. Polidori, *J. Appl. Crystallogr.*, 2015, **48**, 306–306.
34 G. M. Sheldrick, *Acta Crystallogr., Sect. C.*, 2015, **71**, 3–3.
35 P. R. Spackman, M. J. Turner, J. J. McKinnon, S. K. Wolff, D. J. Grimwood, D. Jayatilaka and M. A. Spackman, *J. Appl. Crystallogr.*, 2021, **54**, 1006–1011.
36 C. R. Groom, I. J. Bruno, M. P. Lightfoot and S. C. Ward, *Acta Crystallogr. B Struct. Sci. Cryst. Eng. Mater.*, 2016, **72**, 171–179.
37 M. J. Frisch, Gaussian 09, Revision D. 01/Gaussian (versions 09, Revision d. 01) Gaussian. Inc., Wallingford 2009.
38 A. D. Becke, *J. Chem. Phys.*, 1993, **98**, 5648–5652.
39 T. H. Dunning, *J. Chem. Phys.*, 1989, **90**, 1007–1023.
40 W. R. Wadt and P. J. Hay, *J. Chem. Phys.*, 1985, **82**, 284–298.
41 G. A. El-Inany, H. S. Seleem, B. A. El-Shetary, H. F. El-Shafiy, A. I. Nabeel, A. Madyan and M. Shebl, *Inorg. Chem. Commun.*, 2024, **160**, Article ID: 111888.
42 Y. A. A. Alghuwainem, H. M. A. El-Lateef, M. M. Khalaf, A. A. Amer, A. A. Abdelhamid, A. A. Alzharani, A. Alfarsi, S. Shaaban, M. Gouda and A. Abdou, *IJMS*, 2022, **23**, 15614–15634.
43 R. S. Sarkar, S. Banerjee and S. Chattopadhyay, *Inorg. Chim. Acta.*, 2025, **581**, Article ID: 122618.
44 R. F. W. Bader, *Acc. Chem. Res.*, 1985, **18**, 9–15.
45 R. F. W. Bader, T. S. Slee, D. Cremer and E. Kraka, *J. Am. Chem. Soc.*, 1983, **105**, 5061–5068.
46 T. A. Keith, TK Gristmill Software (version 17.01.25) aim.tkgristmill.com 2017.
